# Associations of Semaglutide With Skeletal Outcomes in People With Obesity, With and Without Type 2 Diabetes: A Target Trial Emulation

**DOI:** 10.1111/dom.70786

**Published:** 2026-04-20

**Authors:** Yu‐Nan Huang, Min‐Yu Tsou, Pin‐Hung Li, Jo‐Ching Chen, Shao‐Chia Chen, Hao‐I Hsieh, Gideon Meyerowitz‐Katz, Yen‐Liang Liu, Pen‐Hua Su

**Affiliations:** ^1^ Division of Genetics and Endocrinology, Department of Pediatrics Chung Shan Medical University Hospital Taichung Taiwan; ^2^ School of Medicine, Chung Shan Medical University Taichung Taiwan; ^3^ Cancer Biology and Precision Therapeutics Center, China Medical University Taichung Taiwan; ^4^ Institute of Medicine, Chung Shan Medical University Taichung Taiwan; ^5^ School of Medicine, College of Medicine, National Taiwan University Taipei Taiwan; ^6^ Department of Education National Taiwan University Hospital Taipei Taiwan; ^7^ School of Health and Society, University of Wollongong Wollongong Australia; ^8^ Master Program for Biomedical Engineering China Medical University Taichung Taiwan

**Keywords:** GLP‐1 receptor agonists, major osteoporotic fractures, obesity, osteoporosis, semaglutide, type 2 diabetes

## Abstract

**Aims:**

To evaluate the associations between semaglutide initiation and long‐term skeletal outcomes in people with obesity, stratified by type 2 diabetes (T2D) status, using target trial emulation.

**Materials and Methods:**

This retrospective cohort study used the TriNetX federated electronic health record network. People with obesity initiating semaglutide were matched 1:1 via propensity score matching (215 covariates) to those initiating active comparators or usual care. The with‐T2D cohort (*n* = 19 824–93 519 matched pairs per comparison) was followed for 3 years; the without‐T2D cohort (*n* = 10 323–56 225 pairs) for 2 years. The primary outcome was major osteoporotic fracture (MOF). Secondary outcomes included osteoporosis diagnosis; exploratory outcomes included osteoarthritis and gout.

**Results:**

In the with‐T2D cohort, semaglutide initiation was associated with a lower risk of MOF compared with empagliflozin (HR 0.69; 95% CI 0.61–0.77), glipizide (HR 0.72; 0.63–0.83) and usual care (HR 0.84; 0.76–0.93). In the without‐T2D cohort, no significant associations with MOF were observed across any comparison (all *p* > 0.05). Osteoporosis risk did not differ significantly in most comparisons in either cohort. Exploratory analyses of osteoarthritis and gout showed inconsistent patterns across comparators.

**Conclusions:**

Among people with obesity and T2D, semaglutide initiation was associated with a lower risk of MOF over 3 years compared with other glucose‐lowering agents and usual care. This association was not observed in people with obesity without T2D. These findings support further investigation of the skeletal effects of GLP‐1 receptor agonists.

## 1 Introduction

Obesity and type 2 diabetes (T2D) are increasingly prevalent worldwide [1, 2]. Beyond well‐documented cardiovascular complications, mounting evidence suggests these metabolic conditions significantly impact bone health [3, 4]. Excess body weight increases mechanical stress on weight‐bearing joints, while diabetes‐related metabolic perturbations may alter bone density and microarchitecture [5, 6, 7]. These concerns have sparked growing interest in understanding how contemporary therapeutic agents influence long‐term skeletal outcomes [8, 9].

Glucagon‐like peptide‐1 receptor agonists (GLP‐1 RAs), with semaglutide at the forefront, have revolutionised clinical approaches to adiposity reduction and blood glucose regulation in recent years [10, 11]. While clinical trials have demonstrated remarkable efficacy in these primary outcomes, the role of GLP‐1 signalling in bone metabolism remains incompletely understood [12]. Nevertheless, long‐term clinical data on skeletal safety remain scarce, particularly in real‐world cohorts.

Few head‐to‐head evaluations of GLP‐1 RAs versus alternative weight‐loss or glucose‐lowering drugs have reported skeletal outcomes over longer follow‐up. Prior studies also rarely distinguish people living with obesity alone from those with concomitant T2D, and multi‐domain skeletal assessments are uncommon.

We therefore prioritised major osteoporotic fracture (MOF) as the primary endpoint because it summarises clinically relevant fracture burden [13]. This study aimed to characterise the associations between semaglutide initiation and long‐term skeletal outcomes in people with obesity, stratified by T2D status, using a target trial emulation framework.

## 2 Methods

### 2.1 Data Source and Ethics

This retrospective study used the TriNetX US Collaborative Network, a federated database of de‐identified records from approximately 120 million patients across 65 healthcare organisations in the United States, containing diagnoses (ICD‐10‐CM), procedures, prescriptions and laboratory data.

The Institutional Review Board at Chung Shan Medical University Hospital approved this study (CS2‐24004, CS2‐24100). TriNetX provides only de‐identified aggregate data; accordingly, the IRB waived consent under 45 CFR 46.104 (d) (4). Reporting followed STROBE guidelines.

### 2.2 Study Design and Target Trial Emulation

We emulated two parallel target trials in TriNetX to compare skeletal outcomes after initiation of semaglutide in people living with obesity, stratified by type 2 diabetes (T2D) status. Eligibility windows were May 13, 2022 to Feb 28, 2026 for obesity with T2D; Nov 8, 2023 to Feb 28, 2026 for obesity without T2D. Three comparator categories are used throughout the manuscript. Active comparators denote individual drugs compared separately with semaglutide: empagliflozin, sitagliptin and glipizide in the T2D cohort; naltrexone–bupropion, phentermine and phentermine–topiramate in the non‐T2D cohort. Usual care denotes patients who did not initiate any index or comparator medication and were treated with metformin in both cohorts. In both cohorts, usual care was defined as metformin use without initiation of semaglutide or any active comparator medication. Pooled active comparators, reported only in Supporting Information, combine all individual active comparators within each cohort into a single reference group. These windows were aligned with the respective FDA approval dates (Ozempic, December 2017; Wegovy, June 2021). An approval‐aligned sensitivity analysis restricted each comparison to the later drug's approval date. The first qualifying prescription defined the index date (*T*_0_). A new‐user design required no prescription for the index drug or same‐class medication during the 6 months before *T*_0_. A directed acyclic graph (Figure S1) guided covariate selection, separating confounders from mediators. Post‐baseline mediators (weight change, glycemic trajectories) were not adjusted for to avoid blocking the pathway of interest.

For semaglutide, active comparators were selected based on prescribing frequency within each stratum. In the T2D cohort, empagliflozin (SGLT2 inhibitor), sitagliptin (DPP‐4 inhibitor) and glipizide (sulfonylurea) were the three most commonly started second‐line glucose‐lowering agents during the study period; usual care was defined as stable metformin monotherapy without initiation of any index or comparator medication. In the non‐T2D cohort, naltrexone–bupropion, phentermine and phentermine–topiramate were selected as FDA‐approved anti‐obesity medications with the largest user base; usual care was defined as metformin treatment without initiation of semaglutide or any active comparator medication. Each comparator carries a distinct skeletal risk profile. Early CANVAS trial data raised concern about fractures associated with canagliflozin, though subsequent studies of empagliflozin and dapagliflozin did not replicate this signal [14, 15]. Phentermine is generally prescribed for shorter durations to younger patients with fewer comorbidities [16]. These differences in patient profiles were addressed through 1:1 propensity score matching within each comparison. We conducted parallel active‐comparator analyses, including head‐to‐head contrasts with semaglutide and additional comparisons with selected glucose‐lowering or weight‐lowering agents. We also evaluated pooled comparator strategies within each cohort, defined as initiation of any prespecified active comparator in that stratum, to summarise associations against a broader treatment mix. All primary analyses report individual drug comparisons.

Following target trial emulation guidance [17, 18, 19], we prespecified eligibility, treatment strategies, assignment procedures, follow‐up, outcomes, contrasts and the analysis plan (Table S1). All analyses were interpreted as comparative associations under real‐world treatment initiation and follow‐up patterns, rather than as causal effects.

### 2.3 Cohort Construction and Exposure Definition

**FIGURE 1:**
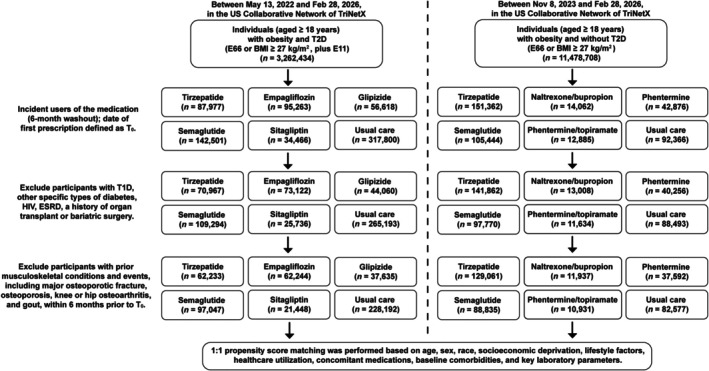
Study flow diagram. People with obesity (ICD‐10 E66 plus BMI ≥ 27 kg/m^2^) were identified from the TriNetX US Collaborative Network between May 2022 and February 2026 (with T2D) or November 2023 and February 2026 (without T2D). The index date (*T*_0_) was the first qualifying prescription. Sequential exclusions removed patients with type 1 or other specified diabetes, HIV, end‐stage renal disease, organ transplantation, bariatric surgery, prior musculoskeletal outcomes within 6 months before *T*_0_, and recent use of study drugs. Final matched cohorts were created using 1:1 propensity score matching based on age, sex, race, socioeconomic deprivation, lifestyle factors, healthcare utilisation, concomitant medications, baseline comorbidities and key laboratory parameters. PSM, propensity score matching; *T*_0_, index date; T2D, type 2 diabetes.

In each cohort, exposure groups were defined by initiation of semaglutide identified from prescription records. Active comparators were defined by initiation of the corresponding comparator medication. For pooled comparator analyses, the comparator group comprised initiators of any prespecified active comparator within that cohort (Figure 1). The index date was the first prescription within the observation window. Within the usual care groups, metformin use was part of the operational definition. Baseline use of these drugs was captured from the supplemental medication records and included as indicator variables in the propensity score.

Exclusions included other/unspecified diabetes (E08, E09, E13), type 1 diabetes (E10), prior bariatric surgery or organ transplantation, HIV and end‐stage renal disease. The primary analysis followed an intention‐to‐treat approach. Bariatric surgery was not a censoring event because it is a post‐baseline intervention potentially influenced by the index treatment; censoring at surgery would introduce informative censoring bias if surgical probability differs between groups. Under the primary ITT estimand, follow‐up started at T0 and continued regardless of treatment discontinuation, switching or augmentation, and ended at the first occurrence of the outcome, death, loss to follow‐up or administrative end of follow‐up. Prescription records capture orders not confirmed dispensing; resultant misclassification would attenuate ITT estimates toward the null. The per‐protocol analysis partially addresses this.

### 2.4 Outcome Assessment

The primary endpoint was MOF, defined as a composite of hip (S72), clinical vertebral (S32.0–S32.2), distal radius/ulna (S52.5–S52.6), proximal humerus (S42.2–S42.3) and fragility fractures (M80). Secondary fracture outcomes included individual MOF components. The secondary outcome was incident osteoporosis (M80–M81). Exploratory outcomes were knee osteoarthritis (M17), hip osteoarthritis (M16) and gout (M10), disorders of bone density and structure (M85.8–M85.9), bone‐active medication use (alendronate, zoledronic acid, denosumab), healthcare utilisation (outpatient, emergency, inpatient) and described longitudinal BMI and HbA1c trajectories under the ITT principle, with measurements attributed to the original treatment group regardless of subsequent treatment changes. These trajectories reflect the combined influence of the index drug, switching, dose adjustment and concomitant medications during follow‐up. Knee osteoarthritis, hip osteoarthritis and gout were exploratory outcomes. A validated hip fracture endpoint, defined by hip fracture diagnosis combined with inpatient surgical procedure codes, was analysed as a sensitivity analysis. To probe residual confounding, we included negative control outcomes with no expected relation to exposure: dog bites, ganglion cysts, adhesive capsulitis of shoulder, blepharitis, hernias and skin cancer (Supporting Information).

### 2.5 Covariates

We adjusted for 215 covariates to balance treatment groups. These included demographics (age, sex, race, socioeconomic status), lifestyle factors (nicotine dependence, alcohol‐related disorders), comorbidities (hypertension, ischemic heart disease, heart failure, hyperlipidemia, acute and chronic kidney disease), concomitant therapies (insulin, metformin, SGLT2 inhibitors, thiazolidinediones, sulfonylureas, DPP‐4 inhibitors; ACE inhibitors, ARBs, beta‐blockers, calcium‐channel blockers, statins; bisphosphonates), laboratory measures (BMI, HbA1c, eGFR, proteinuria, lipid profile), systemic glucocorticoid prescriptions within 12 months before index, DXA utilisation, healthcare utilisation intensity (emergency, ambulatory and inpatient visits during 12 months before index), fall history and prior traumatic fracture. Comorbidities were ascertained via ICD‐10 codes, medications from prescription records and labs from structured EHR fields. These variables entered the propensity‐score matching to improve balance (Supporting Information).

### 2.6 Statistical Analysis

Propensity score matching was used because exact matching on 215 covariates (including continuous measures such as BMI, HbA1c, eGFR and lipid values) was not feasible. Each contrast was analysed in an independently matched cohort using 1:1 nearest‐neighbour matching with a calliper of 0.2 standard deviations of the logit propensity score; balance was confirmed by standardised mean differences below 0.10. Cox proportional hazards models estimated hazard ratios with 95% confidence intervals. The Benjamini–Hochberg procedure controlled the false discovery rate across all comparisons; both raw and adjusted *p*‐values are reported. E‐values were calculated for selected significant associations. The primary prespecified hypothesis was that semaglutide initiation would be associated with lower MOF hazard compared with active comparators in the T2D cohort. Osteoporosis was prespecified as the secondary outcome. Non‐T2D comparisons and exploratory outcomes (knee OA, hip OA, gout) were interpreted as exploratory. Subgroup analyses were hypothesis‐generating.

Per‐protocol (PP) analyses were performed by restricting to patients persistent on their assigned treatment across cumulative 75‐day refill intervals. Grace periods were tested: 75 days (30‐day prescription plus 45‐day permissible gap) [20]. The proportion of participants remaining on index treatment at each grace period threshold was reported for all treatment groups. The ITT analysis was retained as the primary approach because it reflects the clinical question of treatment initiation and avoids informative censoring that may arise when discontinuation is related to the outcome. Osteoporosis medication use (alendronate, zoledronic acid, denosumab) and healthcare utilisation were analysed as time‐to‐event outcomes using the same Cox proportional hazards approach in each matched cohort. Longitudinal changes in BMI and HbA1c were tracked from the index date across the follow‐up period using the TriNetX analytics module.

Pre‐specified subgroups included eGFR (≥ 45 vs. < 45 mL/min/1.73 m^2^), HbA1c (≥ 7% vs. < 7%), BMI categories (< 30, 30–34.9, 35–39.9, ≥ 40 kg/m^2^), cardiovascular comorbidities (hypertension, heart failure, ischemic heart disease), proteinuria and background therapy (insulin, metformin, renin–angiotensin system inhibitors). Sensitivity analyses evaluated a class‐based sensitivity analysis by replacing semaglutide with any GLP‐1 RA (semaglutide, liraglutide, dulaglutide, exenatide) under the same specification. Missing data were handled without imputation. The proportions of missing data for key continuous covariates before propensity score matching are reported in Table S2. To assess the robustness of fracture findings to outcome misclassification, two additional analyses were performed. First, the fracture endpoint was restricted to hip fracture confirmed by surgical procedure codes (CPT 27235, 27 244, 27 245; ICD‐10‐PCS 0QS6 and 0QS7), a validated endpoint with a reported positive predictive value exceeding 95% in administrative databases [21]. Second, site‐specific fracture outcomes (hip, clinical vertebral, distal radius/ulna, proximal humerus) were analysed individually to evaluate whether the composite MOF association was consistent across fracture types. To address the potential influence of differential mortality as a competing event, we examined a composite endpoint of MOF plus all‐cause mortality across all comparisons. Complete‐case analysis was used; sensitivity sets with alternative completeness rules were also examined.

Analyses were run on the TriNetX platform (Java 11.0.16), with statistics in R 4.0.2 and visualisation in Python 3.7 and Prism 10.0. Cell counts < 10 were rounded to the nearest 10 to protect privacy.

## 3 Results

### 3.1 Baseline Characteristics and Propensity Score Matching

Our study included two populations with obesity: those with T2D, compared against glucose‐lowering agents or usual care over 3 years, and those without T2D, compared against anti‐obesity medications or usual care over 2 years (Figure 1; Tables S3–S10). Across the nine T2D comparisons, matched cohorts ranged from 19 824 to 93 519 pairs per arm. After matching, mean age ranged from 55.1 to 62.5 years, and the proportion of female participants varied from 43.0% to 59.2%, reflecting the different sex distributions of each comparator population. Mean BMI fell between 34.1 and 38.2 kg/m^2^, mean HbA1c between 7.1% and 7.5%, and all post‐matching SMDs were below 0.10.

### 3.2 Major Osteoporotic Fracture Outcomes

**FIGURE 2:**
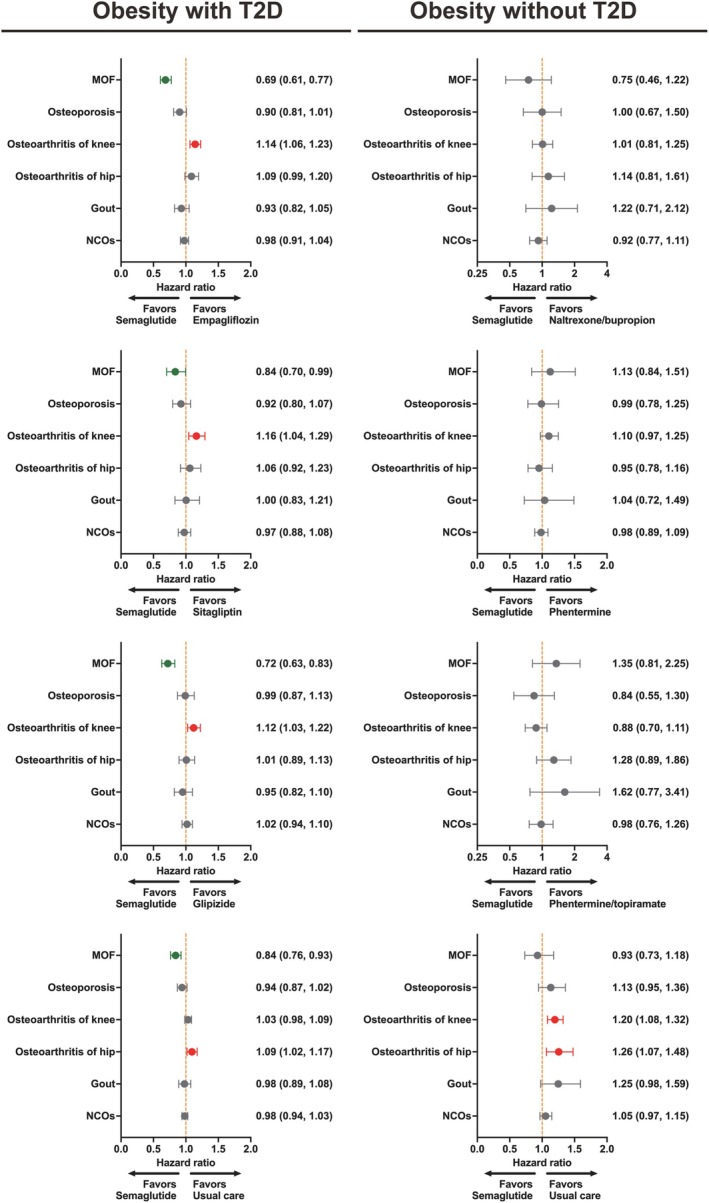
Forest plots of hazard ratios for skeletal outcomes: Semaglutide versus comparators. Left panels: obesity with T2D cohort (3‐year follow‐up); right panels: obesity without T2D cohort (2‐year follow‐up). Each row within a panel represents one comparator. Hazard ratios (diamonds) with 95% confidence intervals (horizontal lines) were estimated using Cox proportional hazards models after 1:1 propensity score matching. Green diamonds indicate a statistically significant association favouring semaglutide (HR < 1.0); red diamonds indicate a statistically significant association favouring the comparator (HR > 1.0); black diamonds indicate non‐significant results. The dashed vertical line at HR = 1.0 represents no difference. Primary outcome: Major osteoporotic fracture (MOF). Secondary outcome: Osteoporosis. Exploratory outcomes: Knee osteoarthritis, hip osteoarthritis, gout. NCOs, negative control outcomes (prespecified falsification endpoints included to assess residual confounding; HRs near 1.0 support the validity of the primary findings). CI, confidence interval; HR, hazard ratio; T2D, type 2 diabetes.

We examined composite MOF across all treatment comparisons (Table 1 and Figure 2). In the T2D cohort over 3 years, semaglutide was associated with lower MOF hazards versus empagliflozin (HR 0.69, 95% CI: 0.61–0.77), glipizide (HR 0.72, 95% CI: 0.63–0.83) and usual care (HR 0.84, 95% CI: 0.76–0.93), all of which remained significant after FDR correction (Table 1). The semaglutide versus sitagliptin comparison showed a similar direction (HR 0.84, 95% CI: 0.70–0.99) and nominal significance before adjustment (raw *p* = 0.045), but it did not remain significant after FDR correction (adjusted *p* = 0.113). In the non‐T2D cohort over 2 years, none of the semaglutide comparisons reached statistical significance for MOF (Table 2).

**TABLE 1:** Three‐year hazard ratios for skeletal outcomes associated with semaglutide versus comparators in people with obesity and type 2 diabetes.

	Exposure cohort	Comparator cohort	Risk difference (95% CI)	ARD (%)	NNT	HR‐based RRR (%)	HR (95% CI)	*P* value	Adjusted *p* value (BH Method)	*E* value for HR	*E* value (CI limit closest to null)
Semaglutide vs. empagliflozin											
MOF	441/46 215 (1.0)	615/46 150 (1.3)	−0.004 (−0.005, −0.002)	−0.4	+143	+23.78	0.69 (0.61, 0.77)	< 0.001	< 0.001	2.28	1.90
Osteoporosis	633/45 884 (1.4)	669/45 811 (1.5)	−0.001 (−0.002, 0.001)	−0.1	N/A	N/A	0.90 (0.81, 1.01)	0.071	0.118	N/A	N/A
Osteoarthritis of knee	1607/40 740 (3.9)	1400/42 321 (3.3)	0.006 (0.004, 0.009)	+0.6	−81	−17.27	1.14 (1.06, 1.23)	< 0.001	< 0.001	1.55	1.32
Osteoarthritis of hip	872/44 885 (1.9)	778/45 310 (1.7)	0.002 (0.001, 0.004)	+0.2	N/A	N/A	1.09 (0.99, 1.20)	0.095	0.119	N/A	N/A
Gout	515/44 970 (1.1)	530/44 992 (1.2)	−0.000 (−0.002, 0.001)	0	N/A	N/A	0.93 (0.82, 1.05)	0.248	0.248	N/A	N/A
NCOs	1810/37 696 (4.8)	1809/38 636 (4.7)	0.001 (−0.002, 0.004)	+0.1	N/A	N/A	0.98 (0.91, 1.04)	0.472	N/A	N/A	N/A
Semaglutide vs. sitagliptin											
MOF	226/19 267 (1.2)	290/19 315 (1.5)	−0.003 (−0.006, −0.001)	−0.3	+250	+12.62	0.84 (0.70, 0.99)	0.045	0.113	1.68	1.06
Osteoporosis	325/18 942 (1.7)	376/18 988 (2.0)	−0.003 (−0.005, 0.000)	−0.3	N/A	N/A	0.92 (0.80, 1.07)	0.298	0.496	N/A	N/A
Osteoarthritis of knee	689/16 877 (4.1)	664/17 692 (3.8)	0.003 (−0.001, 0.007)	+0.3	−81	−15.63	1.16 (1.04, 1.29)	0.006	0.028	1.60	1.26
Osteoarthritis of hip	352/18 701 (1.9)	358/18 982 (1.9)	−0.000 (−0.003, 0.003)	0	N/A	N/A	1.06 (0.92, 1.23)	0.423	0.528	N/A	N/A
Gout	212/18 824 (1.1)	226/19 059 (1.2)	−0.001 (−0.003, 0.002)	−0.1	N/A	N/A	1.00 (0.83, 1.21)	0.972	0.972	N/A	N/A
NCOs	757/15 970 (4.7)	829/16 151 (5.1)	−0.004 (−0.009, 0.001)	−0.4	N/A	N/A	0.97 (0.88, 1.08)	0.604	N/A	N/A	N/A
Semaglutide vs. glipizide											
MOF	340/31 766 (1.1)	484/31 812 (1.5)	−0.005 (−0.006, −0.003)	−0.5	+122	+24.03	0.72 (0.63, 0.83)	< 0.001	< 0.001	2.12	1.70
Osteoporosis	451/31 465 (1.4)	465/31 658 (1.5)	−0.000 (−0.002, 0.002)	0	N/A	N/A	0.99 (0.87, 1.13)	0.887	0.898	N/A	N/A
Osteoarthritis of knee	1042/28 186 (3.7)	980/29 376 (3.3)	0.004 (0.001, 0.007)	+0.4	−491	−2.67	1.12 (1.03, 1.22)	0.011	0.027	1.49	1.19
Osteoarthritis of hip	539/30 915 (1.7)	552/31 331 (1.8)	−0.000 (−0.002, 0.002)	0	N/A	N/A	1.01 (0.89, 1.13)	0.898	0.898	N/A	N/A
Gout	347/31 041 (1.1)	373/31 353 (1.2)	−0.001 (−0.002, 0.001)	−0.1	N/A	N/A	0.95 (0.82, 1.10)	0.494	0.823	N/A	N/A
NCOs	1244/26 451 (4.7)	1248/27 046 (4.6)	0.001 (−0.003, 0.004)	+0.1	N/A	N/A	1.02 (0.94, 1.10)	0.662	N/A	N/A	N/A
Semaglutide vs. usual care											
MOF	763/91 468 (0.8)	952/91 607 (1.0)	−0.002 (−0.003, −0.001)	−0.2	+567	+8.38	0.84 (0.76, 0.93)	< 0.001	0.002	1.66	1.38
Osteoporosis	1178/90 771 (1.3)	1313/91 110 (1.4)	−0.001 (−0.003, −0.000)	−0.1	N/A	N/A	0.94 (0.87, 1.02)	0.127	0.212	N/A	N/A
Osteoarthritis of knee	3026/81 541 (3.7)	3107/83 887 (3.7)	0.000 (−0.002, 0.002)	0	N/A	N/A	1.03 (0.98, 1.09)	0.195	0.244	N/A	N/A
Osteoarthritis of hip	1542/89 320 (1.7)	1482/89 958 (1.6)	0.001 (−0.000, 0.002)	+0.1	−305	−9.71	1.09 (1.02, 1.17)	0.014	0.036	1.41	1.15
Gout	831/89 891 (0.9)	886/90 627 (1.0)	−0.001 (−0.001, 0.000)	−0.1	N/A	N/A	0.98 (0.89, 1.08)	0.644	0.644	N/A	N/A
NCOs	3461/76 004 (4.6)	3661/76 850 (4.8)	−0.002 (−0.004, 0.000)	−0.2	N/A	N/A	0.98 (0.94, 1.03)	0.464	N/A	N/A	N/A

*Note*: Data are presented as events/total patients (proportion). Absolute risk differences were derived from Kaplan–Meier cumulative incidence at 1080 days; NNT = 1/|ARD|, rounded upward, reported only for significant results. Hazard ratios with 95% confidence intervals were estimated using Cox proportional hazards models after 1:1 propensity score matching (215 covariates, calliper 0.2 SD). *p* values were adjusted using the Benjamini–Hochberg method. *E* values quantify the minimum strength of unmeasured confounding needed to explain away the observed association; reported only for significant results.

Abbreviations: ARD, absolute risk difference; BH, Benjamini–Hochberg; CI, confidence interval; HR, hazard ratio; MOF, major osteoporotic fracture; N/A, not applicable; NCO, negative control outcome; NNT, number needed to treat; RRR, relative risk reduction.

**TABLE 2:** Two‐year hazard ratios for skeletal outcomes associated with semaglutide versus comparators in people with obesity Without type 2 diabetes.

	Exposure cohort	Comparator cohort	Risk difference (95% CI)	ARD (%)	NNT	HR‐based RRR (%)	HR (95% CI)	*p*	Adjusted *p* (BH method)	*E* value for HR	*E* value (CI limit closest to null)
Semaglutide vs. naltrexone/bupropion											
MOF	30/10 141 (0.3)	35/10 114 (0.3)	−0.001 (−0.002, 0.001)	−0.1	N/A	N/A	0.75 (0.46, 1.22)	0.240	0.784	N/A	N/A
Osteoporosis	51/10 158 (0.5)	44/10 171 (0.4)	0.001 (−0.001, 0.003)	+0.1	N/A	N/A	1.00 (0.67, 1.50)	0.992	0.992	N/A	N/A
Osteoarthritis of knee	174/9470 (1.8)	153/9526 (1.6)	0.002 (−0.001, 0.006)	+0.2	N/A	N/A	1.01 (0.81, 1.25)	0.934	0.992	N/A	N/A
Osteoarthritis of hip	74/10 036 (0.7)	57/10 038 (0.6)	0.002 (−0.001, 0.004)	+0.2	N/A	N/A	1.14 (0.81, 1.61)	0.454	0.784	N/A	N/A
Gout	30/10 175 (0.3)	22/10 187 (0.2)	0.001 (−0.001, 0.002)	+0.1	N/A	N/A	1.22 (0.71, 2.12)	0.470	0.784	N/A	N/A
NCOs	226/8649 (2.6)	217/8753 (2.5)	0.001 (−0.003, 0.006)	+0.1	N/A	N/A	0.92 (0.77, 1.11)	0.395	N/A	N/A	N/A
Semaglutide vs. phentermine/topiramate											
MOF	37/10 689 (0.3)	25/10 686 (0.2)	0.001 (−0.000, 0.003)	+0.1	N/A	N/A	1.35 (0.81, 2.25)	0.242	0.348	N/A	N/A
Osteoporosis	39/10 710 (0.4)	43/10 711 (0.4)	−0.000 (−0.002, 0.001)	0	N/A	N/A	0.84 (0.55, 1.30)	0.436	0.436	N/A	N/A
Osteoarthritis of knee	138/10 187 (1.4)	146/10 168 (1.4)	−0.001 (−0.004, 0.002)	−0.1	N/A	N/A	0.88 (0.70, 1.11)	0.278	0.348	N/A	N/A
Osteoarthritis of hip	68/10 609 (0.6)	49/10 615 (0.5)	0.002 (−0.000, 0.004)	+0.2	N/A	N/A	1.28 (0.89, 1.86)	0.181	0.348	N/A	N/A
Gout	19/10 754 (0.2)	11/10 756 (0.1)	0.001 (−0.000, 0.002)	+0.1	N/A	N/A	1.62 (0.77, 3.41)	0.198	0.348	N/A	N/A
NCOs	122/9923 (1.2)	115/9923 (1.2)	0.001 (−0.002, 0.004)	+0.1	N/A	N/A	0.98 (0.76, 1.26)	0.865	N/A	N/A	N/A
Semaglutide vs. phentermine											
MOF	92/36 229 (0.3)	85/36 205 (0.2)	0.000 (−0.001, 0.001)	0	N/A	N/A	1.13 (0.84, 1.51)	0.433	0.927	N/A	N/A
Osteoporosis	133/36 288 (0.4)	140/36 274 (0.4)	−0.000 (−0.001, 0.001)	0	N/A	N/A	0.99 (0.78, 1.25)	0.927	0.927	N/A	N/A
Osteoarthritis of knee	502/34 555 (1.5)	478/34 819 (1.4)	0.001 (−0.001, 0.003)	+0.1	N/A	N/A	1.10 (0.97, 1.25)	0.126	0.632	N/A	N/A
Osteoarthritis of hip	192/36 024 (0.5)	210/36 068 (0.6)	−0.000 (−0.002, 0.001)	0	N/A	N/A	0.95 (0.78, 1.16)	0.622	0.927	N/A	N/A
Gout	59/36 348 (0.2)	59/36 386 (0.2)	0.000 (−0.001, 0.001)	0	N/A	N/A	1.04 (0.72, 1.49)	0.836	0.927	N/A	N/A
NCOs	708/32 568 (2.2)	744/32 434 (2.3)	−0.001 (−0.003, 0.001)	−0.1	N/A	N/A	0.98 (0.89, 1.09)	0.741	N/A	N/A	N/A
Semaglutide vs. usual care											
MOF	139/55 428 (0.3)	135/55 434 (0.2)	0.000 (−0.001, 0.001)	0	N/A	N/A	0.93 (0.73, 1.18)	0.540	0.540	N/A	N/A
Osteoporosis	264/55 353 (0.5)	212/55 452 (0.4)	0.001 (0.000, 0.002)	+0.1	N/A	N/A	1.13 (0.95, 1.36)	0.174	0.217	N/A	N/A
Osteoarthritis of knee	876/52 208 (1.7)	682/53 289 (1.3)	0.004 (0.003, 0.005)	+0.4	−292	−9.25	1.20 (1.08, 1.32)	< 0.001	0.002	1.68	1.38
Osteoarthritis of hip	345/54 885 (0.6)	250/55 176 (0.5)	0.002 (0.001, 0.003)	+0.2	−324	−23.85	1.26 (1.07, 1.48)	0.006	0.015	1.82	1.33
Gout	154/55 403 (0.3)	112/55 604 (0.2)	0.001 (0.000, 0.001)	+0.1	N/A	N/A	1.25 (0.98, 1.59)	0.074	0.124	N/A	N/A
NCOs	1103/49 044 (2.2)	966/49 919 (1.9)	0.003 (0.001, 0.005)	+0.3	N/A	N/A	1.05 (0.97, 1.15)	0.236	N/A	N/A	N/A

*Note*: Data are presented as events/total patients (proportion). Absolute risk differences were derived from Kaplan–Meier cumulative incidence at 720 days; NNT = 1/|ARD|, rounded upward, reported only for significant results. Hazard ratios with 95% confidence intervals were estimated using Cox proportional hazards models after 1:1 propensity score matching (215 covariates, calliper 0.2 SD). *p* values were adjusted using the Benjamini–Hochberg method. *E* values quantify the minimum strength of unmeasured confounding needed to explain away the observed association; reported only for significant results. Raw *p* values were derived from Cox proportional hazards models. Adjusted *p* values were calculated using the Benjamini‐Hochberg procedure to control the false discovery rate at 5% across all primary and secondary outcome comparisons within each cohort. E‐values represent the minimum confounder association strength needed to explain the observed result.

Abbreviations: ARD, absolute risk difference; BH, Benjamini–Hochberg; CI, confidence interval; HR, hazard ratio; MOF, major osteoporotic fracture; N/A, not applicable; NCO, negative control outcome; NNT, number needed to treat; RRR, relative risk reduction.

### 3.3 Comparative Associations of Semaglutide Initiation With Skeletal Outcomes Across Cohorts

Secondary skeletal outcomes are summarised in Table 1 and Figure 2. In the T2D cohort, semaglutide was not associated with osteoporosis risk versus any individual comparator, though the comparison with empagliflozin showed a trend (HR 0.90, 95% CI: 0.81–1.01; *p* = 0.071). In the non‐T2D cohort, osteoporosis associations were similarly non‐significant across all comparisons. Among exploratory outcomes, semaglutide was associated with modestly higher knee osteoarthritis hazards in the T2D cohort versus empagliflozin (HR 1.14, 95% CI: 1.06–1.23; *p* < 0.001) and sitagliptin (HR 1.16, 95% CI: 1.04–1.29; *p* = 0.006), and in the non‐T2D cohort versus usual care (HR 1.20, 95% CI: 1.08–1.32; *p* < 0.001; Table 2). Hip osteoarthritis was higher versus usual care in the non‐T2D cohort (HR 1.26, 95% CI: 1.07–1.48; *p* = 0.006) but not in other comparisons. Gout showed no significant associations across either cohort.

### 3.4 Per‐Protocol Analysis

In the PP analysis, the associations between semaglutide and skeletal outcomes were consistent with the ITT results across all persistence thresholds (e.g., semaglutide vs. empagliflozin for MOF: HR 0.59, 95% CI: 0.49–0.71, *p* < 0.001 at day 75; Table S11). Semaglutide consistently showed higher persistence than their respective comparators in both cohorts (Table S12).

### 3.5 Subgroup Analysis

Subgroup analyses are detailed in Tables S13–20. In the T2D cohort, semaglutide showed a stronger association with reduced osteoporosis risk among patients with HbA1c < 7% (HR 0.46, 95% CI 0.24–0.85; *p* for interaction = 0.03) compared with empagliflozin. Most other subgroup comparisons lacked statistical significance.

### 3.6 Sensitivity Analysis

In the T2D cohort, site‐specific fracture HRs for semaglutide versus empagliflozin ranged from 0.52 (hip) to 0.77 (humerus), all in the same direction (Tables S21 and S22). In the surgical code–confirmed hip fracture analysis (Figure S2), semaglutide was associated with a lower hazard versus empagliflozin (HR 0.45, 95% CI 0.30–0.67) and glipizide (HR 0.59, 0.35–1.00); comparisons versus sitagliptin and usual care did not reach significance, consistent with smaller event counts in this more restrictive definition. When MOF and all‐cause mortality were combined (Figure S3), the HR was 0.38 (0.36–0.41) versus 0.69 (0.61–0.77) for MOF alone; this gap was largely attributable to the mortality component. In the GLP‐1 RA class‐level sensitivity analysis, the associations with MOF were consistent with the individual semaglutide results, with lower hazards observed versus empagliflozin (HR 0.74, 95% CI: 0.66–0.83, *p* < 0.001), glipizide and usual care in the obesity with T2D cohort (Figure S4). Semaglutide was also associated with a lower hazard of MOF versus the pooled active comparator in the obesity with T2D cohort (HR 0.72, 95% CI: 0.65–0.80, *p* < 0.001), consistent with the individual comparisons (Figure S5). Results from the global network and three‐month landmark sensitivity analyses were consistent with the main findings (Figures S6 and S7).

### 3.7 Bone‐Active Medication Uses and Healthcare Utilisation; Longitudinal Analysis of BMI and HbA1c

Exploratory analyses of bone‐active medication patterns, healthcare utilisation and longitudinal BMI and HbA1c trajectories are reported in Figures S8–S10.

## 4 Discussion

In this long‐term propensity score–matched cohort of people with obesity with and without T2D, we observed several clinically relevant associations. Semaglutide initiation was associated with lower hazard of MOF, our primary endpoint. Osteoporosis and gout findings were generally non‐significant, whereas exploratory osteoarthritis findings were inconsistent across comparators.

Within T2D, semaglutide was associated with lower MOF hazards across all four comparators, and the direction was consistent regardless of the reference drug. Among those without T2D, a lower MOF hazard appeared chiefly versus usual care. Overall patterns seemed more evident in metabolically dysregulated groups. These results accord with preclinical literature on GLP‐1–related bone biology, and the composite MOF endpoint focuses on clinically consequential fracture sites [22, 23].

A meta‐analysis of 17 studies reported that GLP‐1 RAs modestly reduce serum uric acid, although a network meta‐analysis of 22 RCTs found that only SGLT2 inhibitors, not GLP‐1 RAs, significantly reduced gout incidence [24, 25]. One interpretation is that the association tracks with metabolic changes observed in GLP‐1RA trials; however, residual confounding and differential ascertainment (e.g., diagnostic work‐up) remain plausible [26, 27].

Semaglutide was associated with modestly higher knee and hip OA hazards in both cohorts, but the pattern was inconsistent across comparators (e.g., non‐T2D: higher vs. usual care, but not vs. phentermine‐topiramate). In the PP analysis, OA associations were attenuated and non‐significant in the non‐T2D cohort. Detection bias and channelling bias are more likely explanations than a direct pharmacological effect, as semaglutide users have more frequent clinic visits and may be preferentially prescribed the drug for existing joint complaints. These OA findings are hypothesis‐generating.

These results extend prior literature on GLP‐1 RAs and bone health [28, 29, 30, 31]. Preclinical studies identified GLP‐1 receptors on osteoblasts and osteoclasts [32, 33, 34], and GLP‐1 receptor knockout mice show cortical osteopenia and increased bone resorption [35]. Prior clinical data were limited to 1–2 years of follow‐up; our analysis provides longer‐term data.

T2D is associated with higher bone mineral density but increased fracture risk, attributed to impaired bone quality [36]. Chronic hyperglycemia promotes advanced glycation end product (AGE) accumulation in the collagen matrix, increasing brittleness and is linked to low bone turnover and cortical porosity [36]. GLP‐1 RAs may influence skeletal health indirectly through improved glycemic control, which could mitigate hyperglycemia‐related AGE accumulation and deterioration in bone material properties [37]. In the T2D cohort, semaglutide was associated with lower MOF hazards versus empagliflozin and glipizide, with a similar directional estimate versus sitagliptin. This overall pattern is broadly consistent with these preclinical data. The hip fracture estimate was lowest among fracture sites, which may reflect cortical bone, where T2D‐related porosity is greatest [36]. GLP‐1 RAs at obesity doses produce about 15% weight loss, which may decrease BMD through reduced mechanical loading. In the T2D cohort, lower fracture hazards with semaglutide occurred despite expected weight‐loss‐related BMD changes, suggesting that possible bone quality improvements or reduced fall risk may offset the mechanical unloading.

Some associations may reflect chance despite FDR correction. The most consistent MOF findings were versus empagliflozin, glipizide and usual care, remaining significant after FDR correction and supported by per‐protocol and site‐specific fracture analyses. The semaglutide–sitagliptin estimate was directionally similar but did not remain significant after correction and should be interpreted cautiously. Validated hip fracture showed the same pattern. Associations observed in a single comparison or with marginal significance should be interpreted with greater caution.

This study has several limitations. First, key fracture determinants: frailty, fall risk, physical activity, menopausal status, vitamin D, bone turnover markers and baseline BMD were not directly measurable in TriNetX. We matched on proxy variables (post‐matching: fall history 1.3% vs. 1.3%, DXA utilisation 3.6% vs. 3.8% in the semaglutide–empagliflozin comparison), but residual confounding remains. Healthier semaglutide users would bias results away from the null; preferential prescribing to sicker patients would bias in the opposite direction. For the semaglutide–empagliflozin MOF comparison, an unmeasured confounder would need a 2.28‐fold association with both treatment and outcome to explain the result. These findings should be viewed as hypothesis‐generating. Second, EHR data may under‐capture mild osteoarthritis, and residual confounding from symptom‐driven semaglutide prescribing for painful OA cannot be excluded. Third, dosage variations, treatment adherence and lifestyle changes could not be fully accounted for; estimates for low‐frequency outcomes are preliminary. Fourth, concomitant metformin/pioglitazone use was assessed only at baseline without modelling changes over time. Fifth, different comparator classes in the T2D and non‐T2D strata reflect prescribing practice differences, and residual channelling bias may persist. Results should be interpreted within each drug comparison. Semaglutide dosing patterns also differ by indication, with obesity regimens reaching higher maintenance doses than T2D regimens.

Outcome ascertainment relied on ICD‐10 codes; non‐differential misclassification would generally attenuate hazard ratios toward the null. Consistent lower fracture hazards across four anatomically distinct sites and the strongest association at the validated hip fracture endpoint [21] argue against coding artefact. Residual detection bias for osteoporosis and under‐reporting of vertebral fractures cannot be excluded. Chart‐level validation was not feasible within TriNetX. We acknowledge that MOF components differ in mechanism; hip fractures typically involve falls, whereas vertebral fractures may occur spontaneously, but individual site analyses showed consistent directions with the composite finding. Osteoporosis, unlike MOF, is a radiological diagnosis susceptible to detection bias from differential healthcare access; although propensity score matching included healthcare utilisation, visit frequency and prior osteoporosis screening, residual ascertainment differences cannot be excluded.

For people with T2D and elevated fracture risk, the lower MOF hazards observed with semaglutide in some comparisons warrant further study in trials capturing dose, adherence and bone imaging data.

## 5 Conclusion

In this study, initiation of semaglutide was associated with lower hazards for selected skeletal outcomes in some contrasts, with heterogeneity by diabetes status and comparator class. Given the observational design and potential residual confounding, these findings should be interpreted as comparative associations and considered hypothesis‐generating for future prospective studies.

## Author Contributions

**Yu‐Nan Huang:** conceptualisation, methodology, formal analysis, investigation, data curation, writing – original draft. **Min‐Yu Tsou:** conceptualisation, methodology, data curation. **Pin‐Hung Li:** data curation, investigation. **Jo‐Ching Chen:** validation, formal analysis. **Shao‐Chia Chen:** validation, formal analysis. **Hao‐I Hsieh:** methodology, validation. **Gideon Meyerowitz‐Katz:** methodology, validation, writing – review and editing. **Yen‐Liang Liu:** supervision, project administration, writing – review and editing. **Pen‐Hua Su:** supervision, project administration, resources, funding acquisition, writing – review and editing.

## Funding

Financial support for this investigation was provided by Taiwan's National Science and Technology Council (NSTC 113‐2124‐M‐039‐002, NSTC 114‐2124‐M‐039‐001, NSTC 113‐2314‐B‐040‐026‐MY2 and NSTC 114‐2622‐B‐040‐001) with supplementary funding from Chung Shan Medical University Hospital (CSH‐2026‐A‐009, CSH‐2026‐C‐030 and CSH‐2026‐F‐003); National Health Research Institutes (NHRI‐EX114‐11206EC and NHRI‐EX115‐11206EC). The study funder was not involved in the design of the study; the collection, analysis and interpretation of data; writing the report and did not impose any restrictions regarding the publication of the report.

## Ethics Statement

The Institutional Review Board at Chung Shan Medical University Hospital provided ethical approval for this research (CS2‐24004 and CS2‐24100).

## Consent

Individual consent requirements were waived as TriNetX provides only de‐identified aggregated data.

## Conflicts of Interest

The authors declare no conflicts of interest.

## Supporting information


**Table S1:** Protocol components of the target trial and their emulation in the TriNetX database analysis of semaglutide versus conventional therapies for skeletal outcomes in people with obesity.
**Table S2:** Proportions of missing data for key continuous covariates before propensity score matching.
**Table S3:** Baseline characteristics before and after propensity score matching: Semaglutide versus empagliflozin in obesity with type 2 diabetes.
**Table S4:** Baseline characteristics before and after propensity score matching: Semaglutide versus sitagliptin in obesity with type 2 diabetes.
**Table S5:** Baseline characteristics before and after propensity score matching: Semaglutide versus glipizide in obesity with type 2 diabetes.
**Table S6:** Baseline characteristics before and after propensity score matching: Semaglutide versus usual care in obesity with type 2 diabetes.
**Table S7:** Baseline characteristics before and after propensity score matching: Semaglutide versus naltrexone–bupropion in obesity without type 2 diabetes.
**Table S8:** Baseline characteristics before and after propensity score matching: Semaglutide versus phentermine in obesity without type 2 diabetes.
**Table S9:** Baseline characteristics before and after propensity score matching: Semaglutide versus phentermine–topiramate in obesity without type 2 diabetes.
**Table S10:** Baseline characteristics before and after propensity score matching: Semaglutide versus usual care in obesity without type 2 diabetes.
**Table S11:** Per‐protocol hazard ratios for skeletal outcomes across cumulative 75‐day refill intervals.
**Table S12:** Treatment persistence rates across cumulative 75‐day refill intervals by treatment group.
**Table S13:** Subgroup analysis of osteoporosis incidence during three‐year follow‐up: Semaglutide versus comparators in obesity with type 2 diabetes.
**Table S14:** Subgroup analysis of knee osteoarthritis incidence during three‐year follow‐up: Semaglutide versus comparators in obesity with type 2 diabetes.
**Table S15:** Subgroup analysis of hip osteoarthritis incidence during three‐year follow‐up: Semaglutide versus comparators in obesity with type 2 diabetes.
**Table S16:** Subgroup analysis of gout incidence during three‐year follow‐up: Semaglutide versus comparators in obesity with type 2 diabetes.
**Table S17:** Subgroup analysis of osteoporosis incidence during two‐year follow‐up: Semaglutide versus comparators in obesity without type 2 diabetes.
**Table S18:** Subgroup analysis of knee osteoarthritis incidence during two‐year follow‐up: Semaglutide versus comparators in obesity without type 2 diabetes.
**Table S19:** Subgroup analysis of hip osteoarthritis incidence during two‐year follow‐up: Semaglutide versus comparators in obesity without type 2 diabetes.
**Table S20:** Subgroup analysis of gout incidence during two‐year follow‐up: Semaglutide versus comparators in obesity without type 2 diabetes.
**Table S21:** Individual fracture components of major osteoporotic fracture in obesity with type 2 diabetes.
**Table S22:** Individual fracture components of major osteoporotic fracture in obesity without type 2 diabetes.
**Table S23:** Three‐year hazard ratios for skeletal outcomes associated with tirzepatide versus comparators in people with obesity and type 2 diabetes.
**Table S24:** Two‐year hazard ratios for skeletal outcomes associated with tirzepatide versus comparators in people with obesity without type 2 diabetes.
**Figure S1:** Directed acyclic graph (DAG) of causal pathways between GLP 1 receptor agonist treatment and skeletal health outcomes in obesity. This directed acyclic graph depicts hypothesised causal relationships between GLP‐1 receptor agonist medications (specifically semaglutide versus comparators) and skeletal outcomes in our target populations. Colour‐coding differentiates between baseline factors (blue), exposure (yellow), mediators (red) and skeletal outcomes (purple). Baseline characteristics (demographics, BMI, HbA1c) influence comorbidity patterns (T2D, hypertension, CVD) and laboratory measurements (eGFR, lipids, proteinuria), which collectively affect treatment selection decisions. GLP‐1 RA treatment influences skeletal outcomes through two primary mediating pathways: weight change (BMI reduction) and glycemic control (HbA1c reduction). The dashed line represents unmeasured factors that may influence both baseline characteristics and skeletal outcomes. Concurrent medications (metformin, insulin, ACEi/ARBs) are positioned as intermediary factors influenced by treatment selection decisions but also directly affecting the principal exposure. This framework guided our propensity score matching approach to minimise confounding and informed our subgroup analyses examining effect modification by comorbidities and laboratory parameters.
**Figure S2:** Surgery code–confirmed hip fracture analysis. Hip fracture was defined by ICD‐10 diagnosis codes (S72.0–S72.2) combined with inpatient surgical procedure codes (CPT 27235, 27 244, 27 245; ICD‐10‐PCS 0QS6, 0QS7), a validated hard endpoint with a reported positive predictive value exceeding 95% in administrative databases. CI, confidence interval; HR, hazard ratio; T2D, type 2 diabetes.
**Figure S3:** Forest plot for the composite endpoint of major osteoporotic fracture plus all‐cause mortality. This analysis addresses the potential influence of differential mortality as a competing event by combining MOF and all‐cause mortality into a single composite endpoint. Comparisons are shown for both the obesity with T2D cohort (3 year follow‐up) and the obesity without T2D cohort (2‐year follow‐up). HR, hazard ratio; CI, confidence interval.
**Figure S4:** GLP‐1 receptor agonist class‐level sensitivity analysis: Forest plot of skeletal outcomes. In this sensitivity analysis, the exposure was defined as initiation of any GLP‐1 receptor agonist (semaglutide, liraglutide, dulaglutide or exenatide) rather than semaglutide alone. All other design elements (matching, covariates, outcomes) were identical to the primary analysis. CI, confidence interval; HR, hazard ratio; MOF, major osteoporotic fracture; T2D, type 2 diabetes.
**Figure S5:** Semaglutide versus pooled active comparators: Forest plot of skeletal outcomes. The pooled active comparator group combined all individual active comparators within each cohort into a single reference group (T2D: empagliflozin + sitagliptin + glipizide; non‐T2D: naltrexone–bupropion + phentermine + phentermine–topiramate). CI, confidence interval; HR, hazard ratio; MOF, major osteoporotic fracture; T2D, type 2 diabetes.
**Figure S6:** Global network sensitivity analysis. This sensitivity analysis replicated the primary analysis using the TriNetX Global Collaborative Network (approximately 170 million patients across 80+ healthcare organisations in 30+ countries) to assess generalisability beyond US‐based data. All other design elements (matching, covariates, outcomes, follow‐up) were identical to the primary analysis. CI, confidence interval; HR, hazard ratio; MOF, major osteoporotic fracture; T2D, type 2 diabetes.
**Figure S7:** Three‐month landmark analysis for skeletal outcomes excluding early events. In this landmark analysis, patients who experienced an outcome event or were censored within the first 90 days after the index date were excluded. Follow‐up was counted from day 91 onward to reduce the influence of prevalent or misclassified events captured shortly after treatment initiation. CI, confidence interval; HR, hazard ratio; MOF, major osteoporotic fracture; T2D, type 2 diabetes.
**Figure S8:** Bone‐Active medication use and healthcare utilisation across treatment comparisons. Bone‐active medications include alendronate, zoledronic acid and denosumab. Healthcare utilisation outcomes include outpatient, emergency department and inpatient encounters. HRs were estimated using Cox proportional hazards models after propensity score matching. CI, confidence interval; HR, hazard ratio; T2D, type 2 diabetes.
**Figure S9:** Longitudinal changes in body mass index among semaglutide users versus comparator groups across metabolic phenotypes. This figure illustrates the five‐year trajectory of body mass index (BMI) changes (expressed as percentage change from baseline) across treatment groups, stratified by diabetes status. The left panels demonstrate changes in obesity with type 2 diabetes receiving semaglutide versus conventional glucose‐lowering medications (empagliflozin, sitagliptin, glipizide and usual care). The right panels display corresponding changes in obesity without T2D receiving semaglutide versus traditional anti‐obesity treatments (naltrexone‐bupropion, phentermine, phentermine‐topiramate and non‐users). *Y*‐axis values represent percentage changes from baseline, with baseline normalised to 100%.
**Figure S10:** Differential glycemic control trajectories with semaglutide versus comparator therapies in obesity with and without type 2 diabetes. This figure presents longitudinal changes in glycated haemoglobin (HbA1c) over a five‐year period, expressed as percentage change from baseline. The left panels show obese individuals with type 2 diabetes receiving semaglutide versus conventional antidiabetic medications (empagliflozin, sitagliptin, glipizide and usual care). The right panels display non‐diabetic obese individuals receiving semaglutide versus alternative anti‐obesity treatments (naltrexone‐bupropion, phentermine, phentermine‐topiramate and non‐users). *Y*‐axis values represent percentage changes from baseline, with baseline normalised to 100%.

**Data S1:** dom70786‐sup‐0002‐Supinfo2.docx.

## Data Availability

We analyzed pooled, de‐identified data from the TriNetX platform for this research. Due to patient privacy laws and proprietary elements owned by TriNetX, raw individual‐level data cannot be shared publicly. Researchers interested in accessing these datasets must submit a formal request to our institutional review board and must have executed the standard license agreement with TriNetX, which includes restrictive covenants governing the use of the data.
