# Supplementary material for: Associations of Semaglutide With Skeletal Outcomes in People With Obesity, With and Without Type 2 Diabetes: A Target Trial Emulation
Source: Diabetes Obes Metab. 2026 Apr 20;28(7):5834–47. doi: 10.1111/dom.70786 (PMC13243989; doi:10.1111/dom.70786)
Supplement: Supplementary file 1 — Table S1: Protocol components of the target trial and their emulation in the TriNetX database analysis of semaglutide versus conventional therapies for skeletal outcomes in people with obesity. Table S2: Proportions of missing data for key continuous covariates before propensity score matching. Table S3: Baseline characteristics before and after propensity score matching: Semaglutide versus empagliflozin in obesity with type 2 diabetes. Table S4: Baseline characteristics before and after propensity score matching: Semaglutide versus sitagliptin in obesity with type 2 diabetes. Table S5: Baseline characteristics before and after propensity score matching: Semaglutide versus glipizide in obesity with type 2 diabetes. Table S6: Baseline characteristics before and after propensity score matching: Semaglutide versus usual care in obesity with type 2 diabetes. Table S7: Baseline characteristics before and after propensity score matching: Semaglutide versus naltrexone–bupropion in obesity without type 2 diabetes. Table S8: Baseline characteristics before and after propensity score matching: Semaglutide versus phentermine in obesity without type 2 diabetes. Table S9: Baseline characteristics before and after propensity score matching: Semaglutide versus phentermine–topiramate in obesity without type 2 diabetes. Table S10: Baseline characteristics before and after propensity score matching: Semaglutide versus usual care in obesity without type 2 diabetes. Table S11: Per‐protocol hazard ratios for skeletal outcomes across cumulative 75‐day refill intervals. Table S12: Treatment persistence rates across cumulative 75‐day refill intervals by treatment group. Table S13: Subgroup analysis of osteoporosis incidence during three‐year follow‐up: Semaglutide versus comparators in obesity with type 2 diabetes. Table S14: Subgroup analysis of knee osteoarthritis incidence during three‐year follow‐up: Semaglutide versus comparators in obesity with type 2 diabetes. Table S15: Subgroup an [file DOM-28-5834-s001.pdf]

Supplementary Material

Yu-Nan Huang[1,2], Min-Yu Tsou[3], Pin-Hung Li[1,2], Jo-Ching Chen[1,2], Shao-Chia Chen[1,4], Hao-I Hsieh[5,6], Gideon Meyerowitz-Katz[7], Yen-Liang Liu[3,8,*], Pen-Hua Su[1,2,*]. Associations of Semaglutide with Skeletal Outcomes in People with Obesity, with and without Type 2 Diabetes: A Target Trial Emulation

[1]Division of Genetics and Endocrinology, Department of Pediatrics, Chung Shan Medical University Hospital, Taichung, Taiwan; [2]School of Medicine, Chung Shan Medical University; Taichung, Taiwan; [3]Cancer Biology and Precision Therapeutics Center, China Medical University, Taichung, Taiwan; [4]Institute of Medicine, Chung Shan Medical University, Taichung, Taiwan; [5]School of Medicine, College of Medicine, National Taiwan University, Taipei, Taiwan; [6]Department of Education, National Taiwan University Hospital, Taipei, Taiwan; [7]School of Health and Society, University of Wollongong, Wollongong, Australia; [8]Master Program for Biomedical Engineering, China Medical University, Taichung, Taiwan.

Correspondence to:

Pen-Hua Su, MD, PhD
Department Head and Professor
Division of Genetics and Endocrinology, Department of Pediatrics, Chung Shan Medical University Hospital.
No. 110, Sec. 1, Jianguo N. Rd., South Dist., Taichung City 402306, Taiwan.
Tel: +886 4739595 (Ext. 21707)
E-mail: ninaphsu@gmail.com

Yen-Liang Liu, PhD
Cancer Biology and Precision Therapeutics Center, China Medical University
No. 2, Yude Rd., North Dist., Taichung City 404327, Taiwan
Tel: +886-912378780
email: yl.allen.liu@gmail.com

(A) Supplementary material and methods
Data Source and Study Population
Data Infrastructure and Quality Assurance
Addressing Bias
Statistical Methodology and Confounding Control
Security Measures and Data Accessibility
Study Limitations

(B) Database, cohorts, covariates, and outcome definition

(C) Supplementary results

(D) Supplementary tables
Supplementary Table 1. Protocol components of the target trial and their emulation in the TriNetX database analysis of semaglutide versus conventional therapies for skeletal outcomes in people with obesity.
Supplementary Table 2. Proportions of Missing Data for Key Continuous Covariates Before Propensity Score Matching.
Supplementary Table 3. Baseline Characteristics Before and After Propensity Score Matching: Semaglutide Versus Empagliflozin in Obesity With Type 2 Diabetes.
Supplementary Table 4. Baseline Characteristics Before and After Propensity Score Matching: Semaglutide Versus Sitagliptin in Obesity With Type 2 Diabetes.
Supplementary Table 5. Baseline Characteristics Before and After Propensity Score Matching: Semaglutide Versus Glipizide in Obesity With Type 2 Diabetes.
Supplementary Table 6. Baseline Characteristics Before and After Propensity Score Matching: Semaglutide Versus Usual Care in Obesity With Type 2 Diabetes.
Supplementary Table 7. Baseline Characteristics Before and After Propensity Score Matching: Semaglutide Versus Naltrexone–Bupropion in Obesity Without Type 2 Diabetes.
Supplementary Table 8. Baseline Characteristics Before and After Propensity Score Matching: Semaglutide Versus Phentermine in Obesity Without Type 2 Diabetes.
Supplementary Table 9. Baseline Characteristics Before and After Propensity Score Matching: Semaglutide Versus Phentermine–Topiramate in Obesity Without Type 2 Diabetes.
Supplementary Table 10. Baseline Characteristics Before and After Propensity Score Matching: Semaglutide Versus Usual Care in Obesity Without Type 2 Diabetes.
Supplementary Table 11. Per-Protocol Hazard Ratios for Skeletal Outcomes Across Cumulative 75-Day Refill Intervals.

Supplementary Table 12. Treatment Persistence Rates Across Cumulative 75-Day Refill Intervals by Treatment Group.
Supplementary Table 13. Subgroup Analysis of Osteoporosis Incidence During Three-Year Follow-Up: Semaglutide Versus Comparators in Obesity With Type 2 Diabetes.
Supplementary Table 14. Subgroup Analysis of Knee Osteoarthritis Incidence During Three-Year Follow-Up: Semaglutide Versus Comparators in Obesity With Type 2 Diabetes.
Supplementary Table 15. Subgroup Analysis of Hip Osteoarthritis Incidence During Three-Year Follow-Up: Semaglutide Versus Comparators in Obesity With Type 2 Diabetes.
Supplementary Table 16. Subgroup Analysis of Gout Incidence During Three-Year Follow-Up: Semaglutide Versus Comparators in Obesity With Type 2 Diabetes.
Supplementary Table 17. Subgroup Analysis of Osteoporosis Incidence During Two-Year Follow-Up: Semaglutide Versus Comparators in Obesity Without Type 2 Diabetes.
Supplementary Table 18. Subgroup Analysis of Knee Osteoarthritis Incidence During Two-Year Follow-Up: Semaglutide Versus Comparators in Obesity Without Type 2 Diabetes.
Supplementary Table 19. Subgroup Analysis of Hip Osteoarthritis Incidence During Two-Year Follow-Up: Semaglutide Versus Comparators in Obesity Without Type 2 Diabetes.
Supplementary Table 20. Subgroup Analysis of Gout Incidence During Two-Year Follow-Up: Semaglutide Versus Comparators in Obesity Without Type 2 Diabetes.
Supplementary Table 21. Individual Fracture Components of Major Osteoporotic Fracture in Obesity with Type 2 Diabetes.
Supplementary Table 22. Individual Fracture Components of Major Osteoporotic Fracture in Obesity without Type 2 Diabetes.
Supplementary Table 23. Three-Year Hazard Ratios for Skeletal Outcomes Associated With Tirzepatide Versus Comparators in People With Obesity and Type 2 Diabetes.
Supplementary Table 24. Two-Year Hazard Ratios for Skeletal Outcomes Associated With Tirzepatide Versus Comparators in People With Obesity Without Type 2 Diabetes.

(E) Supplementary figures

Supplementary Figure 1. Directed Acyclic Graph (DAG) of Causal Pathways Between GLP-1 Receptor Agonist Treatment and Skeletal Health Outcomes in Obesity.
Supplementary Figure 2. Surgery Code–Confirmed Hip Fracture Analysis.
Supplementary Figure 3. Forest plot for the Composite Endpoint of Major Osteoporotic Fracture Plus All-Cause Mortality.
Supplementary Figure 4. GLP-1 Receptor Agonist Class-Level Sensitivity Analysis: Forest Plot of Skeletal Outcomes.
Supplementary Figure 5. Semaglutide Versus Pooled Active Comparators: Forest Plot of Skeletal Outcomes.

Supplementary Figure 6. Global Network Sensitivity Analysis.
Supplementary Figure 7. Three-Month Landmark Analysis for Skeletal Outcomes Excluding Early Events.
Supplementary Figure 8. Bone-Active Medication Use and Healthcare Utilization Across Treatment Comparisons.
Supplementary Figure 9. Longitudinal Changes in Body Mass Index Among Semaglutide Users Versus Comparator Groups Across Metabolic Phenotypes.
Supplementary Figure 10. Differential Glycemic Control Trajectories with Semaglutide Versus Comparator Therapies in Obesity With and Without Type 2 diabetes.

(F) References

Data Source and Study Population

Our investigation utilized the TriNetX US Collaborative Network, accessing de-identified medical records from more than 120 million individuals across 65 healthcare organizations throughout the United States. We analyzed comprehensive clinical data collected between January 2018 and December 2025. encompassing demographics, ICD-10-CM diagnostic codes, medication prescriptions, procedures, and laboratory values. Chung Shan Medical University Hospital's Institutional Review Board approved our research (approval numbers CS2-24004 and CS2-24100), waiving informed consent requirements given the de-identified nature of aggregate data. We strictly followed STROBE guidelines to ensure transparent and reproducible reporting of our observational findings.

Data Infrastructure and Quality Assurance

The TriNetX platform employs a hub-and-spoke model where healthcare organizations contribute de-identified patient data to a secure, HIPAA-compliant cloud infrastructure. The system utilizes advanced extract-transform-load processes to harmonize data from various sources, including i2b2 research repositories and other common data models. To maintain data integrity across diverse sources, we implemented quality control measures based on fundamental principles of cleanliness, consistency, correctness, and completeness. These principles guided our systematic identification of outliers, standardization of data formats, validation against established clinical parameters, and documentation of missing data patterns.

Addressing Bias

Various bias sources plague pharmacoepidemiological investigations of obesity treatments, primarily immortal time bias, selection bias, and confounding factors[1, 2]. Our investigation into GLP-1 receptor agonists and conventional anti-obesity medications employed multiple methodological approaches to minimize these potential distortions[3].
When studying chronic obesity treatments, immortal time bias emerged as a critical consideration. We acknowledged that patients must survive sufficiently long to receive and continue their prescribed therapies, potentially creating artificial advantages for treatment cohorts. To mitigate immortal time bias, we aligned time zero at treatment initiation (T_0) and applied the same eligibility and baseline covariate assessment windows across comparator arms. The primary analysis used ITT-type follow-up from T_0 under real-world treatment changes[4, 5]. This methodology helped ensure that our exposure classifications accurately mirrored actual therapeutic patterns. Additionally, we incorporated time-dependent exposure definitions based on prescription records.

The rapidly shifting landscape of obesity treatment guidelines and differential access to newer medications presented selection bias challenges. We tackled this through the implementation of comprehensive eligibility standards applied consistently across all treatment groups. The TriNetX platform's requisite regular follow-up documentation helped reduce differential attrition. Through meticulous propensity matching, we achieved well-balanced baseline characteristics across cohorts, with standardized mean differences remaining under 0.1 for matched variables[6].
Confounding by indication warranted particular attention since newer GLP-1 receptor agonists might be selectively prescribed to specific patient populations. To address this concern, we implemented an active comparator design that evaluated GLP-1 receptor agonists against established anti-obesity or glucose-lowering medications. Our propensity score matching incorporated numerous covariates spanning demographics, lifestyle behaviors, comorbid conditions, concurrent pharmacotherapies, and laboratory values. To evaluate our findings' resilience against unmeasured confounding, we conducted negative control analyses with pre-specified endpoints and calculated E-values for statistically significant associations[7, 8].
To bolster the validity of our conclusions, we conducted several sensitivity analyses. These included evaluating outcomes across varying follow-up durations. We also performed subgroup analyses examining the effects of all available GLP-1 RA brands (including liraglutide, dulaglutide, exenatide, and others) to assess consistency of treatment effects the all-drug class. The remarkable consistency observed across these diverse analytical approaches lends credibility to our primary findings despite the inherent limitations of observational research methodologies.

Statistical Methodology and Confounding Control

Our statistical framework integrated multiple approaches to ensure robust analysis and control for potential confounding factors. The foundation of our methodology rested on propensity score matching, which we implemented through a nearest-neighbor (NN) matching approach using a 1:1 ratio with a caliper of 0.2 standard deviations. This technique balances covariates between study groups while maintaining adequate sample sizes. The matching procedure began with constructing a covariate matrix containing both binary indicators and continuous variables, with distinct binary columns created for categorical variables. We utilized logistic regression through the scikit-learn package (version 1.2.2, C=1000) to generate propensity scores, calculating each patient's probability of treatment group assignment based on baseline characteristics.
To ensure robust matching across our multi-center dataset, we first sorted records by healthcare organization identifiers and performed random shuffling with a fixed seed (seed=42), eliminating potential ordering bias while maintaining reproducibility. For each patient in the smaller cohort, we identified the closest unmatched control patient whose

propensity score fell within our predefined caliper. We evaluated matching success through standardized mean differences (SMDs), calculating absolute standardized mean differences using established formulas for both continuous and binary variables. Values below 0.1 indicated adequate balance between groups.
Our comprehensive statistical analysis incorporated three main components. First, we conducted a detailed Measure of Association Analysis to evaluate outcome prevalence across patient groups, incorporating numerous adjustment factors ranging from basic patient characteristics to complex health indicators and medication profiles. Second, we employed Kaplan-Meier survival analysis to track outcome probabilities across daily intervals, implementing appropriate censoring techniques for patient dropout. Patients were typically censored either at the day following their last recorded clinical fact or, for pre-window outcomes, at the analysis window's start. Third, we performed a number of Instances analysis, carefully defining instance criteria for both date-based and visit-based outcomes to ensure consistent measurement across all study participants.

Security Measures and Data Accessibility

TriNetX maintains stringent security protocols, operating on a HIPAA-compliant virtual private cloud hosted by Amazon Web Services. The platform meets industry-standard security certifications including FedRAMP and NIST 800-53, with all communications secured using Transport Layer Security (TLS) with 2048-bit encryption. For data access, qualified researchers may submit detailed research proposals to our institutional committee, typically receiving decisions within 14 business days. Interested researchers should contact Professor Pen-Hua Su (ninaphsu@gmail.com).

Study Limitations

Our study faced several important limitations inherent to real-world data analysis. While our nearest-neighbor matching approach offered advantages in terms of sample size retention and covariate balance, it may have introduced minor residual differences, particularly in complex or unbalanced covariate distributions. Additionally, the potential for missed events at non-participating healthcare organizations and variable completeness across different healthcare settings may impact our findings. Despite these limitations, our large sample size, diverse patient population, and comprehensive analytical approach provide robust insights into the comparative safety profiles of the studied medications.

Demographic, diagnostic, procedural, medication, visit, and laboratory codes used in the definition of the cohorts.

Category	Code	Description
**Tirzepatide group (with T2D)**		
**#1: At least 20 years old (must have the following).**		
Demographics	Age	Age (at least 20 years)
**#2: Individuals with obesity and type 2 diabetes treated with tirzepatide (#2.3 and #2.4 must be fulfilled after #2.1 and #2.2).<br>Date constraint: The terms in this group occurred between May 13, 2022, and Feb 28, 2026.**		
**#2.1: Individuals with obesity (must have any of the following).**		
Diagnosis	UMLS:ICD10CM:E66	Overweight and obesity
Laboratory	TNX:9083	BMI (at least 27.00 kg/m^2)
**#2.2: With type 2 diabetes (must have the following).**		
Diagnosis	UMLS:ICD10CM:E11	Type 2 diabetes mellitus
**#2.3: Receiving tirzepatide (must have the following).**		
Medication	NLM:RXNORM:2601723	tirzepatide
**#2.4: No use of the following medications (cannot have any of the following).**		
Medication	NLM:ATC:A10BK	Sodium-glucose co-transporter 2 (SGLT2) inhibitors
Medication	NLM:ATC:A10BJ	Glucagon-like peptide-1 (GLP-1) analogues
Medication	NLM:RXNORM:4821	glipizide
Medication	NLM:RXNORM:593411	sitagliptin
**#3: Without T1D, other diabetes, ESRD, HIV, transplant surgery or bariatric surgery (cannot have any of the following).**		
Diagnosis	UMLS:ICD10CM:E10	Type 1 diabetes mellitus
Diagnosis	UMLS:ICD10CM:E08	Diabetes mellitus due to underlying condition
Diagnosis	UMLS:ICD10CM:E09	Drug or chemical induced diabetes mellitus
Diagnosis	UMLS:ICD10CM:E13	Other specified diabetes mellitus
Diagnosis	UMLS:ICD10CM:N18.6	End stage renal disease
Diagnosis	UMLS:ICD10CM:B20	Human immunodeficiency virus [HIV] disease
Diagnosis	UMLS:ICD10CM:Z98.84	Bariatric surgery status
Procedure	UMLS:CPT:1007385	Bariatric Surgery Procedures
Diagnosis	UMLS:ICD10CM:Z94	Transplanted organ and tissue status
Procedure	UMLS:SNOMED:77465005	Transplantation
Diagnosis	UMLS:ICD10CM:T86	Complications of transplanted organs and tissue
**#4: Without prior exposure to the following medications (cannot have any of the following). #4 must be fulfilled within 6 months on or before #2.3**		
Medication	NLM:RXNORM:2601723	tirzepatide
Medication	NLM:ATC:A10BK	Sodium-glucose co-transporter 2 (SGLT2) inhibitors
Medication	NLM:ATC:A10BJ	Glucagon-like peptide-1 (GLP-1) analogues
Medication	NLM:RXNORM:4821	glipizide
Medication	NLM:RXNORM:593411	sitagliptin
**#5: No prior musculoskeletal events (cannot have any of the following). #5 must be fulfilled within 1 year before #2.3**		
Diagnosis	UMLS:ICD10CM:M80	Osteoporosis with current pathological fracture
Diagnosis	UMLS:ICD10CM:M81	Osteoporosis without current pathological fracture
Diagnosis	UMLS:ICD10CM:M17	Osteoarthritis of knee
Diagnosis	UMLS:ICD10CM:M16	Osteoarthritis of hip
Diagnosis	UMLS:ICD10CM:M10	Gout
Diagnosis	UMLS:ICD10CM:S72.0	Fracture of head and neck of femur

Diagnosis	UMLS:ICD10CM:S72.1	Pertrochanteric fracture
Diagnosis	UMLS:ICD10CM:S72.2	Subtrochanteric fracture of femur
Diagnosis	UMLS:ICD10CM:S22.0	Fracture of thoracic vertebra
Diagnosis	UMLS:ICD10CM:S32.0	Fracture of lumbar vertebra
Diagnosis	UMLS:ICD10CM:S52.5	Fracture of lower end of radius
Diagnosis	UMLS:ICD10CM:S52.6	Fracture of lower end of ulna
Diagnosis	UMLS:ICD10CM:S42.2	Fracture of upper end of humerus

**Semaglutide group (with T2D)**		
**#1: At least 20 years old (must have the following).**		
Demographics	Age	Age (at least 20 years)
**#2: Individuals with obesity and type 2 diabetes treated with semaglutide (#2.3 and #2.4 must be fulfilled after #2.1 and #2.2).<br>Date constraint: The terms in this group occurred between May 13, 2022, and Feb 28, 2026.**		
**#2.1: Individuals with obesity (must have any of the following).**		
Diagnosis	UMLS:ICD10CM:E66	Overweight and obesity
Laboratory	TNX:9083	BMI (at least 27.00 kg/m^2)
**#2.2: With type 2 diabetes (must have the following).**		
Diagnosis	UMLS:ICD10CM:E11	Type 2 diabetes mellitus
**#2.3: Receiving semaglutide (must have the following).**		
Medication	NLM:ATC:A10BJ	Glucagon-like peptide-1 (GLP-1) analogues (Brand: Ozempic or Rybelsus or Wegovy)
**#2.4: No use of the following medications (cannot have any of the following).**		
Medication	NLM:ATC:A10BK	Sodium-glucose co-transporter 2 (SGLT2) inhibitors
Medication	NLM:RXNORM:2601723	tirzepatide
Medication	NLM:RXNORM:4821	glipizide
Medication	NLM:RXNORM:593411	sitagliptin
**#3: Without T1D, other diabetes, ESRD, HIV, transplant surgery or bariatric surgery (cannot have any of the following).**		
Diagnosis	UMLS:ICD10CM:E10	Type 1 diabetes mellitus
Diagnosis	UMLS:ICD10CM:E08	Diabetes mellitus due to underlying condition
Diagnosis	UMLS:ICD10CM:E09	Drug or chemical induced diabetes mellitus
Diagnosis	UMLS:ICD10CM:E13	Other specified diabetes mellitus
Diagnosis	UMLS:ICD10CM:N18.6	End stage renal disease
Diagnosis	UMLS:ICD10CM:B20	Human immunodeficiency virus [HIV] disease
Diagnosis	UMLS:ICD10CM:Z98.84	Bariatric surgery status
Procedure	UMLS:CPT:1007385	Bariatric Surgery Procedures
Diagnosis	UMLS:ICD10CM:Z94	Transplanted organ and tissue status
Procedure	UMLS:SNOMED:77465005	Transplantation
Diagnosis	UMLS:ICD10CM:T86	Complications of transplanted organs and tissue
**#4: Without prior exposure to the following medications (cannot have any of the following). #4 must be fulfilled within 6 months on or before #2.3**		
Medication	NLM:RXNORM:2601723	tirzepatide
Medication	NLM:ATC:A10BK	Sodium-glucose co-transporter 2 (SGLT2) inhibitors
Medication	NLM:ATC:A10BJ	Glucagon-like peptide-1 (GLP-1) analogues
Medication	NLM:RXNORM:4821	glipizide
Medication	NLM:RXNORM:593411	sitagliptin
**#5: No prior musculoskeletal events (cannot have any of the following). #5 must be fulfilled within 1 year before #2.3**		
Diagnosis	UMLS:ICD10CM:M80	Osteoporosis with current pathological fracture
Diagnosis	UMLS:ICD10CM:M81	Osteoporosis without current pathological fracture

Diagnosis	UMLS:ICD10CM:M17	Osteoarthritis of knee
Diagnosis	UMLS:ICD10CM:M16	Osteoarthritis of hip
Diagnosis	UMLS:ICD10CM:M10	Gout
Diagnosis	UMLS:ICD10CM:S72.0	Fracture of head and neck of femur
Diagnosis	UMLS:ICD10CM:S72.1	Pertrochanteric fracture
Diagnosis	UMLS:ICD10CM:S72.2	Subtrochanteric fracture of femur
Diagnosis	UMLS:ICD10CM:S22.0	Fracture of thoracic vertebra
Diagnosis	UMLS:ICD10CM:S32.0	Fracture of lumbar vertebra
Diagnosis	UMLS:ICD10CM:S52.5	Fracture of lower end of radius
Diagnosis	UMLS:ICD10CM:S52.6	Fracture of lower end of ulna
Diagnosis	UMLS:ICD10CM:S42.2	Fracture of upper end of humerus

**Empagliflozin group (with T2D)**		
**#1: At least 20 years old (must have the following).**		
Demographics	Age	Age (at least 20 years)
**#2: Individuals with obesity and type 2 diabetes treated with empagliflozin (#2.3 and #2.4 must be fulfilled after #2.1 and #2.2).<br>Date constraint: The terms in this group occurred between May 13, 2022, and Feb 28, 2026.**		
**#2.1: Individuals with obesity (must have any of the following).**		
Diagnosis	UMLS:ICD10CM:E66	Overweight and obesity
Laboratory	TNX:9083	BMI (at least 27.00 kg/m^2)
**#2.2: With type 2 diabetes (must have the following).**		
Diagnosis	UMLS:ICD10CM:E11	Type 2 diabetes mellitus
**#2.3: Receiving empagliflozin (must have the following).**		
Medication	NLM:ATC:A10BK	Sodium-glucose co-transporter 2 (SGLT2) inhibitors (Brand:Jardiance)
**#2.4: No use of the following medications (cannot have any of the following).**		
Medication	NLM:RXNORM:2601723	tirzepatide
Medication	NLM:ATC:A10BJ	Glucagon-like peptide-1 (GLP-1) analogues
Medication	NLM:RXNORM:4821	glipizide
Medication	NLM:RXNORM:593411	sitagliptin
**#3: Without T1D, other diabetes, ESRD, HIV, transplant surgery or bariatric surgery (cannot have any of the following).**		
Diagnosis	UMLS:ICD10CM:E10	Type 1 diabetes mellitus
Diagnosis	UMLS:ICD10CM:E08	Diabetes mellitus due to underlying condition
Diagnosis	UMLS:ICD10CM:E09	Drug or chemical induced diabetes mellitus
Diagnosis	UMLS:ICD10CM:E13	Other specified diabetes mellitus
Diagnosis	UMLS:ICD10CM:N18.6	End stage renal disease
Diagnosis	UMLS:ICD10CM:B20	Human immunodeficiency virus [HIV] disease
Diagnosis	UMLS:ICD10CM:Z98.84	Bariatric surgery status
Procedure	UMLS:CPT:1007385	Bariatric Surgery Procedures
Diagnosis	UMLS:ICD10CM:Z94	Transplanted organ and tissue status
Procedure	UMLS:SNOMED:77465005	Transplantation
Diagnosis	UMLS:ICD10CM:T86	Complications of transplanted organs and tissue
**#4: Without prior exposure to the following medications (cannot have any of the following). #4 must be fulfilled within 6 months on or before #2.3**		
Medication	NLM:RXNORM:2601723	tirzepatide
Medication	NLM:ATC:A10BK	Sodium-glucose co-transporter 2 (SGLT2) inhibitors
Medication	NLM:ATC:A10BJ	Glucagon-like peptide-1 (GLP-1) analogues
Medication	NLM:RXNORM:4821	glipizide
Medication	NLM:RXNORM:593411	sitagliptin

#5: No prior musculoskeletal events (cannot have any of the following). #5 must be fulfilled within 1 year before #2.3		
Diagnosis	UMLS:ICD10CM:M80	Osteoporosis with current pathological fracture
Diagnosis	UMLS:ICD10CM:M81	Osteoporosis without current pathological fracture
Diagnosis	UMLS:ICD10CM:M17	Osteoarthritis of knee
Diagnosis	UMLS:ICD10CM:M16	Osteoarthritis of hip
Diagnosis	UMLS:ICD10CM:M10	Gout
Diagnosis	UMLS:ICD10CM:S72.0	Fracture of head and neck of femur
Diagnosis	UMLS:ICD10CM:S72.1	Pertrochanteric fracture
Diagnosis	UMLS:ICD10CM:S72.2	Subtrochanteric fracture of femur
Diagnosis	UMLS:ICD10CM:S22.0	Fracture of thoracic vertebra
Diagnosis	UMLS:ICD10CM:S32.0	Fracture of lumbar vertebra
Diagnosis	UMLS:ICD10CM:S52.5	Fracture of lower end of radius
Diagnosis	UMLS:ICD10CM:S52.6	Fracture of lower end of ulna
Diagnosis	UMLS:ICD10CM:S42.2	Fracture of upper end of humerus

Sitagliptin group (with T2D)		
**#1: At least 20 years old (must have the following).**		
Demographics	Age	Age (at least 20 years)
**#2: Individuals with obesity and type 2 diabetes treated with sitagliptin (#2.3 and #2.4 must be fulfilled after #2.1 and #2.2).<br>Date constraint: The terms in this group occurred between May 13, 2022, and Feb 28, 2026.**		
**#2.1: Individuals with obesity (must have any of the following).**		
Diagnosis	UMLS:ICD10CM:E66	Overweight and obesity
Laboratory	TNX:9083	BMI (at least 27.00 kg/m^2)
**#2.2: With type 2 diabetes (must have the following).**		
Diagnosis	UMLS:ICD10CM:E11	Type 2 diabetes mellitus
**#2.3: Receiving sitagliptin (must have the following).**		
Medication	NLM:RXNORM:593411	sitagliptin
**#2.4: No use of the following medications (cannot have any of the following).**		
Medication	NLM:ATC:A10BJ	Glucagon-like peptide-1 (GLP-1) analogues
Medication	NLM:ATC:A10BK	Sodium-glucose co-transporter 2 (SGLT2) inhibitors
Medication	NLM:RXNORM:2601723	tirzepatide
Medication	NLM:RXNORM:4821	glipizide
**#3: Without T1D, other diabetes, ESRD, HIV, transplant surgery or bariatric surgery (cannot have any of the following).**		
Diagnosis	UMLS:ICD10CM:E10	Type 1 diabetes mellitus
Diagnosis	UMLS:ICD10CM:E08	Diabetes mellitus due to underlying condition
Diagnosis	UMLS:ICD10CM:E09	Drug or chemical induced diabetes mellitus
Diagnosis	UMLS:ICD10CM:E13	Other specified diabetes mellitus
Diagnosis	UMLS:ICD10CM:N18.6	End stage renal disease
Diagnosis	UMLS:ICD10CM:B20	Human immunodeficiency virus [HIV] disease
Diagnosis	UMLS:ICD10CM:Z98.84	Bariatric surgery status
Procedure	UMLS:CPT:1007385	Bariatric Surgery Procedures
Diagnosis	UMLS:ICD10CM:Z94	Transplanted organ and tissue status
Procedure	UMLS:SNOMED:77465005	Transplantation
Diagnosis	UMLS:ICD10CM:T86	Complications of transplanted organs and tissue
**#4: Without prior exposure to the following medications (cannot have any of the following). #4 must be fulfilled within 6 months on or before #2.3**		
Medication	NLM:RXNORM:2601723	tirzepatide
Medication	NLM:ATC:A10BK	Sodium-glucose co-transporter 2 (SGLT2) inhibitors

Medication	NLM:ATC:A10BJ	Glucagon-like peptide-1 (GLP-1) analogues
Medication	NLM:RXNORM:4821	glipizide
Medication	NLM:RXNORM:593411	sitagliptin
**#5: No prior musculoskeletal events (cannot have any of the following). #5 must be fulfilled within 1 year before #2.3**		
Diagnosis	UMLS:ICD10CM:M80	Osteoporosis with current pathological fracture
Diagnosis	UMLS:ICD10CM:M81	Osteoporosis without current pathological fracture
Diagnosis	UMLS:ICD10CM:M17	Osteoarthritis of knee
Diagnosis	UMLS:ICD10CM:M16	Osteoarthritis of hip
Diagnosis	UMLS:ICD10CM:M10	Gout
Diagnosis	UMLS:ICD10CM:S72.0	Fracture of head and neck of femur
Diagnosis	UMLS:ICD10CM:S72.1	Pertrochanteric fracture
Diagnosis	UMLS:ICD10CM:S72.2	Subtrochanteric fracture of femur
Diagnosis	UMLS:ICD10CM:S22.0	Fracture of thoracic vertebra
Diagnosis	UMLS:ICD10CM:S32.0	Fracture of lumbar vertebra
Diagnosis	UMLS:ICD10CM:S52.5	Fracture of lower end of radius
Diagnosis	UMLS:ICD10CM:S52.6	Fracture of lower end of ulna
Diagnosis	UMLS:ICD10CM:S42.2	Fracture of upper end of humerus

**Glipizide group (with T2D)**		
**#1: At least 20 years old (must have the following).**		
Demographics	Age	Age (at least 20 years)
**#2: Individuals with obesity and type 2 diabetes treated with glipizide (#2.3 and #2.4 must be fulfilled after #2.1 and #2.2).<br>Date constraint: The terms in this group occurred between May 13, 2022, and Feb 28, 2026.**		
**#2.1: Individuals with obesity (must have any of the following).**		
Diagnosis	UMLS:ICD10CM:E66	Overweight and obesity
Laboratory	TNX:9083	BMI (at least 27.00 kg/m^2)
**#2.2: With type 2 diabetes (must have the following).**		
Diagnosis	UMLS:ICD10CM:E11	Type 2 diabetes mellitus
**#2.3: Receiving glipizide (must have the following).**		
Medication	NLM:RXNORM:4821	glipizide
**#2.4: No use of the following medications (cannot have any of the following).**		
Medication	NLM:ATC:A10BJ	Glucagon-like peptide-1 (GLP-1) analogues
Medication	NLM:ATC:A10BK	Sodium-glucose co-transporter 2 (SGLT2) inhibitors
Medication	NLM:RXNORM:2601723	tirzepatide
Medication	NLM:RXNORM:593411	sitagliptin
**#3: Without T1D, other diabetes, ESRD, HIV, transplant surgery or bariatric surgery (cannot have any of the following).**		
Diagnosis	UMLS:ICD10CM:E10	Type 1 diabetes mellitus
Diagnosis	UMLS:ICD10CM:E08	Diabetes mellitus due to underlying condition
Diagnosis	UMLS:ICD10CM:E09	Drug or chemical induced diabetes mellitus
Diagnosis	UMLS:ICD10CM:E13	Other specified diabetes mellitus
Diagnosis	UMLS:ICD10CM:N18.6	End stage renal disease
Diagnosis	UMLS:ICD10CM:B20	Human immunodeficiency virus [HIV] disease
Diagnosis	UMLS:ICD10CM:Z98.84	Bariatric surgery status
Procedure	UMLS:CPT:1007385	Bariatric Surgery Procedures
Diagnosis	UMLS:ICD10CM:Z94	Transplanted organ and tissue status
Procedure	UMLS:SNOMED:77465005	Transplantation
Diagnosis	UMLS:ICD10CM:T86	Complications of transplanted organs and tissue

**#4: Without prior exposure to the following medications (cannot have any of the following). #4 must be fulfilled within 6 months on or before #2.3**		
Medication	NLM:RXNORM:2601723	tirzepatide
Medication	NLM:ATC:A10BK	Sodium-glucose co-transporter 2 (SGLT2) inhibitors
Medication	NLM:ATC:A10BJ	Glucagon-like peptide-1 (GLP-1) analogues
Medication	NLM:RXNORM:4821	glipizide
Medication	NLM:RXNORM:593411	sitagliptin
**#5: No prior musculoskeletal events (cannot have any of the following). #5 must be fulfilled within 1 year before #2.3**		
Diagnosis	UMLS:ICD10CM:M80	Osteoporosis with current pathological fracture
Diagnosis	UMLS:ICD10CM:M81	Osteoporosis without current pathological fracture
Diagnosis	UMLS:ICD10CM:M17	Osteoarthritis of knee
Diagnosis	UMLS:ICD10CM:M16	Osteoarthritis of hip
Diagnosis	UMLS:ICD10CM:M10	Gout
Diagnosis	UMLS:ICD10CM:S72.0	Fracture of head and neck of femur
Diagnosis	UMLS:ICD10CM:S72.1	Pertrochanteric fracture
Diagnosis	UMLS:ICD10CM:S72.2	Subtrochanteric fracture of femur
Diagnosis	UMLS:ICD10CM:S22.0	Fracture of thoracic vertebra
Diagnosis	UMLS:ICD10CM:S32.0	Fracture of lumbar vertebra
Diagnosis	UMLS:ICD10CM:S52.5	Fracture of lower end of radius
Diagnosis	UMLS:ICD10CM:S52.6	Fracture of lower end of ulna
Diagnosis	UMLS:ICD10CM:S42.2	Fracture of upper end of humerus

**Usual care group (with T2D)**		
**#1: At least 20 years old (must have the following).**		
Demographics	Age	Age (at least 20 years)
**#2: Individuals with obesity and type 2 diabetes treated with metformin (#2.3 and #2.4 must be fulfilled after #2.1 and #2.2).<br>Date constraint: The terms in this group occurred between May 13, 2022, and Feb 28, 2026.**		
**#2.1: Individuals with obesity (must have any of the following).**		
Diagnosis	UMLS:ICD10CM:E66	Overweight and obesity
Laboratory	TNX:9083	BMI (at least 27.00 kg/m^2)
**#2.2: With type 2 diabetes (must have the following).**		
Diagnosis	UMLS:ICD10CM:E11	Type 2 diabetes mellitus
**#2.3: Receiving metformin (must have the following).**		
Medication	NLM:RXNORM:6809	metformin
**#2.4: No use of the following medications (cannot have any of the following).**		
Medication	NLM:ATC:A10BJ	Glucagon-like peptide-1 (GLP-1) analogues
Medication	NLM:ATC:A10BK	Sodium-glucose co-transporter 2 (SGLT2) inhibitors
Medication	NLM:RXNORM:2601723	tirzepatide
Medication	NLM:RXNORM:593411	sitagliptin
Medication	NLM:RXNORM:4821	glipizide
**#3: Without T1D, other diabetes, ESRD, HIV, transplant surgery or bariatric surgery (cannot have any of the following).**		
Diagnosis	UMLS:ICD10CM:E10	Type 1 diabetes mellitus
Diagnosis	UMLS:ICD10CM:E08	Diabetes mellitus due to underlying condition
Diagnosis	UMLS:ICD10CM:E09	Drug or chemical induced diabetes mellitus
Diagnosis	UMLS:ICD10CM:E13	Other specified diabetes mellitus
Diagnosis	UMLS:ICD10CM:N18.6	End stage renal disease
Diagnosis	UMLS:ICD10CM:B20	Human immunodeficiency virus [HIV] disease
Diagnosis	UMLS:ICD10CM:Z98.84	Bariatric surgery status

Procedure	UMLS:CPT:1007385	Bariatric Surgery Procedures
Diagnosis	UMLS:ICD10CM:Z94	Transplanted organ and tissue status
Procedure	UMLS:SNOMED:77465005	Transplantation
Diagnosis	UMLS:ICD10CM:T86	Complications of transplanted organs and tissue
**#4: Without prior exposure to the following medications (cannot have any of the following). #4 must be fulfilled within 6 months on or before #2.3**		
Medication	NLM:RXNORM:2601723	tirzepatide
Medication	NLM:ATC:A10BK	Sodium-glucose co-transporter 2 (SGLT2) inhibitors
Medication	NLM:ATC:A10BJ	Glucagon-like peptide-1 (GLP-1) analogues
Medication	NLM:RXNORM:4821	glipizide
Medication	NLM:RXNORM:593411	sitagliptin
**#5: No prior musculoskeletal events (cannot have any of the following). #5 must be fulfilled within 1 year before #2.3**		
Diagnosis	UMLS:ICD10CM:M80	Osteoporosis with current pathological fracture
Diagnosis	UMLS:ICD10CM:M81	Osteoporosis without current pathological fracture
Diagnosis	UMLS:ICD10CM:M17	Osteoarthritis of knee
Diagnosis	UMLS:ICD10CM:M16	Osteoarthritis of hip
Diagnosis	UMLS:ICD10CM:M10	Gout
Diagnosis	UMLS:ICD10CM:S72.0	Fracture of head and neck of femur
Diagnosis	UMLS:ICD10CM:S72.1	Pertrochanteric fracture
Diagnosis	UMLS:ICD10CM:S72.2	Subtrochanteric fracture of femur
Diagnosis	UMLS:ICD10CM:S22.0	Fracture of thoracic vertebra
Diagnosis	UMLS:ICD10CM:S32.0	Fracture of lumbar vertebra
Diagnosis	UMLS:ICD10CM:S52.5	Fracture of lower end of radius
Diagnosis	UMLS:ICD10CM:S52.6	Fracture of lower end of ulna
Diagnosis	UMLS:ICD10CM:S42.2	Fracture of upper end of humerus

**Tirzepatide group (without T2D)**		
**#1: At least 20 years old (must have the following).**		
Demographics	Age	Age (at least 20 years)
**#2: Individuals with obesity treated with tirzepatide (#2.2 and #2.3 must be fulfilled after #2.1).<br>Date constraint: The terms in this group occurred between Nov 8, 2023, and Feb 28, 2026.**		
**#2.1: Individuals with obesity (must have any of the following).**		
Diagnosis	UMLS:ICD10CM:E66	Overweight and obesity
Laboratory	TNX:9083	BMI (at least 27.00 kg/m^2)
**#2.2: Receiving tirzepatide (must have the following).**		
Medication	NLM:RXNORM:2601723	tirzepatide
**#2.3: No use of the following medications (cannot have any of the following).**		
Medication	NLM:ATC:A10BJ	Glucagon-like peptide-1 (GLP-1) analogues
Medication	NLM:RXNORM:8152	phentermine
Medication	NLM:RXNORM:7243	naltrexone
Medication	NLM:RXNORM:42347	bupropion
Medication	NLM:RXNORM:38404	topiramate
**#3: Without diabetes, ESRD, HIV, transplant surgery or bariatric surgery (cannot have any of the following).**		
Diagnosis	UMLS:ICD10CM:E08-E13	Diabetes mellitus
Diagnosis	UMLS:ICD10CM:N18.6	End stage renal disease
Diagnosis	UMLS:ICD10CM:B20	Human immunodeficiency virus [HIV] disease
Diagnosis	UMLS:ICD10CM:Z98.84	Bariatric surgery status
Procedure	UMLS:CPT:1007385	Bariatric Surgery Procedures

Diagnosis	UMLS:ICD10CM:Z94	Transplanted organ and tissue status
Procedure	UMLS:SNOMED:77465005	Transplantation
Diagnosis	UMLS:ICD10CM:T86	Complications of transplanted organs and tissue
**#4: Without prior exposure to the following medications (cannot have any of the following). #4 must be fulfilled within 6 months on or before #2.2**		
Medication	NLM:RXNORM:2601723	tirzepatide
Medication	NLM:ATC:A10BJ	Glucagon-like peptide-1 (GLP-1) analogues
Medication	NLM:RXNORM:8152	phentermine
Medication	NLM:RXNORM:7243	naltrexone
Medication	NLM:RXNORM:42347	bupropion
Medication	NLM:RXNORM:38404	topiramate
**#5: No prior musculoskeletal events (cannot have any of the following). #5 must be fulfilled within 1 year before #2.2**		
Diagnosis	UMLS:ICD10CM:M80	Osteoporosis with current pathological fracture
Diagnosis	UMLS:ICD10CM:M81	Osteoporosis without current pathological fracture
Diagnosis	UMLS:ICD10CM:M17	Osteoarthritis of knee
Diagnosis	UMLS:ICD10CM:M16	Osteoarthritis of hip
Diagnosis	UMLS:ICD10CM:M10	Gout
Diagnosis	UMLS:ICD10CM:S72.0	Fracture of head and neck of femur
Diagnosis	UMLS:ICD10CM:S72.1	Pertrochanteric fracture
Diagnosis	UMLS:ICD10CM:S72.2	Subtrochanteric fracture of femur
Diagnosis	UMLS:ICD10CM:S22.0	Fracture of thoracic vertebra
Diagnosis	UMLS:ICD10CM:S32.0	Fracture of lumbar vertebra
Diagnosis	UMLS:ICD10CM:S52.5	Fracture of lower end of radius
Diagnosis	UMLS:ICD10CM:S52.6	Fracture of lower end of ulna
Diagnosis	UMLS:ICD10CM:S42.2	Fracture of upper end of humerus

**Semaglutide group (without T2D)**		
**#1: At least 20 years old (must have the following).**		
Demographics	Age	Age (at least 20 years)
**#2: Individuals with obesity treated with semaglutide (#2.2 and #2.3 must be fulfilled after #2.1).<br>Date constraint: The terms in this group occurred between Nov 8, 2023, and Feb 28, 2026.**		
**#2.1: Individuals with obesity (must have any of the following).**		
Diagnosis	UMLS:ICD10CM:E66	Overweight and obesity
Laboratory	TNX:9083	BMI (at least 27.00 kg/m^2)
**#2.2: Receiving semaglutide (must have the following).**		
Medication	NLM:ATC:A10BJ	Glucagon-like peptide-1 (GLP-1) analogues (Brand: Ozempic or Rybelsus or Wegovy)
**#2.3: No use of the following medications (cannot have any of the following).**		
Medication	NLM:RXNORM:2601723	tirzepatide
Medication	NLM:RXNORM:8152	phentermine
Medication	NLM:RXNORM:7243	naltrexone
Medication	NLM:RXNORM:42347	bupropion
Medication	NLM:RXNORM:38404	topiramate
**#3: Without diabetes, ESRD, HIV, transplant surgery or bariatric surgery (cannot have any of the following).**		
Diagnosis	UMLS:ICD10CM:E08-E13	Diabetes mellitus
Diagnosis	UMLS:ICD10CM:N18.6	End stage renal disease
Diagnosis	UMLS:ICD10CM:B20	Human immunodeficiency virus [HIV] disease
Diagnosis	UMLS:ICD10CM:Z98.84	Bariatric surgery status

Procedure	UMLS:CPT:1007385	Bariatric Surgery Procedures
Diagnosis	UMLS:ICD10CM:Z94	Transplanted organ and tissue status
Procedure	UMLS:SNOMED:77465005	Transplantation
Diagnosis	UMLS:ICD10CM:T86	Complications of transplanted organs and tissue
**#4: Without prior exposure to the following medications (cannot have any of the following). #4 must be fulfilled within 6 months on or before #2.2**		
Medication	NLM:RXNORM:2601723	tirzepatide
Medication	NLM:ATC:A10BJ	Glucagon-like peptide-1 (GLP-1) analogues
Medication	NLM:RXNORM:8152	phentermine
Medication	NLM:RXNORM:7243	naltrexone
Medication	NLM:RXNORM:42347	bupropion
Medication	NLM:RXNORM:38404	topiramate
**#5: No prior musculoskeletal events (cannot have any of the following). #5 must be fulfilled within 1 year before #2.2**		
Diagnosis	UMLS:ICD10CM:M80	Osteoporosis with current pathological fracture
Diagnosis	UMLS:ICD10CM:M81	Osteoporosis without current pathological fracture
Diagnosis	UMLS:ICD10CM:M17	Osteoarthritis of knee
Diagnosis	UMLS:ICD10CM:M16	Osteoarthritis of hip
Diagnosis	UMLS:ICD10CM:M10	Gout
Diagnosis	UMLS:ICD10CM:S72.0	Fracture of head and neck of femur
Diagnosis	UMLS:ICD10CM:S72.1	Pertrochanteric fracture
Diagnosis	UMLS:ICD10CM:S72.2	Subtrochanteric fracture of femur
Diagnosis	UMLS:ICD10CM:S22.0	Fracture of thoracic vertebra
Diagnosis	UMLS:ICD10CM:S32.0	Fracture of lumbar vertebra
Diagnosis	UMLS:ICD10CM:S52.5	Fracture of lower end of radius
Diagnosis	UMLS:ICD10CM:S52.6	Fracture of lower end of ulna
Diagnosis	UMLS:ICD10CM:S42.2	Fracture of upper end of humerus

**Naltrexone/bupropion group (without T2D)**		
**#1: At least 20 years old (must have the following).**		
Demographics	Age	Age (at least 20 years)
**#2: Individuals with obesity treated with naltrexone/bupropion (#2.2 and #2.3 must be fulfilled after #2.1).<br>Date constraint: The terms in this group occurred between Nov 8, 2023, and Feb 28, 2026.**		
**#2.1: Individuals with obesity (must have any of the following).**		
Diagnosis	UMLS:ICD10CM:E66	Overweight and obesity
Laboratory	TNX:9083	BMI (at least 27.00 kg/m^2)
**#2.2: Receiving naltrexone/bupropion (must have the following).**		
Medication	NLM:RXNORM:7243	naltrexone
Medication	NLM:RXNORM:42347	bupropion
**#2.3: No use of the following medications (cannot have any of the following).**		
Medication	NLM:RXNORM:2601723	tirzepatide
Medication	NLM:ATC:A10BJ	Glucagon-like peptide-1 (GLP-1) analogues
Medication	NLM:RXNORM:8152	phentermine
Medication	NLM:RXNORM:38404	topiramate
**#3: Without diabetes, ESRD, HIV, transplant surgery or bariatric surgery (cannot have any of the following).**		
Diagnosis	UMLS:ICD10CM:E08-E13	Diabetes mellitus
Diagnosis	UMLS:ICD10CM:N18.6	End stage renal disease
Diagnosis	UMLS:ICD10CM:B20	Human immunodeficiency virus [HIV] disease

Diagnosis	UMLS:ICD10CM:Z98.84	Bariatric surgery status
Procedure	UMLS:CPT:1007385	Bariatric Surgery Procedures
Diagnosis	UMLS:ICD10CM:Z94	Transplanted organ and tissue status
Procedure	UMLS:SNOMED:77465005	Transplantation
Diagnosis	UMLS:ICD10CM:T86	Complications of transplanted organs and tissue
**#4: Without prior exposure to the following medications (cannot have any of the following). #4 must be fulfilled within 6 months on or before #2.2**		
Medication	NLM:RXNORM:2601723	tirzepatide
Medication	NLM:ATC:A10BJ	Glucagon-like peptide-1 (GLP-1) analogues
Medication	NLM:RXNORM:8152	phentermine
Medication	NLM:RXNORM:7243	naltrexone
Medication	NLM:RXNORM:42347	bupropion
Medication	NLM:RXNORM:38404	topiramate
**#5: No prior musculoskeletal events (cannot have any of the following). #5 must be fulfilled within 1 year before #2.2**		
Diagnosis	UMLS:ICD10CM:M80	Osteoporosis with current pathological fracture
Diagnosis	UMLS:ICD10CM:M81	Osteoporosis without current pathological fracture
Diagnosis	UMLS:ICD10CM:M17	Osteoarthritis of knee
Diagnosis	UMLS:ICD10CM:M16	Osteoarthritis of hip
Diagnosis	UMLS:ICD10CM:M10	Gout
Diagnosis	UMLS:ICD10CM:S72.0	Fracture of head and neck of femur
Diagnosis	UMLS:ICD10CM:S72.1	Pertrochanteric fracture
Diagnosis	UMLS:ICD10CM:S72.2	Subtrochanteric fracture of femur
Diagnosis	UMLS:ICD10CM:S22.0	Fracture of thoracic vertebra
Diagnosis	UMLS:ICD10CM:S32.0	Fracture of lumbar vertebra
Diagnosis	UMLS:ICD10CM:S52.5	Fracture of lower end of radius
Diagnosis	UMLS:ICD10CM:S52.6	Fracture of lower end of ulna
Diagnosis	UMLS:ICD10CM:S42.2	Fracture of upper end of humerus

**Phentermine/topiramate group (without T2D)**		
**#1: At least 20 years old (must have the following).**		
Demographics	Age	Age (at least 20 years)
**#2: Individuals with obesity treated with phentermine/topiramate (#2.2 and #2.3 must be fulfilled after #2.1).**<br>**Date constraint: The terms in this group occurred between Nov 8, 2023, and Feb 28, 2026.**		
**#2.1: Individuals with obesity (must have any of the following).**		
Diagnosis	UMLS:ICD10CM:E66	Overweight and obesity
Laboratory	TNX:9083	BMI (at least 27.00 kg/m^2)
**#2.2: Receiving phentermine/topiramate (must have the following).**		
Medication	NLM:RXNORM:8152	phentermine
Medication	NLM:RXNORM:38404	topiramate
**#2.3: No use of the following medications (cannot have any of the following).**		
Medication	NLM:RXNORM:2601723	tirzepatide
Medication	NLM:ATC:A10BJ	Glucagon-like peptide-1 (GLP-1) analogues
Medication	NLM:RXNORM:7243	naltrexone
Medication	NLM:RXNORM:42347	bupropion
**#3: Without diabetes, ESRD, HIV, transplant surgery or bariatric surgery (cannot have any of the following).**		
Diagnosis	UMLS:ICD10CM:E08-E13	Diabetes mellitus
Diagnosis	UMLS:ICD10CM:N18.6	End stage renal disease

Diagnosis	UMLS:ICD10CM:B20	Human immunodeficiency virus [HIV] disease
Diagnosis	UMLS:ICD10CM:Z98.84	Bariatric surgery status
Procedure	UMLS:CPT:1007385	Bariatric Surgery Procedures
Diagnosis	UMLS:ICD10CM:Z94	Transplanted organ and tissue status
Procedure	UMLS:SNOMED:77465005	Transplantation
Diagnosis	UMLS:ICD10CM:T86	Complications of transplanted organs and tissue
**#4: Without prior exposure to the following medications (cannot have any of the following). #4 must be fulfilled within 6 months on or before #2.2**		
Medication	NLM:RXNORM:2601723	tirzepatide
Medication	NLM:ATC:A10BJ	Glucagon-like peptide-1 (GLP-1) analogues
Medication	NLM:RXNORM:8152	phentermine
Medication	NLM:RXNORM:7243	naltrexone
Medication	NLM:RXNORM:42347	bupropion
Medication	NLM:RXNORM:38404	topiramate
**#5: No prior musculoskeletal events (cannot have any of the following). #5 must be fulfilled within 1 year before #2.2**		
Diagnosis	UMLS:ICD10CM:M80	Osteoporosis with current pathological fracture
Diagnosis	UMLS:ICD10CM:M81	Osteoporosis without current pathological fracture
Diagnosis	UMLS:ICD10CM:M17	Osteoarthritis of knee
Diagnosis	UMLS:ICD10CM:M16	Osteoarthritis of hip
Diagnosis	UMLS:ICD10CM:M10	Gout
Diagnosis	UMLS:ICD10CM:S72.0	Fracture of head and neck of femur
Diagnosis	UMLS:ICD10CM:S72.1	Pertrochanteric fracture
Diagnosis	UMLS:ICD10CM:S72.2	Subtrochanteric fracture of femur
Diagnosis	UMLS:ICD10CM:S22.0	Fracture of thoracic vertebra
Diagnosis	UMLS:ICD10CM:S32.0	Fracture of lumbar vertebra
Diagnosis	UMLS:ICD10CM:S52.5	Fracture of lower end of radius
Diagnosis	UMLS:ICD10CM:S52.6	Fracture of lower end of ulna
Diagnosis	UMLS:ICD10CM:S42.2	Fracture of upper end of humerus

**Phentermine group (without T2D)**		
**#1: At least 20 years old (must have the following).**		
Demographics	Age	Age (at least 20 years)
**#2: Individuals with obesity treated with phentermine (#2.2 and #2.3 must be fulfilled after #2.1). Date constraint: The terms in this group occurred between Nov 8, 2023, and Feb 28, 2026.**		
**#2.1: Individuals with obesity (must have any of the following).**		
Diagnosis	UMLS:ICD10CM:E66	Overweight and obesity
Laboratory	TNX:9083	BMI (at least 27.00 kg/m^2)
**#2.2: Receiving phentermine (must have the following).**		
Medication	NLM:RXNORM:8152	phentermine
**#2.3: No use of the following medications (cannot have any of the following).**		
Medication	NLM:RXNORM:2601723	tirzepatide
Medication	NLM:ATC:A10BJ	Glucagon-like peptide-1 (GLP-1) analogues
Medication	NLM:RXNORM:7243	naltrexone
Medication	NLM:RXNORM:42347	bupropion
Medication	NLM:RXNORM:38404	topiramate
**#3: Without diabetes, ESRD, HIV, transplant surgery or bariatric surgery (cannot have any of the following).**		
Diagnosis	UMLS:ICD10CM:E08-E13	Diabetes mellitus
Diagnosis	UMLS:ICD10CM:N18.6	End stage renal disease

Diagnosis	UMLS:ICD10CM:B20	Human immunodeficiency virus [HIV] disease
Diagnosis	UMLS:ICD10CM:Z98.84	Bariatric surgery status
Procedure	UMLS:CPT:1007385	Bariatric Surgery Procedures
Diagnosis	UMLS:ICD10CM:Z94	Transplanted organ and tissue status
Procedure	UMLS:SNOMED:77465005	Transplantation
Diagnosis	UMLS:ICD10CM:T86	Complications of transplanted organs and tissue
**#4: Without prior exposure to the following medications (cannot have any of the following). #4 must be fulfilled within 6 months on or before #2.2**		
Medication	NLM:RXNORM:2601723	tirzepatide
Medication	NLM:ATC:A10BJ	Glucagon-like peptide-1 (GLP-1) analogues
Medication	NLM:RXNORM:8152	phentermine
Medication	NLM:RXNORM:7243	naltrexone
Medication	NLM:RXNORM:42347	bupropion
Medication	NLM:RXNORM:38404	topiramate
**#5: No prior musculoskeletal events (cannot have any of the following). #5 must be fulfilled within 1 year before #2.2**		
Diagnosis	UMLS:ICD10CM:M80	Osteoporosis with current pathological fracture
Diagnosis	UMLS:ICD10CM:M81	Osteoporosis without current pathological fracture
Diagnosis	UMLS:ICD10CM:M17	Osteoarthritis of knee
Diagnosis	UMLS:ICD10CM:M16	Osteoarthritis of hip
Diagnosis	UMLS:ICD10CM:M10	Gout
Diagnosis	UMLS:ICD10CM:S72.0	Fracture of head and neck of femur
Diagnosis	UMLS:ICD10CM:S72.1	Pertrochanteric fracture
Diagnosis	UMLS:ICD10CM:S72.2	Subtrochanteric fracture of femur
Diagnosis	UMLS:ICD10CM:S22.0	Fracture of thoracic vertebra
Diagnosis	UMLS:ICD10CM:S32.0	Fracture of lumbar vertebra
Diagnosis	UMLS:ICD10CM:S52.5	Fracture of lower end of radius
Diagnosis	UMLS:ICD10CM:S52.6	Fracture of lower end of ulna
Diagnosis	UMLS:ICD10CM:S42.2	Fracture of upper end of humerus

**Usual care group (without T2D)**		
**#1: At least 20 years old (must have the following).**		
Demographics	Age	Age (at least 20 years)
**#2: Individuals with obesity treated with metformin (#2.2 and #2.3 must be fulfilled after #2.1).<br>Date constraint: The terms in this group occurred between Nov 8, 2023, and Feb 28, 2026.**		
**#2.1: Individuals with obesity (must have any of the following).**		
Diagnosis	UMLS:ICD10CM:E66	Overweight and obesity
Laboratory	TNX:9083	BMI (at least 27.00 kg/m^2)
**#2.2: Receiving metformin (must have the following).**		
Medication	NLM:RXNORM:6809	metformin
**#2.3: No use of the following medications (cannot have any of the following).**		
Medication	NLM:RXNORM:2601723	tirzepatide
Medication	NLM:ATC:A10BJ	Glucagon-like peptide-1 (GLP-1) analogues
Medication	NLM:RXNORM:7243	naltrexone
Medication	NLM:RXNORM:42347	bupropion
Medication	NLM:RXNORM:8152	phentermine
Medication	NLM:RXNORM:38404	topiramate
**#3: Without diabetes, ESRD, HIV, transplant surgery or bariatric surgery (cannot have any of the following).**		
Diagnosis	UMLS:ICD10CM:E08-E13	Diabetes mellitus

Diagnosis	UMLS:ICD10CM:N18.6	End stage renal disease
Diagnosis	UMLS:ICD10CM:B20	Human immunodeficiency virus [HIV] disease
Diagnosis	UMLS:ICD10CM:Z98.84	Bariatric surgery status
Procedure	UMLS:CPT:1007385	Bariatric Surgery Procedures
Diagnosis	UMLS:ICD10CM:Z94	Transplanted organ and tissue status
Procedure	UMLS:SNOMED:77465005	Transplantation
Diagnosis	UMLS:ICD10CM:T86	Complications of transplanted organs and tissue
**#4: Without prior exposure to the following medications (cannot have any of the following). #4 must be fulfilled within 6 months on or before #2.2**		
Medication	NLM:RXNORM:2601723	tirzepatide
Medication	NLM:ATC:A10BJ	Glucagon-like peptide-1 (GLP-1) analogues
Medication	NLM:RXNORM:8152	phentermine
Medication	NLM:RXNORM:7243	naltrexone
Medication	NLM:RXNORM:42347	bupropion
Medication	NLM:RXNORM:38404	topiramate
**#5: No prior musculoskeletal events (cannot have any of the following). #5 must be fulfilled within 1 year before #2.2**		
Diagnosis	UMLS:ICD10CM:M80	Osteoporosis with current pathological fracture
Diagnosis	UMLS:ICD10CM:M81	Osteoporosis without current pathological fracture
Diagnosis	UMLS:ICD10CM:M17	Osteoarthritis of knee
Diagnosis	UMLS:ICD10CM:M16	Osteoarthritis of hip
Diagnosis	UMLS:ICD10CM:M10	Gout
Diagnosis	UMLS:ICD10CM:S72.0	Fracture of head and neck of femur
Diagnosis	UMLS:ICD10CM:S72.1	Pertrochanteric fracture
Diagnosis	UMLS:ICD10CM:S72.2	Subtrochanteric fracture of femur
Diagnosis	UMLS:ICD10CM:S22.0	Fracture of thoracic vertebra
Diagnosis	UMLS:ICD10CM:S32.0	Fracture of lumbar vertebra
Diagnosis	UMLS:ICD10CM:S52.5	Fracture of lower end of radius
Diagnosis	UMLS:ICD10CM:S52.6	Fracture of lower end of ulna
Diagnosis	UMLS:ICD10CM:S42.2	Fracture of upper end of humerus

Demographic, diagnostic, laboratory, and medication codes used in the definition of covariates.

Category	Code	Description
Demographics	AI	Age at index
Demographics	F	Female
Demographics	M	Male
Demographics	2135-2	Hispanic or Latino
Demographics	2186-5	Not Hispanic or Latino
Demographics	2106-3	White
Demographics	2054-5	Black or African American
Demographics	2131-1	Other Race
Diagnosis	Z55-Z65	Persons with potential health hazards related to socioeconomic and psychosocial circumstances
Visit	Visit	Visit
Visit	AMB	Visit: Ambulatory
Visit	EMER	Visit: Emergency
Diagnosis	J45	Asthma
Diagnosis	F32	Depressive episode
Diagnosis	G47	Sleep disorders
Diagnosis	H25	Age-related cataract
Diagnosis	H40-H42	Glaucoma
Diagnosis	C00-D49	Neoplasms
Diagnosis	D80-D89	Certain disorders involving the immune mechanism
Diagnosis	E00-E07	Disorders of thyroid gland
Diagnosis	E78	Disorders of lipoprotein metabolism and other lipidemias
Diagnosis	F31	Bipolar disorder
Diagnosis	F33	Major depressive disorder, recurrent
Diagnosis	F51	Sleep disorders not due to a substance or known physiological condition
Diagnosis	G20-G26	Extrapyramidal and movement disorders
Diagnosis	G89.2	Chronic pain, not elsewhere classified
Diagnosis	K70-K77	Diseases of liver
Diagnosis	L40	Psoriasis
Diagnosis	R40	Somnolence, stupor and coma
Diagnosis	R41	Other symptoms and signs involving cognitive functions and awareness
Diagnosis	U07.1	COVID-19
Diagnosis	Z79.52	Long term (current) use of systemic steroids
Diagnosis	M85	Other disorders of bone density and structure
Diagnosis	Z13.820	Encounter for screening for osteoporosis
Diagnosis	Z91.81	History of falling
Diagnosis	W00-W19	Slipping, tripping, stumbling and falls
Diagnosis	R29.6	Repeated falls
Diagnosis	Z87.81	Personal history of (healed) traumatic fracture
Diagnosis	R80, R80.9	Proteinuria
Procedure	1014948	Dual-energy X-ray absorptiometry (DXA), bone density study, 1 or more sites
Medication	CV900	CARDIOVASCULAR AGENTS,OTHER
Medication	H03	THYROID THERAPY
Medication	L01	ANTINEOPLASTIC AGENTS
Medication	L04	IMMUNOSUPPRESSANTS

Category	Code	Description
Medication	N02C	ANTIMIGRAINE PREPARATIONS
Medication	N03	ANTIEPILEPTICS
Medication	N04	ANTI-PARKINSON DRUGS
Medication	N05A	ANTIPSYCHOTICS
Medication	N06AA	Non-selective monoamine reuptake inhibitors
Medication	N06AX	Other antidepressants
Medication	N07B	DRUGS USED IN ADDICTIVE DISORDERS
Medication	P	ANTIPARASITIC PRODUCTS, INSECTICIDES AND REPELLENTS
Medication	R06	ANTIHISTAMINES FOR SYSTEMIC USE
Medication	213	SARS-CoV-2 (COVID-19) Vaccine
Medication	M05BA	Bisphosphonates
Medication	993449	denosumab
Medication	2123126	romosozumab
Medication	M05BX	Other drugs affecting bone structure and mineralization
Medication	N06AB	Selective serotonin reuptake inhibitors
Medication	A11CC	Vitamin D and analogues
Medication	D06B	CHEMOTHERAPEUTICS FOR TOPICAL USE
Medication	L02BG	Aromatase inhibitors
Medication	HS051	GLUCOCORTICOIDS
Medication	46041	alendronate
Medication	55685	risedronic acid
Medication	115264	ibandronate
Medication	77655	zoledronic acid
Medication	32915	teriparatide
Medication	1921069	abaloparatide
Medication	6809	metformin
Laboratory	9083	BMI
Laboratory	9002	Cholesterol in LDL [Mass/volume] in Serum or Plasma
Laboratory	9004	Triglyceride [Mass/volume] in Serum, Plasma or Blood
Laboratory	9037	Hemoglobin A1c/Hemoglobin.total in Blood
Laboratory	8001	Glomerular filtration rate/1.73 sq M.predicted [Volume Rate/Area] in Serum, Plasma or Blood by Creatinine-based formula (MDRD)
Laboratory	9085	Blood Pressure, Systolic

Diagnostic codes used in the definition of outcomes.

Category	Code	Description
**#1**: **MOF** (have any of the following)		
Diagnosis	UMLS:ICD10CM:S72.0	Fracture of head and neck of femur
Diagnosis	UMLS:ICD10CM:S72.1	Pertrochanteric fracture
Diagnosis	UMLS:ICD10CM:S72.2	Subtrochanteric fracture of femur
Diagnosis	UMLS:ICD10CM:S22.0	Fracture of thoracic vertebra
Diagnosis	UMLS:ICD10CM:S32.0	Fracture of lumbar vertebra
Diagnosis	UMLS:ICD10CM:S52.5	Fracture of lower end of radius
Diagnosis	UMLS:ICD10CM:S52.6	Fracture of lower end of ulna
Diagnosis	UMLS:ICD10CM:S42.2	Fracture of upper end of humerus
**#2: Osteoporosis** (have any of the following)		
Diagnosis	UMLS:ICD10CM:M80	Osteoporosis with current pathological fracture
Diagnosis	UMLS:ICD10CM:M81	Osteoporosis without current pathological fracture
**#3: Osteoarthritis of knee** (have the following)		
Diagnosis	UMLS:ICD10CM:M17	Osteoarthritis of knee
**#4: Osteoarthritis of hip** (have the following)		
Diagnosis	UMLS:ICD10CM:M16	Osteoarthritis of hip
**#5: Gout** (have the following)		
Diagnosis	UMLS:ICD10CM:M10	Gout
**#6: Hip fracture surgery/repair** (have any of the following)		
Procedure	UMLS:CPT:27235	Percutaneous skeletal fixation of femoral fracture, proximal end, neck
Procedure	UMLS:CPT:27244	Treatment of intertrochanteric, peritrochanteric, or subtrochanteric femoral fracture; with plate/screw type implant, with or without cerclage
Procedure	UMLS:CPT:27245	Treatment of intertrochanteric, peritrochanteric, or subtrochanteric femoral fracture; with intramedullary implant, with or without interlocking screws and/or cerclage
Procedure	UMLS:CPT:1006747	Hemodialysis Access, Intervascular Cannulation for Extracorporeal Circulation, or Shunt Insertion Procedures on Arteries and Veins
Procedure	UMLS:ICD10PCS:0QS6	Lower Bones / Reposition / Upper Femur, Right
Procedure	UMLS: ICD10PCS:0QS7	Lower Bones / Reposition / Upper Femur, Left
**#7: Alendronate** (have the following)		
Medication	NLM:RXNORM:46041	alendronate
**#8: Denosumab** (have any of the following)		
Medication	NLM:RXNORM:993449	denosumab
Medication	HCPCS:J0897	Injection, denosumab, 1 mg
Medication	HCPCS:Q5136	Injection, denosumab-bodz (jubbonti/wyost), biosimilar, 1 mg
**#9: Zoledronic acid** (have any of the following)		
Medication	NLM:RXNORM:77655	zoledronic acid
Medication	HCPCS:J3489	Injection, zoledronic acid, 1 mg
**#10: Healthcare utilization** (have any of the following)		
Procedure	UMLS:CPT:99217	Observation care discharge day management (This code is to be utilized to report all services provided to a patient on discharge from outpatient hospital "observation status" if the discharge is on other than the initial date of "observation status." To report services to a patient designated as observation status" or "inpatient status" and discharged on the same date (deprecated 2023)

Procedure	UMLS:CPT:99218	Initial observation care (deprecated 2023)
Procedure	UMLS:CPT:99219	Initial observation care (deprecated 2023)
Procedure	UMLS:CPT:99220	Initial observation care (deprecated 2023)
Procedure	UMLS:CPT:99221	Initial hospital inpatient or observation care, per day, for the evaluation and management of a patient, which requires a medically appropriate history and/or examination and straightforward or low level medical decision making. When using total time on the date of the encounter for code selection, 40 minutes must be met or exceeded.
Procedure	UMLS:CPT:99222	Initial hospital inpatient or observation care, per day, for the evaluation and management of a patient, which requires a medically appropriate history and/or examination and moderate level of medical decision making. When using total time on the date of the encounter for code selection, 55 minutes must be met or exceeded.
Procedure	UMLS:CPT:99223	Initial hospital inpatient or observation care, per day, for the evaluation and management of a patient, which requires a medically appropriate history and/or examination and high level of medical decision making. When using total time on the date of the encounter for code selection, 75 minutes must be met or exceeded.
Procedure	UMLS:CPT:99231	Subsequent hospital inpatient or observation care, per day, for the evaluation and management of a patient, which requires a medically appropriate history and/or examination and straightforward or low level of medical decision making. When using total time on the date of the encounter for code selection, 25 minutes must be met or exceeded.
Procedure	UMLS:CPT:99232	Subsequent hospital inpatient or observation care, per day, for the evaluation and management of a patient, which requires a medically appropriate history and/or examination and moderate level of medical decision making. When using total time on the date of the encounter for code selection, 35 minutes must be met or exceeded.
Procedure	UMLS:CPT:99233	Subsequent hospital inpatient or observation care, per day, for the evaluation and management of a patient, which requires a medically appropriate history and/or examination and high level of medical decision making. When using total time on the date of the encounter for code selection, 50 minutes must be met or exceeded.
Procedure	UMLS:CPT:99234	Hospital inpatient or observation care, for the evaluation and management of a patient incuding admission and discharge on the same date, which requires a medically appropriate history and/or examination and straightforward or low level of medical decision making. When using total time on the date of the encounter for code selection, 45 minutes must be met or exceeded.
Procedure	UMLS:CPT:99235	Hospital inpatient or observation care, for the evaluation and management of a patient including admission and discharge on the same date, which requires a medically appropriate history and/or examination and moderate level of medical decision making. When using total time on the date of the encounter for code selection, 70 minutes must be met or exceeded.
Procedure	UMLS:CPT:99236	Hospital inpatient or observation care, for the evaluation and management of a patient including admission and discharge on the same date, which requires a medically

		appropriate history and/or examination and high level of medical decision making. When using total time on the date of the encounter for code selection, 85 minutes must be met or exceeded.
Procedure	UMLS:CPT:99238	Hospital inpatient or observation discharge day management; 30 minutes or less on the date of the encounter
Procedure	UMLS:CPT:99239	Hospital inpatient or observation discharge day management; more than 30 minutes on the date of the encounter
**#11: Negative control outcomes**		
Diagnosis	UMLS:ICD10CM:K40-K46	Hernia
Diagnosis	UMLS:ICD10CM:M54.16	Radiculopathy, lumbar region
Diagnosis	UMLS:ICD10CM:L60.0	Ingrowing nail
Diagnosis	UMLS:ICD10CM:H60	Otitis externa
Diagnosis	UMLS:ICD10CM:H90	Conductive and sensorineural hearing loss
Diagnosis	UMLS:ICD10CM:C44	Other and unspecified malignant neoplasm of skin

Supplementary Results

Tirzepatide results:

In the T2D cohort (Table S23), tirzepatide showed directionally consistent MOF findings versus active comparators: versus empagliflozin (HR 0.64, 95% CI: 0.54–0.76, $p < 0.001$), versus glipizide (HR 0.63, 0.52–0.77, $p < 0.001$), and versus usual care (HR 0.71, 0.61–0.82, $p < 0.001$). The tirzepatide versus sitagliptin comparison was HR 0.76 (0.60–0.96, $p = 0.023$). When compared directly with the GLP-1 RA class, tirzepatide showed no significant MOF difference (HR 0.92, 95% CI: 0.79–1.07, $p = 0.259$).

In the non-T2D cohort (Table S24), tirzepatide was not associated with a significant MOF difference versus any comparator: versus usual care (HR 0.95, 0.74–1.22), versus phentermine (HR 1.06, 0.77–1.45), versus naltrexone-bupropion (HR 0.92, 0.56–1.51), and versus phentermine-topiramate (HR 0.89, 0.49–1.62). However, tirzepatide versus usual care was associated with a higher gout hazard (HR 1.44, 95% CI: 1.13–1.83, adjusted p = 0.016) in the non-T2D cohort. The tirzepatide versus GLP-1 RA comparison for MOF was HR 1.02 (0.86–1.21, $p = 0.804$).

The limited tirzepatide sample sizes and shorter observation period restrict the interpretability of these findings. Tirzepatide received FDA approval for obesity in November 2023, and early-adopter populations may differ systematically from later users in ways not fully captured by propensity score matching.

Bone-active medication uses and healthcare utilization

Bone-active medication use (alendronate, denosumab, zoledronic acid) was analyzed separately from osteoporosis diagnosis to assess post-index prescribing patterns (Figure S6). Semaglutide was not associated with significant differences in bone-active medication use versus any comparator in either cohort (all $p > 0.05$), indicating that the observed osteoporosis findings were independent of differential treatment intensity. Healthcare utilization was consistently lower with semaglutide in the T2D cohort (HR 0.62–0.78, all $p < 0.001$).

Longitudinal analysis of BMI and HbA1c

BMI reduction varied between population groups. In obesity without T2D, semaglutide achieved 6.33% BMI reduction over five years versus naltrexone/bupropion's 3.58% (Figure S9). In T2D, semaglutide's 4.75% reduction exceeded sitagliptin's 2.98%. The stronger effect in people without T2D (difference of 1.58%) suggests possible metabolic resistance in diabetic states. HbA1c control showed marked differences between groups (Figure S10). In T2D, semaglutide reduced HbA1c by 11.59% versus sitagliptin's 8.65%. The contrast between people with and without T2D response was striking – semaglutide's effect was 5.3-fold greater in people with T2D (11.59% vs 1.83% reduction). Meanwhile, naltrexone/bupropion increased HbA1c slightly (0.95%) in n people without T2D.

Supplementary Table 1. Protocol components of the target trial and their emulation in the TriNetX database analysis of semaglutide versus conventional therapies for skeletal outcomes in people with obesity.

Protocol component	Target trial	Emulated trial
**Aim**	Compare the risk of skeletal outcomes after initiating semaglutide versus prespecified comparators in people with obesity, stratified by T2D status.	Same objective, operationalized with the TriNetX US Collaborative Network (≈65 healthcare organizations, ≈120 million patients).
**Eligibility criteria**	**Inclusion:**<br>• Age ≥20 years<br>• Obesity (BMI ≥27 kg/m²)<br>• Two cohorts: obesity with T2D; obesity without T2D<br>• New user of index or comparator drug<br><br>**Exclusion:**<br>• T1D (E10), other diabetes (E08, E09, E13)<br>• Prior bariatric surgery, organ transplant<br>• HIV, end-stage renal disease	Same criteria applied via ICD-10 codes, procedure codes, and laboratory values in TriNetX.<br><br>• Obesity: E66 plus documented BMI ≥27 kg/m²<br>• T2D: E11<br>• 6-month washout: no prescription for the index drug or same-class medication before T_0<br>• No prior study outcomes within 1 year before T_0
**Treatment strategies**	**T2D cohort:**<br>Initiate semaglutide vs empagliflozin, sitagliptin, glipizide, or usual care (stable metformin monotherapy).<br><br>**Non-T2D cohort:**<br>Initiate semaglutide vs naltrexone–bupropion, phentermine, phentermine–topiramate, or usual care (no anti-obesity pharmacotherapy).	Treatment groups defined by first qualifying prescription in TriNetX. Eligibility windows: Jan 2018–May 2025 (T2D); Jun 2021–May 2025 (non-T2D), aligned with FDA approval dates.<br><br>Each comparison analyzed as an independent matched cohort. Tirzepatide analyses reported separately in Supplementary Material.
**Treatment assignment**	Random 1:1 allocation at baseline.	Non-random; emulated via 1:1 nearest-neighbor propensity score matching (215 covariates; caliper 0.2 SD of logit PS). Balance confirmed by SMD <0.10.
**Follow-up**	3 years (T2D cohort); 2 years (non-T2D cohort) from treatment initiation to outcome, death, loss to follow-up, or end of study.	Same windows applied within TriNetX. Censoring at last recorded clinical encounter.

**Outcomes**	**Primary:** major osteoporotic fracture (MOF: hip, vertebral, radius/ulna, humerus, fragility).<br>**Secondary:** osteoporosis (M80–M81).<br>**Exploratory:** knee OA, hip OA, gout.<br>**Negative controls:** dog bites, ganglion cysts, adhesive capsulitis, blepharitis, hernias, skin cancer.	Same ICD-10–coded outcomes ascertained after the index date.<br>Surgery code–confirmed hip fracture (CPT 27235/27244/27245; ICD-10-PCS 0QS6/0QS7) as sensitivity endpoint.<br>Composite MOF + all-cause mortality to address competing risks.
**Causal contrasts**	Intention-to-treat (ITT) as primary.<br>Per-protocol (on-treatment) as sensitivity.	**ITT:** follow-up from T_0 regardless of treatment changes.<br>**Per-protocol:** restricted to patients persistent on assigned treatment across 75-day refill intervals (30-day prescription + 45-day gap).
**Statistical analysis**	Cox proportional hazards models; Kaplan–Meier curves; multiple-testing correction.	Cox HRs with 95% CI after 1:1 PSM. Benjamini–Hochberg FDR adjustment. E-values for unmeasured confounding. 3-month landmark, class-level, pooled-comparator, FDA-date–aligned, and global network sensitivity analyses.

Abbreviations: BMI, body mass index; CI, confidence interval; CPT, Current Procedural Terminology; FDA, US Food and Drug Administration; FDR, false discovery rate; HR, hazard ratio; ICD-10, International Classification of Diseases, 10th Revision; ITT, intention-to-treat; MOF, major osteoporotic fracture; OA, osteoarthritis; PS, propensity score; PSM, propensity score matching; SD, standard deviation; SMD, standardized mean difference; T2D, type 2 diabetes.

Supplementary Table 2. Proportions of Missing Data for Key Continuous Covariates Before Propensity Score Matching.

Obesity with type 2 diabetes								
	Semaglutide vs Empagliflozin		Semaglutide vs Sitagliptin		Semaglutide vs Glipizide		Semaglutide vs Usual care	
	Semaglutide missing (%)	Empagliflozin missing (%)	Semaglutide missing (%)	Sitagliptin missing (%)	Semaglutide missing (%)	Glipizide missing (%)	Semaglutide missing (%)	Usual care missing (%)
BMI	9,747 (10.0%)	5,928 (9.5%)	9,747 (10.0%)	1,785 (8.3%)	9,738 (10.0%)	3,337 (8.9%)	9,738 (10.0%)	20,835 (9.1%)
SBP	10,058 (10.4%)	4,684 (7.5%)	10,058 (10.4%)	2,490 (11.6%)	10,058 (10.4%)	3,233 (8.6%)	10,051 (10.4%)	23,079 (10.1%)
eGFR	14,607 (15.1%)	7,164 (11.5%)	14,607 (15.1%)	2,907 (13.6%)	14,607 (15.1%)	4,737 (12.6%)	14,596 (15.0%)	31,180 (13.7%)
TG	24,813 (25.6%)	22,055 (35.4%)	24,813 (25.6%)	7,866 (36.7%)	24,798 (25.6%)	15,608 (41.5%)	24,798 (25.6%)	81,037 (35.5%)
LDL-C	25,632 (26.4%)	22,837 (36.7%)	25,632 (26.4%)	8,127 (37.9%)	25,617 (26.4%)	15,818 (42.0%)	25,617 (26.4%)	83,551 (36.6%)
HbA1c	17,261 (17.8%)	15,951 (25.6%)	17,261 (17.8%)	5,403 (25.2%)	17,261 (17.8%)	10,400 (27.6%)	17,249 (17.8%)	54,725 (24.0%)
Obesity without type 2 diabetes								
	Semaglutide vs Naltrexone/bupropion		Semaglutide vs Phentermine/topiramate		Semaglutide vs Phentermine		Semaglutide vs Usual care	
	Semaglutide missing (%)	Naltrexone/ bupropion missing (%)	Semaglutide missing (%)	Phentermine/ topiramate missing (%)	Semaglutide missing (%)	Phentermine missing (%)	Semaglutide missing (%)	Usual care missing (%)
BMI	10,768 (12.1%)	1,378 (11.5%)	10,767 (12.1%)	1,282 (11.7%)	10,767 (12.1%)	4,034 (10.7%)	10,768 (12.1%)	5,406 (6.5%)
SBP	13,668 (15.4%)	1,252 (10.5%)	13,663 (15.4%)	1,206 (11.0%)	13,654 (15.4%)	3,273 (8.7%)	13,648 (15.4%)	13,224 (16.0%)
eGFR	23,446 (26.4%)	2,539 (21.3%)	23,428 (26.4%)	2,842 (26.0%)	23,412 (26.4%)	10,451 (27.8%)	23,386 (26.3%)	26,833 (32.5%)
TG	32,164 (36.2%)	4,376 (36.7%)	32,144 (36.2%)	4,193 (38.4%)	32,144 (36.2%)	15,551 (41.4%)	32,164 (36.2%)	42,143 (51.0%)
LDL-C	32,743 (36.9%)	4,382 (36.7%)	32,724 (36.8%)	4,172 (38.2%)	32,724 (36.8%)	15,550 (41.4%)	32,743 (36.9%)	42,754 (51.8%)
HbA1c	42,092 (47.4%)	5,630 (47.2%)	42,087 (47.4%)	5,067 (46.4%)	42,067 (47.4%)	20,464 (54.4%)	42,041 (47.3%)	37,543 (45.5%)

Supplementary Table 3. Baseline Characteristics Before and After Propensity Score Matching: Semaglutide Versus Empagliflozin in Obesity With Type 2 Diabetes.

Characteristic	Before Matching			After Matching		
	Semaglutide (n = 97,047)	Empagliflozin (n = 62,244)	SMD	Semaglutide (n = 47,426)	Empagliflozin (n = 47,426)	SMD
**Age**						
Mean ± SD	56.5 ± 13.4	65.1 ± 12.4		61.4 ± 12.3	62.5 ± 12.1	
18-44 years	18,288 (18.8)	3,959 (6.4)	0.383	4,001 (8.4)	3,911 (8.2)	0.007
45-64 years	49,279 (50.8)	23,779 (38.2)	0.255	21,703 (45.8)	21,783 (45.9)	0.003
65-74 years	21,867 (22.5)	19,449 (31.2)	0.197	14,903 (31.4)	14,738 (31.1)	0.008
≥75 years	7,354 (7.6)	15,054 (24.2)	0.467	6,816 (14.4)	6,991 (14.7)	0.010
**Sex (%)**						
Male	39,407 (40.6)	38,812 (62.4)	0.446	27,012 (57.0)	26,568 (56.0)	0.019
Female	57,583 (59.3)	23,396 (37.6)	0.446	20,388 (43.0)	20,829 (43.9)	0.019
**Ethnicity (%)**						
Hispanic or Latino	6,417 (6.6)	4,107 (6.6)	<0.001	3,111 (6.6)	3,074 (6.5)	0.003
Not Hispanic or Latino	70,343 (72.5)	47,674 (76.6)	0.094	35,416 (74.7)	35,590 (75.0)	0.008
**Race (%)**						
White	65,276 (67.3)	42,462 (68.2)	0.020	32,352 (68.2)	32,160 (67.8)	0.009
Black or African American	20,380 (21.0)	12,014 (19.3)	0.042	9,336 (19.7)	9,460 (19.9)	0.007
Other Races	2,447 (2.5)	1,631 (2.6)	0.006	1,279 (2.7)	1,216 (2.6)	0.008
**Socioeconomic determinants**						
Persons with potential health hazards related to socioeconomic and psychosocial circumstances	3,655 (3.8)	3,266 (5.2)	0.071	2,039 (4.3)	2,063 (4.3)	0.002
**Measures of healthcare utilization**						
Visit	96,876 (99.8)	62,104 (99.8)	0.011	47,321 (99.8)	47,317 (99.8)	0.002
Visit: ambulatory	94,449 (97.3)	59,236 (95.2)	0.114	45,580 (96.1)	45,598 (96.1)	0.002
Visit: emergency	26,773 (27.6)	23,588 (37.9)	0.221	15,280 (32.2)	15,514 (32.7)	0.011
**Comorbidities**						
Disorders of lipoprotein metabolism and other lipidemias	66,086 (68.1)	47,310 (76.0)	0.177	34,982 (73.8)	34,972 (73.7)	<0.001
Sleep disorders	27,725 (28.6)	18,138 (29.1)	0.013	13,378 (28.2)	13,687 (28.9)	0.014
Neoplasms	19,574 (20.2)	13,581 (21.8)	0.041	9,846 (20.8)	9,886 (20.8)	0.002
Disorders of thyroid gland	18,183 (18.7)	10,677 (17.2)	0.041	8,021 (16.9)	8,237 (17.4)	0.012
Chronic pain, not elsewhere classified	18,019 (18.6)	10,144 (16.3)	0.060	7,871 (16.6)	8,021 (16.9)	0.008
Depressive episode	15,278 (15.7)	8,362 (13.4)	0.065	6,256 (13.2)	6,411 (13.5)	0.010
Asthma	11,967 (12.3)	5,836 (9.4)	0.095	4,679 (9.9)	4,786 (10.1)	0.008
Diseases of liver	11,638 (12.0)	7,097 (11.4)	0.018	5,301 (11.2)	5,356 (11.3)	0.004
COVID-19	8,585 (8.8)	4,787 (7.7)	0.042	3,592 (7.6)	3,643 (7.7)	0.004

Major depressive disorder, recurrent	7,086 (7.3)	2,799 (4.5)	0.119	2,297 (4.8)	2,411 (5.1)	0.011
Sleep disorders not due to a substance or known physiological condition	3,541 (3.6)	1,419 (2.3)	0.081	1,233 (2.6)	1,218 (2.6)	0.002
Other disorders of bone density and structure	3,616 (3.7)	2,273 (3.7)	0.004	1,760 (3.7)	1,813 (3.8)	0.006
Other symptoms and signs involving cognitive functions and awareness	3,626 (3.7)	4,199 (6.7)	0.135	2,306 (4.9)	2,362 (5.0)	0.005
Slipping, tripping, stumbling and falls	4,014 (4.1)	4,251 (6.8)	0.119	2,402 (5.1)	2,465 (5.2)	0.006
Extrapyramidal and movement disorders	3,460 (3.6)	2,593 (4.2)	0.031	1,750 (3.7)	1,837 (3.9)	0.010
Bipolar disorder	2,622 (2.7)	1,332 (2.1)	0.037	1,059 (2.2)	1,092 (2.3)	0.005
Psoriasis	2,122 (2.2)	1,008 (1.6)	0.042	825 (1.7)	835 (1.8)	0.002
Age-related cataract	4,496 (4.6)	3,127 (5.0)	0.018	2,399 (5.1)	2,417 (5.1)	0.002
Somnolence, stupor and coma	1,414 (1.5)	920 (1.5)	0.002	591 (1.2)	631 (1.3)	0.007
Certain disorders involving the immune mechanism	1,631 (1.7)	1,392 (2.2)	0.040	919 (1.9)	938 (2.0)	0.003
Glaucoma	2,637 (2.7)	2,122 (3.4)	0.040	1,475 (3.1)	1,497 (3.2)	0.003
History of falling	1,605 (1.7)	1,924 (3.1)	0.095	1,067 (2.2)	1,105 (2.3)	0.005
Personal history of (healed) traumatic fracture	367 (0.4)	283 (0.5)	0.012	185 (0.4)	182 (0.4)	0.001
Repeated falls	791 (0.8)	1,308 (2.1)	0.107	595 (1.3)	615 (1.3)	0.004
Long term (current) use of systemic steroids	855 (0.9)	995 (1.6)	0.065	535 (1.1)	557 (1.2)	0.004
**Procedure**						
Encounter for screening for osteoporosis	2,312 (2.4)	1,132 (1.8)	0.039	984 (2.1)	1,014 (2.1)	0.004
Dual-energy x-ray absorptiometry (dxa), bone density study, 1 or more sites	4,071 (4.2)	2,026 (3.3)	0.050	1,723 (3.6)	1,788 (3.8)	0.007
**Medications**						
Glucocorticoids	42,214 (43.5)	28,174 (45.3)	0.036	20,397 (43.0)	20,618 (43.5)	0.009
Antihistamines for systemic use	24,847 (25.6)	17,243 (27.7)	0.047	12,230 (25.8)	12,483 (26.3)	0.012
Selective serotonin reuptake inhibitors	15,881 (16.4)	7,964 (12.8)	0.101	6,105 (12.9)	6,299 (13.3)	0.012
Antiepileptics	20,626 (21.3)	14,949 (24.0)	0.066	10,478 (22.1)	10,708 (22.6)	0.012
Other antidepressants	16,896 (17.4)	10,161 (16.3)	0.029	7,332 (15.5)	7,533 (15.9)	0.012
Vitamin D and analogues	12,525 (12.9)	11,154 (17.9)	0.139	7,013 (14.8)	7,179 (15.1)	0.010
Thyroid therapy	11,065 (11.4)	6,728 (10.8)	0.019	5,035 (10.6)	5,111 (10.8)	0.005

Chemotherapeutics for topical use	8,266 (8.5)	5,604 (9.0)	0.017	3,920 (8.3)	3,959 (8.3)	0.003
Antipsychotics	10,148 (10.5)	8,635 (13.9)	0.105	5,564 (11.7)	5,676 (12.0)	0.007
Metformin	47,034 (48.5)	23,688 (38.1)	0.211	20,282 (42.8)	20,055 (42.3)	0.010
Antiparasitic products, insecticides and repellents	6,456 (6.7)	3,709 (6.0)	0.029	2,637 (5.6)	2,722 (5.7)	0.008
COVID-19 vaccine	16,298 (16.8)	9,354 (15.0)	0.048	7,432 (15.7)	7,411 (15.6)	0.001
Antineoplastic agents	5,932 (6.1)	3,978 (6.4)	0.012	2,835 (6.0)	2,901 (6.1)	0.006
Antimigraine preparations	4,777 (4.9)	2,346 (3.8)	0.057	1,785 (3.8)	1,860 (3.9)	0.008
Immunosuppressants	2,726 (2.8)	1,414 (2.3)	0.034	1,099 (2.3)	1,139 (2.4)	0.006
Drugs used in addictive disorders	4,315 (4.4)	3,884 (6.2)	0.080	2,553 (5.4)	2,600 (5.5)	0.004
Non-selective monoamine reuptake inhibitors	2,917 (3.0)	1,629 (2.6)	0.024	1,261 (2.7)	1,278 (2.7)	0.002
Anti-Parkinson drugs	2,900 (3.0)	2,362 (3.8)	0.045	1,576 (3.3)	1,641 (3.5)	0.008
Aromatase inhibitors	824 (0.8)	422 (0.7)	0.020	336 (0.7)	377 (0.8)	0.010
Cardiovascular agents,other	1,181 (1.2)	2,445 (3.9)	0.172	990 (2.1)	993 (2.1)	<0.001
Bisphosphonates	700 (0.7)	477 (0.8)	0.005	349 (0.7)	379 (0.8)	0.007
Alendronate	533 (0.5)	354 (0.6)	0.003	267 (0.6)	287 (0.6)	0.006
Other drugs affecting bone structure and mineralization	171 (0.2)	132 (0.2)	0.008	83 (0.2)	95 (0.2)	0.006
Denosumab	153 (0.2)	119 (0.2)	0.008	75 (0.2)	87 (0.2)	0.006
Zoledronic acid	130 (0.1)	94 (0.2)	0.005	61 (0.1)	69 (0.1)	0.005
Ibandronate	48 (0.0)	29 (0.0)	0.001	25 (0.1)	23 (0.0)	0.002
Romosozumab	≤10 (0.0)	≤10 (0.0)	0.005	≤10 (0.0)	≤10 (0.0)	<0.001
Risedronic acid	≤10 (0.0)	N/A	N/A	N/A	N/A	N/A
Teriparatide	12 (0.0)	≤10 (0.0)	0.003	≤10 (0.0)	≤10 (0.0)	<0.001
Abaloparatide	≤10 (0.0)	≤10 (0.0)	0.005	≤10 (0.0)	≤10 (0.0)	<0.001
**Laboratory**						
**BMI**						
Mean ± SD, kg/m^2	37.2 ± 7.4	33.4 ± 6.4		36.0 ± 7.0	34.1 ± 6.5	
≥30 kg/m^2	79,404 (81.8)	44,469 (71.4)	0.247	36,001 (75.9)	36,016 (75.9)	<0.001
**SBP**						
Mean ± SD, mm[hg]	130.2 ± 17.1	128.3 ± 20.9		131.2 ± 17.6	128.5 ± 20.5	
≥140 mm[hg]	58,403 (60.2)	41,520 (66.7)	0.136	30,457 (64.2)	30,568 (64.5)	0.005
**eGFR**						
Mean ± SD, ml/min/(1.73_m^2)	83.6 ± 25.7	72.8 ± 28.1		79.6 ± 25.6	75.8 ± 28.1	
≥60 ml/min/(1.73_m^2)	75,549 (77.8)	44,262 (71.1)	0.155	34,967 (73.7)	35,032 (73.9)	0.003
**TG**						
Mean ± SD, mg/dl	169.4 ± 139.6	166.7 ± 168.2		164.8 ± 136.3	175.1 ± 183.6	
≥200 mg/dl	24,247 (25.0)	12,585 (20.2)	0.114	10,872 (22.9)	10,734 (22.6)	0.007
**LDL-C**						
Mean ± SD, mg/dl	94.1 ± 37.3	83.2 ± 37.3		89.2 ± 36.8	85.7 ± 37.8	
≥190 mg/dl	1,844 (1.9)	812 (1.3)	0.047	714 (1.5)	717 (1.5)	<0.001

**HbA1C**						
Mean ± SD, %	7.3 ± 1.8	7.5 ± 1.7		7.4 ± 1.8	7.5 ± 1.8	
≥7%	44,094 (45.4)	29,226 (47.0)	0.030	22,989 (48.5)	22,664 (47.8)	0.014

Data are presented as mean ± SD for continuous variables and n (%) for categorical variables. SMD, standardized mean difference. A post-matching SMD <0.10 indicates adequate balance. Matching was performed using 1:1 nearest-neighbor propensity score matching with a caliper of 0.2 SD of the logit propensity score based on 215 covariates. SMD, standardized mean difference; COVID-19, coronavirus disease 2019; BMI, body mass index; SBP, systolic blood pressure; eGFR, estimated glomerular filtration rate; TG, triglycerides; LDL-C, low-density lipoprotein cholesterol; HbA1c, glycated hemoglobin A1c.

Supplementary Table 4. Baseline Characteristics Before and After Propensity Score Matching: Semaglutide Versus Sitagliptin in Obesity With Type 2 Diabetes.

	Before Matching			After Matching		
Characteristic	Semaglutide (n = 97,047)	Sitagliptin (n = 21,448)	SMD	Semaglutide (n = 19,824)	Sitagliptin (n = 19,824)	SMD
**Age**						
Mean ± SD	56.5 ± 13.4	66.3 ± 13.1		64.1 ± 12.7	65.4 ± 13.0	
18-44 years	18,288 (18.8)	1,356 (6.3)	0.384	1,295 (6.5)	1,355 (6.8)	0.012
45-64 years	49,279 (50.8)	7,408 (34.5)	0.333	7,356 (37.1)	7,312 (36.9)	0.005
65-74 years	21,867 (22.5)	6,419 (29.9)	0.169	6,136 (31.0)	6,144 (31.0)	<0.001
≥75 years	7,354 (7.6)	6,264 (29.2)	0.581	5,036 (25.4)	5,012 (25.3)	0.003
**Sex (%)**						
Male	39,407 (40.6)	10,265 (47.9)	0.146	9,489 (47.9)	9,404 (47.4)	0.009
Female	57,583 (59.3)	11,169 (52.1)	0.147	10,324 (52.1)	10,407 (52.5)	0.008
**Ethnicity (%)**						
Hispanic or Latino	6,417 (6.6)	1,633 (7.6)	0.039	1,471 (7.4)	1,468 (7.4)	<0.001
Not Hispanic or Latino	70,343 (72.5)	15,412 (71.9)	0.014	14,494 (73.1)	14,325 (72.3)	0.019
**Race (%)**						
White	65,276 (67.3)	12,855 (59.9)	0.153	12,116 (61.1)	12,127 (61.2)	0.001
Black or African American	20,380 (21.0)	4,867 (22.7)	0.041	4,491 (22.7)	4,480 (22.6)	0.001
Other Races	2,447 (2.5)	933 (4.3)	0.101	709 (3.6)	762 (3.8)	0.014
**Socioeconomic determinants**						
Persons with potential health hazards related to socioeconomic and psychosocial circumstances	3,655 (3.8)	989 (4.6)	0.042	829 (4.2)	877 (4.4)	0.012
**Measures of healthcare utilization**						
Visit	96,876 (99.8)	21,395 (99.8)	0.015	19,778 (99.8)	19,776 (99.8)	0.002
Visit: ambulatory	94,449 (97.3)	18,769 (87.5)	0.377	18,087 (91.2)	17,988 (90.7)	0.017
Visit: emergency	26,773 (27.6)	6,695 (31.2)	0.08	6,131 (30.9)	6,056 (30.5)	0.008
**Comorbidities**						
Disorders of lipoprotein metabolism and other lipidemias	66,086 (68.1)	15,828 (73.8)	0.126	14,540 (73.3)	14,599 (73.6)	0.007
Sleep disorders	27,725 (28.6)	4,338 (20.2)	0.195	4,143 (20.9)	4,177 (21.1)	0.004
Neoplasms	19,574 (20.2)	4,907 (22.9)	0.066	4,530 (22.9)	4,532 (22.9)	<0.001
Disorders of thyroid gland	18,183 (18.7)	4,185 (19.5)	0.020	3,725 (18.8)	3,833 (19.3)	0.014
Chronic pain, not elsewhere classified	18,019 (18.6)	3,270 (15.2)	0.089	3,145 (15.9)	3,122 (15.7)	0.003
Depressive episode	15,278 (15.7)	2,769 (12.9)	0.081	2,503 (12.6)	2,568 (13.0)	0.010
Asthma	11,967 (12.3)	2,064 (9.6)	0.087	1,926 (9.7)	1,964 (9.9)	0.006
Diseases of liver	11,638 (12.0)	2,105 (9.8)	0.07	1,959 (9.9)	1,992 (10.0)	0.006
COVID-19	8,585 (8.8)	1,680 (7.8)	0.037	1,562 (7.9)	1,554 (7.8)	0.001

Major depressive disorder, recurrent	7,086 (7.3)	1,054 (4.9)	0.100	1,029 (5.2)	1,007 (5.1)	0.005
Sleep disorders not due to a substance or known physiological condition	3,541 (3.6)	535 (2.5)	0.067	494 (2.5)	512 (2.6)	0.006
Other disorders of bone density and structure	3,616 (3.7)	949 (4.4)	0.035	896 (4.5)	887 (4.5)	0.002
Other symptoms and signs involving cognitive functions and awareness	3,626 (3.7)	1,730 (8.1)	0.185	1,304 (6.6)	1,372 (6.9)	0.014
Slipping, tripping, stumbling and falls	4,014 (4.1)	1,588 (7.4)	0.140	1,286 (6.5)	1,312 (6.6)	0.005
Extrapyramidal and movement disorders	3,460 (3.6)	875 (4.1)	0.027	781 (3.9)	794 (4.0)	0.003
Bipolar disorder	2,622 (2.7)	564 (2.6)	0.004	490 (2.5)	520 (2.6)	0.010
Psoriasis	2,122 (2.2)	331 (1.5)	0.048	312 (1.6)	320 (1.6)	0.003
Age-related cataract	4,496 (4.6)	1,138 (5.3)	0.031	1,107 (5.6)	1,095 (5.5)	0.003
Somnolence, stupor and coma	1,414 (1.5)	307 (1.4)	0.002	257 (1.3)	270 (1.4)	0.006
Certain disorders involving the immune mechanism	1,631 (1.7)	411 (1.9)	0.018	383 (1.9)	375 (1.9)	0.003
Glaucoma	2,637 (2.7)	854 (4.0)	0.070	778 (3.9)	759 (3.8)	0.005
History of falling	1,605 (1.7)	801 (3.7)	0.129	647 (3.3)	649 (3.3)	<0.001
Personal history of (healed) traumatic fracture	367 (0.4)	88 (0.4)	0.005	67 (0.3)	85 (0.4)	0.015
Repeated falls	791 (0.8)	497 (2.3)	0.121	351 (1.8)	377 (1.9)	0.010
Long term (current) use of systemic steroids	855 (0.9)	314 (1.5)	0.054	276 (1.4)	268 (1.4)	0.003
**Procedure**						
Encounter for screening for osteoporosis	2,312 (2.4)	503 (2.3)	0.002	463 (2.3)	488 (2.5)	0.008
Dual-energy x-ray absorptiometry (dxa), bone density study, 1 or more sites	4,071 (4.2)	886 (4.1)	0.003	826 (4.2)	849 (4.3)	0.006
**Medications**						
Glucocorticoids	42,214 (43.5)	8,615 (40.2)	0.068	8,040 (40.6)	8,029 (40.5)	0.001
Antihistamines for systemic use	24,847 (25.6)	5,771 (26.9)	0.030	5,315 (26.8)	5,291 (26.7)	0.003
Selective serotonin reuptake inhibitors	15,881 (16.4)	2,803 (13.1)	0.093	2,598 (13.1)	2,601 (13.1)	<0.001
Antiepileptics	20,626 (21.3)	4,970 (23.2)	0.046	4,503 (22.7)	4,504 (22.7)	<0.001
Other antidepressants	16,896 (17.4)	3,121 (14.6)	0.078	2,804 (14.1)	2,877 (14.5)	0.011
Vitamin D and analogues	12,525 (12.9)	3,459 (16.1)	0.092	3,053 (15.4)	3,129 (15.8)	0.011
Thyroid therapy	11,065 (11.4)	2,691 (12.5)	0.035	2,420 (12.2)	2,451 (12.4)	0.005

Chemotherapeutics for topical use	8,266 (8.5)	1,741 (8.1)	0.014	1,611 (8.1)	1,597 (8.1)	0.003
Antipsychotics	10,148 (10.5)	3,088 (14.4)	0.120	2,654 (13.4)	2,682 (13.5)	0.004
Metformin	47,034 (48.5)	10,347 (48.2)	0.004	9,958 (50.2)	9,645 (48.7)	0.032
Antiparasitic products, insecticides and repellents	6,456 (6.7)	1,296 (6.0)	0.025	1,236 (6.2)	1,200 (6.1)	0.008
COVID-19 vaccine	16,298 (16.8)	3,505 (16.3)	0.012	3,413 (17.2)	3,334 (16.8)	0.011
Antineoplastic agents	5,932 (6.1)	1,371 (6.4)	0.012	1,282 (6.5)	1,271 (6.4)	0.002
Antimigraine preparations	4,777 (4.9)	823 (3.8)	0.053	736 (3.7)	754 (3.8)	0.005
Immunosuppressants	2,726 (2.8)	535 (2.5)	0.020	478 (2.4)	502 (2.5)	0.008
Drugs used in addictive disorders	4,315 (4.4)	887 (4.1)	0.015	816 (4.1)	843 (4.3)	0.007
Non-selective monoamine reuptake inhibitors	2,917 (3.0)	531 (2.5)	0.032	527 (2.7)	493 (2.5)	0.011
Anti-Parkinson drugs	2,900 (3.0)	775 (3.6)	0.035	678 (3.4)	704 (3.6)	0.007
Aromatase inhibitors	824 (0.8)	210 (1.0)	0.014	190 (1.0)	194 (1.0)	0.002
Cardiovascular agents,other	1,181 (1.2)	481 (2.2)	0.079	402 (2.0)	378 (1.9)	0.009
Bisphosphonates	700 (0.7)	255 (1.2)	0.048	246 (1.2)	226 (1.1)	0.009
Alendronate	533 (0.5)	191 (0.9)	0.040	183 (0.9)	168 (0.8)	0.008
Other drugs affecting bone structure and mineralization	171 (0.2)	66 (0.3)	0.027	55 (0.3)	55 (0.3)	<0.001
Denosumab	153 (0.2)	62 (0.3)	0.028	52 (0.3)	51 (0.3)	<0.001
Zoledronic acid	130 (0.1)	46 (0.2)	0.019	49 (0.2)	42 (0.2)	0.007
Ibandronate	48 (0.0)	18 (0.1)	0.013	15 (0.1)	17 (0.1)	0.004
Romosozumab	≤10 (0.0)	≤10 (0.0)	0.022	≤10 (0.1)	≤10 (0.1)	<0.001
Risedronic acid	≤10 (0.0)	N/A	N/A	N/A	N/A	N/A
Teriparatide	12 (0.0)	≤10 (0.0)	0.020	N/A	≤10 (0.1)	N/A
Abaloparatide	≤10 (0.0)	≤10 (0.0)	0.022	≤10 (0.1)	≤10 (0.1)	<0.001
**Laboratory**						
**BMI**						
Mean ± SD, kg/m^2	37.2 ± 7.4	32.9 ± 6.0		35.2 ± 6.9	33.1 ± 6.0	
≥30 kg/m^2	79,404 (81.8)	14,577 (68.0)	0.324	13,943 (70.3)	13,942 (70.3)	<0.001
**SBP**						
Mean ± SD, mm[hg]	130.2 ± 17.1	131.7 ± 18.6		131.3 ± 17.7	131.6 ± 18.5	
≥140 mm[hg]	58,403 (60.2)	13,354 (62.3)	0.043	12,089 (61.0)	12,356 (62.3)	0.028
**eGFR**						
Mean ± SD, ml/min/(1.73_m^2)	83.6 ± 25.7	74.3 ± 28.0		77.1 ± 25.9	75.2 ± 27.9	
≥60 ml/min/(1.73_m^2)	75,549 (77.8)	15,187 (70.8)	0.162	14,157 (71.4)	14,191 (71.6)	0.004
**TG**						
Mean ± SD, mg/dl	169.4 ± 139.6	161.8 ± 128.1		159.3 ± 138.4	163.6 ± 130.1	
≥200 mg/dl	24,247 (25.0)	4,153 (19.4)	0.136	4,041 (20.4)	4,014 (20.2)	0.003
**LDL-C**						
Mean ± SD, mg/dl	94.1 ± 37.3	87.9 ± 37.9		87.5 ± 36.2	88.5 ± 37.9	
≥190 mg/dl	1,844 (1.9)	320 (1.5)	0.032	284 (1.4)	306 (1.5)	0.009

**HbA1C**						
Mean ± SD, %	7.3 ± 1.8	7.7 ± 1.8		7.5 ± 1.8	7.7 ± 1.8	
≥7%	44,094 (45.4)	11,074 (51.6)	0.124	10,387 (52.4)	10,202 (51.5)	0.019

Data are presented as mean ± SD for continuous variables and n (%) for categorical variables. SMD, standardized mean difference. A post-matching SMD <0.10 indicates adequate balance. Matching was performed using 1:1 nearest-neighbor propensity score matching with a caliper of 0.2 SD of the logit propensity score based on 215 covariates. SMD, standardized mean difference; COVID-19, coronavirus disease 2019; BMI, body mass index; SBP, systolic blood pressure; eGFR, estimated glomerular filtration rate; TG, triglycerides; LDL-C, low-density lipoprotein cholesterol; HbA1c, glycated hemoglobin A1c.

Supplementary Table 5. Baseline Characteristics Before and After Propensity Score Matching: Semaglutide Versus Glipizide in Obesity With Type 2 Diabetes.

	Before Matching			After Matching		
Characteristic	Semaglutide (n = 97,047)	Glipizide (n = 37,635)	SMD	Semaglutide (n = 32,594)	Glipizide (n = 32,594)	SMD
**Age**						
Mean ± SD	56.5 ± 13.4	64.6 ± 13.5		62.2 ± 13.2	63.2 ± 13.3	
18-44 years	18,288 (18.8)	3,325 (8.8)	0.293	3,191 (9.8)	3,218 (9.9)	0.003
45-64 years	49,279 (50.8)	13,733 (36.5)	0.291	12,749 (39.1)	12,961 (39.8)	0.013
65-74 years	21,867 (22.5)	11,101 (29.5)	0.159	10,351 (31.8)	9,977 (30.6)	0.025
≥75 years	7,354 (7.6)	9,473 (25.2)	0.489	6,299 (19.3)	6,435 (19.7)	0.011
**Sex (%)**						
Male	39,407 (40.6)	20,640 (54.8)	0.288	17,383 (53.3)	17,089 (52.4)	0.018
Female	57,583 (59.3)	16,971 (45.1)	0.288	15,186 (46.6)	15,486 (47.5)	0.018
**Ethnicity (%)**						
Hispanic or Latino	6,417 (6.6)	3,061 (8.1)	0.058	2,580 (7.9)	2,526 (7.8)	0.006
Not Hispanic or Latino	70,343 (72.5)	25,617 (68.1)	0.097	22,490 (69.0)	22,511 (69.1)	0.001
**Race (%)**						
White	65,276 (67.3)	24,852 (66.0)	0.026	21,522 (66.0)	21,634 (66.4)	0.007
Black or African American	20,380 (21.0)	7,326 (19.5)	0.038	6,499 (19.9)	6,466 (19.8)	0.003
Other Races	2,447 (2.5)	1,655 (4.4)	0.103	1,222 (3.7)	1,203 (3.7)	0.003
**Socioeconomic determinants**						
Persons with potential health hazards related to socioeconomic and psychosocial circumstances	3,661 (3.8)	1,807 (4.8)	0.051	1,411 (4.3)	1,437 (4.4)	0.004
**Measures of healthcare utilization**						
Visit	96,877 (99.8)	37,506 (99.7)	0.033	32,477 (99.6)	32,491 (99.7)	0.007
Visit: ambulatory	94,454 (97.3)	32,821 (87.2)	0.386	30,255 (92.8)	30,051 (92.2)	0.024
Visit: emergency	26,787 (27.6)	12,424 (33.0)	0.118	10,396 (31.9)	10,312 (31.6)	0.006
**Comorbidities**						
Disorders of lipoprotein metabolism and other lipidemias	66,105 (68.1)	25,944 (68.9)	0.018	22,635 (69.4)	22,579 (69.3)	0.004
Sleep disorders	27,743 (28.6)	7,239 (19.2)	0.221	6,708 (20.6)	6,758 (20.7)	0.004
Neoplasms	19,585 (20.2)	7,796 (20.7)	0.013	6,741 (20.7)	6,744 (20.7)	<0.001
Disorders of thyroid gland	18,190 (18.7)	6,123 (16.3)	0.065	5,429 (16.7)	5,440 (16.7)	<0.001
Chronic pain, not elsewhere classified	18,031 (18.6)	5,496 (14.6)	0.107	5,075 (15.6)	4,985 (15.3)	0.008
Depressive episode	15,284 (15.7)	4,494 (11.9)	0.110	3,989 (12.2)	4,050 (12.4)	0.006
Asthma	11,973 (12.3)	3,155 (8.4)	0.130	2,848 (8.7)	2,910 (8.9)	0.007
Diseases of liver	11,645 (12.0)	3,458 (9.2)	0.091	3,089 (9.5)	3,137 (9.6)	0.005
COVID-19	8,593 (8.9)	2,804 (7.5)	0.051	2,417 (7.4)	2,442 (7.5)	0.003

Major depressive disorder, recurrent	7,092 (7.3)	1,604 (4.3)	0.131	1,482 (4.5)	1,509 (4.6)	0.004
Sleep disorders not due to a substance or known physiological condition	3,543 (3.7)	829 (2.2)	0.086	705 (2.2)	769 (2.4)	0.013
Other disorders of bone density and structure	3,625 (3.7)	1,197 (3.2)	0.030	1,078 (3.3)	1,101 (3.4)	0.004
Other symptoms and signs involving cognitive functions and awareness	3,629 (3.7)	2,359 (6.3)	0.116	1,695 (5.2)	1,733 (5.3)	0.005
Slipping, tripping, stumbling and falls	4,016 (4.1)	2,441 (6.5)	0.105	1,817 (5.6)	1,818 (5.6)	<0.001
Extrapyramidal and movement disorders	3,462 (3.6)	1,372 (3.6)	0.004	1,203 (3.7)	1,151 (3.5)	0.009
Bipolar disorder	2,622 (2.7)	964 (2.6)	0.009	844 (2.6)	846 (2.6)	<0.001
Psoriasis	2,122 (2.2)	536 (1.4)	0.057	481 (1.5)	497 (1.5)	0.004
Age-related cataract	4,500 (4.6)	1,865 (5.0)	0.015	1,723 (5.3)	1,692 (5.2)	0.004
Somnolence, stupor and coma	1,416 (1.5)	462 (1.2)	0.020	342 (1.0)	377 (1.2)	0.010
Certain disorders involving the immune mechanism	1,632 (1.7)	621 (1.6)	0.002	538 (1.7)	545 (1.7)	0.002
Glaucoma	2,640 (2.7)	1,265 (3.4)	0.037	1,100 (3.4)	1,075 (3.3)	0.004
History of falling	1,608 (1.7)	1,118 (3.0)	0.087	828 (2.5)	831 (2.5)	<0.001
Personal history of (healed) traumatic fracture	368 (0.4)	127 (0.3)	0.007	113 (0.3)	112 (0.3)	<0.001
Repeated falls	792 (0.8)	671 (1.8)	0.085	469 (1.4)	458 (1.4)	0.003
Long term (current) use of systemic steroids	856 (0.9)	488 (1.3)	0.040	367 (1.1)	381 (1.2)	0.004
**Procedure**						
Encounter for screening for osteoporosis	2,316 (2.4)	699 (1.9)	0.037	667 (2.0)	675 (2.1)	0.002
Dual-energy x-ray absorptiometry (dxa), bone density study, 1 or more sites	4,078 (4.2)	1,077 (2.9)	0.073	1,008 (3.1)	1,022 (3.1)	0.002
**Medications**						
Glucocorticoids	42,236 (43.5)	14,321 (38.1)	0.111	12,679 (38.9)	12,742 (39.1)	0.004
Antihistamines for systemic use	24,871 (25.6)	8,962 (23.8)	0.042	7,853 (24.1)	7,872 (24.2)	0.001
Selective serotonin reuptake inhibitors	15,890 (16.4)	4,491 (11.9)	0.128	3,965 (12.2)	4,057 (12.4)	0.009
Antiepileptics	20,644 (21.3)	8,279 (22.0)	0.018	7,117 (21.8)	7,065 (21.7)	0.004
Other antidepressants	16,911 (17.4)	4,944 (13.1)	0.119	4,347 (13.3)	4,426 (13.6)	0.007
Vitamin D and analogues	12,532 (12.9)	4,729 (12.6)	0.010	4,082 (12.5)	4,074 (12.5)	<0.001
Thyroid therapy	11,068 (11.4)	3,853 (10.2)	0.038	3,383 (10.4)	3,363 (10.3)	0.002

Chemotherapeutics for topical use	8,278 (8.5)	2,876 (7.6)	0.033	2,532 (7.8)	2,526 (7.8)	<0.001
Antipsychotics	10,153 (10.5)	5,481 (14.6)	0.124	4,346 (13.3)	4,356 (13.4)	<0.001
Metformin	47,053 (48.5)	17,132 (45.5)	0.059	15,436 (47.4)	15,182 (46.6)	0.016
Antiparasitic products, insecticides and repellents	6,464 (6.7)	2,185 (5.8)	0.035	1,879 (5.8)	1,899 (5.8)	0.003
COVID-19 vaccine	16,317 (16.8)	5,480 (14.6)	0.062	5,002 (15.3)	4,953 (15.2)	0.004
Antineoplastic agents	5,940 (6.1)	2,133 (5.7)	0.019	1,747 (5.4)	1,871 (5.7)	0.017
Antimigraine preparations	4,782 (4.9)	1,374 (3.7)	0.063	1,200 (3.7)	1,221 (3.7)	0.003
Immunosuppressants	2,729 (2.8)	651 (1.7)	0.073	561 (1.7)	605 (1.9)	0.010
Drugs used in addictive disorders	4,318 (4.4)	1,856 (4.9)	0.023	1,521 (4.7)	1,573 (4.8)	0.008
Non-selective monoamine reuptake inhibitors	2,920 (3.0)	843 (2.2)	0.048	762 (2.3)	770 (2.4)	0.002
Anti-Parkinson drugs	2,902 (3.0)	1,269 (3.4)	0.022	1,086 (3.3)	1,063 (3.3)	0.004
Aromatase inhibitors	824 (0.8)	244 (0.6)	0.023	226 (0.7)	222 (0.7)	0.001
Cardiovascular agents,other	1,182 (1.2)	667 (1.8)	0.046	513 (1.6)	539 (1.7)	0.006
Bisphosphonates	701 (0.7)	286 (0.8)	0.004	242 (0.7)	242 (0.7)	<0.001
Alendronate	533 (0.5)	205 (0.5)	<0.001	176 (0.5)	174 (0.5)	<0.001
Other drugs affecting bone structure and mineralization	171 (0.2)	56 (0.1)	0.007	44 (0.1)	50 (0.2)	0.005
Denosumab	153 (0.2)	53 (0.1)	0.004	41 (0.1)	47 (0.1)	0.005
Zoledronic acid	131 (0.1)	64 (0.2)	0.009	49 (0.1)	54 (0.2)	0.004
Ibandronate	48 (0.0)	16 (0.0)	0.003	17 (0.1)	15 (0.0)	0.003
Romosozumab	≤10 (0.0)	N/A	N/A	N/A	N/A	N/A
Risedronic acid	≤10 (0.0)	≤10 (0.0)	0.012	≤10 (0.0)	≤10 (0.0)	<0.001
Teriparatide	12 (0.0)	N/A	N/A	N/A	N/A	N/A
Abaloparatide	≤10 (0.0)	≤10 (0.0)	0.012	≤10 (0.0)	≤10 (0.0)	<0.001
**Laboratory**						
**BMI**						
Mean ± SD, kg/m^2	37.2 ± 7.4	33.5 ± 6.2		35.6 ± 6.9	33.9 ± 6.3	
≥30 kg/m^2	79,415 (81.8)	26,677 (70.9)	0.260	24,094 (73.9)	24,072 (73.9)	0.002
**SBP**						
Mean ± SD, mm[hg]	130.2 ± 17.1	132.6 ± 19.7		131.7 ± 17.7	132.3 ± 19.5	
≥140 mm[hg]	58,403 (60.2)	24,569 (65.3)	0.106	20,943 (64.3)	21,052 (64.6)	0.007
**eGFR**						
Mean ± SD, ml/min/(1.73_m^2)	83.6 ± 25.7	76.8 ± 30.1		79.2 ± 26.1	78.2 ± 29.9	
≥60 ml/min/(1.73_m^2)	75,549 (77.8)	26,900 (71.5)	0.147	23,614 (72.4)	23,740 (72.8)	0.009
**TG**						
Mean ± SD, mg/dl	169.4 ± 139.6	176.5 ± 157.0		162.6 ± 133.5	180.2 ± 162.4	
≥200 mg/dl	24,263 (25.0)	7,425 (19.7)	0.127	7,087 (21.7)	6,879 (21.1)	0.016
**LDL-C**						
Mean ± SD, mg/dl	94.1 ± 37.3	88.9 ± 38.4		88.4 ± 36.5	89.9 ± 38.3	
≥190 mg/dl	1,844 (1.9)	515 (1.4)	0.042	462 (1.4)	485 (1.5)	0.006

**HbA1C**						
Mean ± SD, %	7.3 ± 1.8	8.0 ± 1.9		7.6 ± 1.8	8.0 ± 1.9	
≥7%	44,094 (45.4)	20,679 (54.9)	0.191	18,035 (55.3)	17,723 (54.4)	0.019

Data are presented as mean ± SD for continuous variables and n (%) for categorical variables. SMD, standardized mean difference. A post-matching SMD <0.10 indicates adequate balance. Matching was performed using 1:1 nearest-neighbor propensity score matching with a caliper of 0.2 SD of the logit propensity score based on 215 covariates. SMD, standardized mean difference; COVID-19, coronavirus disease 2019; BMI, body mass index; SBP, systolic blood pressure; eGFR, estimated glomerular filtration rate; TG, triglycerides; LDL-C, low-density lipoprotein cholesterol; HbA1c, glycated hemoglobin A1c.

Supplementary Table 6. Baseline Characteristics Before and After Propensity Score Matching: Semaglutide Versus Usual Care in Obesity With Type 2 Diabetes.

	Before Matching			After Matching		
Characteristic	Semaglutide (n = 97,047)	Usual care (n = 228,192)	SMD	Semaglutide (n = 93,519)	Usual care (n = 93,519)	SMD
**Age**						
Mean ± SD	56.5 ± 13.4	60.5 ± 14.6		56.7 ± 13.5	57.2 ± 13.7	
18-44 years	18,288 (18.8)	33,798 (14.8)	0.108	17,350 (18.6)	17,170 (18.4)	0.005
45-64 years	49,279 (50.8)	92,812 (40.7)	0.204	46,871 (50.1)	47,309 (50.6)	0.009
65-74 years	21,867 (22.5)	62,310 (27.3)	0.111	21,690 (23.2)	21,488 (23.0)	0.005
≥75 years	7,354 (7.6)	38,504 (16.9)	0.287	7,349 (7.9)	7,273 (7.8)	0.003
**Sex (%)**						
Male	39,407 (40.6)	118,126 (51.8)	0.225	39,077 (41.8)	39,632 (42.4)	0.012
Female	57,583 (59.3)	109,932 (48.2)	0.225	54,394 (58.2)	53,841 (57.6)	0.012
**Ethnicity (%)**						
Hispanic or Latino	6,417 (6.6)	22,107 (9.7)	0.113	6,373 (6.8)	6,233 (6.7)	0.006
Not Hispanic or Latino	70,343 (72.5)	158,477 (69.4)	0.067	67,864 (72.6)	68,278 (73.0)	0.010
**Race (%)**						
White	65,276 (67.3)	141,118 (61.8)	0.113	62,383 (66.7)	62,790 (67.1)	0.009
Black or African American	20,380 (21.0)	46,644 (20.4)	0.014	19,788 (21.2)	19,630 (21.0)	0.004
Other Races	2,447 (2.5)	11,309 (5.0)	0.129	2,441 (2.6)	2,376 (2.5)	0.004
**Socioeconomic determinants**						
Persons with potential health hazards related to socioeconomic and psychosocial circumstances	3,661 (3.8)	11,511 (5.0)	0.062	3,606 (3.9)	3,471 (3.7)	0.008
**Measures of healthcare utilization**						
Visit	96,877 (99.8)	227,610 (99.7)	0.017	93,349 (99.8)	93,344 (99.8)	0.001
Visit: ambulatory	94,454 (97.3)	200,308 (87.8)	0.370	90,926 (97.2)	90,612 (96.9)	0.020
Visit: emergency	26,787 (27.6)	66,948 (29.3)	0.038	25,990 (27.8)	24,970 (26.7)	0.024
**Comorbidities**						
Disorders of lipoprotein metabolism and other lipidemias	66,105 (68.1)	151,228 (66.3)	0.039	63,354 (67.7)	63,236 (67.6)	0.003
Sleep disorders	27,743 (28.6)	48,235 (21.1)	0.173	25,672 (27.5)	25,812 (27.6)	0.003
Neoplasms	19,585 (20.2)	47,951 (21.0)	0.021	18,920 (20.2)	18,629 (19.9)	0.008
Disorders of thyroid gland	18,190 (18.7)	38,145 (16.7)	0.053	17,228 (18.4)	16,987 (18.2)	0.007
Chronic pain, not elsewhere classified	18,031 (18.6)	33,912 (14.9)	0.100	16,841 (18.0)	16,419 (17.6)	0.012
Depressive episode	15,284 (15.7)	29,976 (13.1)	0.074	14,299 (15.3)	14,128 (15.1)	0.005
Asthma	11,973 (12.3)	22,310 (9.8)	0.082	11,160 (11.9)	11,004 (11.8)	0.005
Diseases of liver	11,645 (12.0)	22,011 (9.6)	0.076	10,874 (11.6)	10,709 (11.5)	0.006
COVID-19	8,593 (8.9)	17,551 (7.7)	0.042	8,078 (8.6)	7,875 (8.4)	0.008

Major depressive disorder, recurrent	7,092 (7.3)	11,304 (5.0)	0.098	6,356 (6.8)	6,229 (6.7)	0.005
Sleep disorders not due to a substance or known physiological condition	3,543 (3.7)	5,521 (2.4)	0.072	3,143 (3.4)	3,045 (3.3)	0.006
Other disorders of bone density and structure	3,625 (3.7)	8,768 (3.8)	0.006	3,512 (3.8)	3,354 (3.6)	0.009
Other symptoms and signs involving cognitive functions and awareness	3,629 (3.7)	12,714 (5.6)	0.087	3,564 (3.8)	3,385 (3.6)	0.010
Slipping, tripping, stumbling and falls	4,016 (4.1)	11,811 (5.2)	0.049	3,903 (4.2)	3,754 (4.0)	0.008
Extrapyramidal and movement disorders	3,462 (3.6)	7,698 (3.4)	0.011	3,254 (3.5)	3,093 (3.3)	0.010
Bipolar disorder	2,622 (2.7)	6,813 (3.0)	0.017	2,554 (2.7)	2,458 (2.6)	0.006
Psoriasis	2,122 (2.2)	3,580 (1.6)	0.046	1,937 (2.1)	1,946 (2.1)	<0.001
Age-related cataract	4,500 (4.6)	11,287 (4.9)	0.014	4,378 (4.7)	4,234 (4.5)	0.007
Somnolence, stupor and coma	1,416 (1.5)	2,875 (1.3)	0.017	1,294 (1.4)	1,290 (1.4)	<0.001
Certain disorders involving the immune mechanism	1,632 (1.7)	3,165 (1.4)	0.024	1,517 (1.6)	1,443 (1.5)	0.006
Glaucoma	2,640 (2.7)	7,316 (3.2)	0.029	2,566 (2.7)	2,525 (2.7)	0.003
History of falling	1,608 (1.7)	5,398 (2.4)	0.050	1,586 (1.7)	1,457 (1.6)	0.011
Personal history of (healed) traumatic fracture	368 (0.4)	790 (0.3)	0.005	345 (0.4)	337 (0.4)	0.001
Repeated falls	792 (0.8)	3,035 (1.3)	0.050	779 (0.8)	734 (0.8)	0.005
Long term (current) use of systemic steroids	856 (0.9)	2,399 (1.1)	0.017	833 (0.9)	784 (0.8)	0.006
**Procedure**						
Encounter for screening for osteoporosis	2,316 (2.4)	4,782 (2.1)	0.020	2,219 (2.4)	2,100 (2.2)	0.008
Dual-energy x-ray absorptiometry (dxa), bone density study, 1 or more sites	4,078 (4.2)	8,445 (3.7)	0.026	3,879 (4.1)	3,687 (3.9)	0.010
**Medications**						
Glucocorticoids	42,236 (43.5)	88,982 (39.0)	0.092	40,009 (42.8)	39,095 (41.8)	0.020
Antihistamines for systemic use	24,871 (25.6)	56,248 (24.6)	0.023	23,757 (25.4)	23,143 (24.7)	0.015
Selective serotonin reuptake inhibitors	15,890 (16.4)	29,920 (13.1)	0.092	14,705 (15.7)	14,459 (15.5)	0.007
Antiepileptics	20,644 (21.3)	46,918 (20.6)	0.017	19,602 (21.0)	19,137 (20.5)	0.012
Other antidepressants	16,911 (17.4)	31,916 (14.0)	0.095	15,560 (16.6)	15,309 (16.4)	0.007
Vitamin D and analogues	12,532 (12.9)	29,290 (12.8)	0.002	12,071 (12.9)	11,927 (12.8)	0.005
Thyroid therapy	11,068 (11.4)	24,602 (10.8)	0.020	10,546 (11.3)	10,291 (11.0)	0.009

Chemotherapeutics for topical use	8,278 (8.5)	17,161 (7.5)	0.037	7,834 (8.4)	7,720 (8.3)	0.004
Antipsychotics	10,153 (10.5)	31,531 (13.8)	0.103	10,031 (10.7)	9,502 (10.2)	0.018
Metformin	47,053 (48.5)	122,058 (53.5)	0.100	46,032 (49.2)	45,768 (48.9)	0.006
Antiparasitic products, insecticides and repellents	6,464 (6.7)	13,934 (6.1)	0.023	6,177 (6.6)	6,110 (6.5)	0.003
COVID-19 vaccine	16,317 (16.8)	41,405 (18.1)	0.035	15,910 (17.0)	15,432 (16.5)	0.014
Antineoplastic agents	5,940 (6.1)	13,381 (5.9)	0.011	5,624 (6.0)	5,418 (5.8)	0.009
Antimigraine preparations	4,782 (4.9)	8,511 (3.7)	0.059	4,361 (4.7)	4,290 (4.6)	0.004
Immunosuppressants	2,729 (2.8)	4,804 (2.1)	0.046	2,481 (2.7)	2,428 (2.6)	0.004
Drugs used in addictive disorders	4,318 (4.4)	12,387 (5.4)	0.045	4,221 (4.5)	4,012 (4.3)	0.011
Non-selective monoamine reuptake inhibitors	2,920 (3.0)	5,461 (2.4)	0.038	2,715 (2.9)	2,653 (2.8)	0.004
Anti-Parkinson drugs	2,902 (3.0)	7,064 (3.1)	0.006	2,781 (3.0)	2,683 (2.9)	0.006
Aromatase inhibitors	824 (0.8)	1,935 (0.8)	<0.001	801 (0.9)	775 (0.8)	0.003
Cardiovascular agents,other	1,182 (1.2)	3,552 (1.6)	0.029	1,151 (1.2)	1,132 (1.2)	0.002
Bisphosphonates	701 (0.7)	1,729 (0.8)	0.004	663 (0.7)	651 (0.7)	0.002
Alendronate	533 (0.5)	1,312 (0.6)	0.003	500 (0.5)	502 (0.5)	<0.001
Other drugs affecting bone structure and mineralization	171 (0.2)	377 (0.2)	0.003	155 (0.2)	145 (0.2)	0.003
Denosumab	153 (0.2)	334 (0.1)	0.003	138 (0.1)	129 (0.1)	0.003
Zoledronic acid	131 (0.1)	279 (0.1)	0.004	125 (0.1)	120 (0.1)	0.001
Ibandronate	48 (0.0)	130 (0.1)	0.003	45 (0.0)	38 (0.0)	0.004
Romosozumab	≤10 (0.0)	13 (0.0)	0.005	≤10 (0.0)	≤10 (0.0)	<0.001
Risedronic acid	≤10 (0.0)	≤10 (0.0)	0.007	≤10 (0.0)	≤10 (0.0)	<0.001
Teriparatide	12 (0.0)	11 (0.0)	0.008	≤10 (0.0)	≤10 (0.0)	<0.001
Abaloparatide	≤10 (0.0)	≤10 (0.0)	0.007	≤10 (0.0)	≤10 (0.0)	<0.001
**Laboratory**						
**BMI**						
Mean ± SD, kg/m^2	37.2 ± 7.4	34.0 ± 6.5		37.1 ± 7.4	35.3 ± 6.8	
≥30 kg/m^2	79,415 (81.8)	165,059 (72.3)	0.227	76,037 (81.3)	75,994 (81.3)	0.001
**SBP**						
Mean ± SD, mm[hg]	130.2 ± 17.1	131.2 ± 18.4		130.3 ± 17.1	130.6 ± 18.0	
≥140 mm[hg]	58,435 (60.2)	140,032 (61.4)	0.024	56,486 (60.4)	56,091 (60.0)	0.009
**eGFR**						
Mean ± SD, ml/min/(1.73_m^2)	83.6 ± 25.7	84.1 ± 27.2		83.7 ± 25.7	85.1 ± 27.4	
≥60 ml/min/(1.73_m^2)	75,566 (77.9)	181,146 (79.4)	0.037	73,057 (78.1)	72,429 (77.4)	0.016
**TG**						
Mean ± SD, mg/dl	169.4 ± 139.6	165.7 ± 149.9		168.5 ± 139.8	177.0 ± 168.0	
≥200 mg/dl	24,263 (25.0)	45,053 (19.7)	0.126	22,829 (24.4)	22,762 (24.3)	0.002
**LDL-C**						
Mean ± SD, mg/dl	94.1 ± 37.3	92.4 ± 37.7		93.8 ± 37.3	95.8 ± 38.2	
≥190 mg/dl	1,844 (1.9)	3,454 (1.5)	0.030	1,747 (1.9)	1,713 (1.8)	0.003

**HbA1C**						
Mean ± SD, %	7.3 ± 1.8	7.2 ± 1.7		7.3 ± 1.8	7.3 ± 1.7	
≥7%	44,122 (45.5)	87,204 (38.2)	0.147	41,920 (44.8)	42,542 (45.5)	0.013

Data are presented as mean ± SD for continuous variables and n (%) for categorical variables. SMD, standardized mean difference. A post-matching SMD <0.10 indicates adequate balance. Matching was performed using 1:1 nearest-neighbor propensity score matching with a caliper of 0.2 SD of the logit propensity score based on 215 covariates. SMD, standardized mean difference; COVID-19, coronavirus disease 2019; BMI, body mass index; SBP, systolic blood pressure; eGFR, estimated glomerular filtration rate; TG, triglycerides; LDL-C, low-density lipoprotein cholesterol; HbA1c, glycated hemoglobin A1c.

Supplementary Table 7. Baseline Characteristics Before and After Propensity Score Matching: Semaglutide Versus Naltrexone–Bupropion in Obesity Without Type 2 Diabetes.

Characteristic	Before Matching			After Matching		
	Semaglutide (n = 88,835)	Naltrexone/ bupropion (n = 11,937)	SMD	Semaglutide (n = 10,323)	Naltrexone/ bupropion (n = 10,323)	SMD
**Age**						
Mean ± SD	48.0 ± 14.9	45.5 ± 14.0		46.4 ± 14.0	45.9 ± 14.1	
18-44 years	36,399 (41.0)	5,938 (49.7)	0.177	4,892 (47.4)	4,984 (48.3)	0.018
45-64 years	38,432 (43.3)	4,678 (39.2)	0.083	4,285 (41.5)	4,140 (40.1)	0.029
65-74 years	10,142 (11.4)	1,088 (9.1)	0.076	954 (9.2)	982 (9.5)	0.009
≥75 years	3,004 (3.4)	211 (1.8)	0.102	177 (1.7)	196 (1.9)	0.014
**Sex (%)**						
Male	25,075 (28.2)	2,846 (23.8)	0.100	2,142 (20.8)	2,339 (22.7)	0.046
Female	63,685 (71.7)	9,074 (76.0)	0.099	8,166 (79.1)	7,971 (77.2)	0.046
**Ethnicity (%)**						
Hispanic or Latino	6,096 (6.9)	719 (6.0)	0.034	596 (5.8)	625 (6.1)	0.012
Not Hispanic or Latino	67,233 (75.7)	8,360 (70.0)	0.127	7,531 (73.0)	7,331 (71.0)	0.043
**Race (%)**						
White	63,668 (71.7)	9,418 (78.9)	0.168	8,078 (78.3)	8,030 (77.8)	0.011
Black or African American	14,452 (16.3)	1,389 (11.6)	0.134	1,270 (12.3)	1,280 (12.4)	0.003
Other Races	2,433 (2.7)	344 (2.9)	0.009	279 (2.7)	305 (3.0)	0.015
**Socioeconomic determinants**						
Persons with potential health hazards related to socioeconomic and psychosocial circumstances	2,462 (2.8)	992 (8.3)	0.244	626 (6.1)	631 (6.1)	0.002
**Measures of healthcare utilization**						
Visit	88,635 (99.8)	11,916 (99.8)	0.011	10,298 (99.8)	10,302 (99.8)	0.008
Visit: ambulatory	85,275 (96.0)	11,393 (95.4)	0.027	9,970 (96.6)	9,893 (95.8)	0.039
Visit: emergency	20,956 (23.6)	3,544 (29.7)	0.138	2,957 (28.6)	2,896 (28.1)	0.013
**Comorbidities**						
Disorders of lipoprotein metabolism and other lipidemias	34,555 (38.9)	3,979 (33.3)	0.116	3,693 (35.8)	3,549 (34.4)	0.029
Sleep disorders	18,879 (21.3)	3,004 (25.2)	0.093	2,627 (25.4)	2,542 (24.6)	0.019
Neoplasms	15,216 (17.1)	2,159 (18.1)	0.025	1,860 (18.0)	1,918 (18.6)	0.015
Disorders of thyroid gland	13,053 (14.7)	1,788 (15.0)	0.008	1,623 (15.7)	1,564 (15.2)	0.016
Chronic pain, not elsewhere classified	12,410 (14.0)	2,005 (16.8)	0.078	1,778 (17.2)	1,710 (16.6)	0.018
Depressive episode	11,638 (13.1)	3,361 (28.2)	0.379	2,526 (24.5)	2,580 (25.0)	0.012
Asthma	10,148 (11.4)	1,578 (13.2)	0.055	1,368 (13.3)	1,343 (13.0)	0.007
Diseases of liver	6,800 (7.7)	1,058 (8.9)	0.044	831 (8.1)	882 (8.5)	0.018

COVID-19	5,565 (6.3)	752 (6.3)	0.001	675 (6.5)	654 (6.3)	0.008
Major depressive disorder, recurrent	5,129 (5.8)	1,959 (16.4)	0.344	1,426 (13.8)	1,423 (13.8)	<0.001
Sleep disorders not due to a substance or known physiological condition	3,070 (3.5)	595 (5.0)	0.076	497 (4.8)	501 (4.9)	0.002
Other disorders of bone density and structure	2,389 (2.7)	377 (3.2)	0.028	310 (3.0)	326 (3.2)	0.009
Other symptoms and signs involving cognitive functions and awareness	2,253 (2.5)	628 (5.3)	0.141	445 (4.3)	473 (4.6)	0.013
Slipping, tripping, stumbling and falls	2,227 (2.5)	484 (4.1)	0.087	372 (3.6)	387 (3.7)	0.008
Extrapyramidal and movement disorders	1,992 (2.2)	385 (3.2)	0.060	300 (2.9)	305 (3.0)	0.003
Bipolar disorder	1,948 (2.2)	560 (4.7)	0.137	386 (3.7)	389 (3.8)	0.002
Psoriasis	1,649 (1.9)	234 (2.0)	0.008	209 (2.0)	201 (1.9)	0.006
Age-related cataract	1,281 (1.4)	181 (1.5)	0.006	157 (1.5)	158 (1.5)	<0.001
Somnolence, stupor and coma	1,200 (1.4)	243 (2.0)	0.053	188 (1.8)	191 (1.9)	0.002
Certain disorders involving the immune mechanism	1,039 (1.2)	167 (1.4)	0.020	150 (1.5)	140 (1.4)	0.008
Glaucoma	859 (1.0)	122 (1.0)	0.006	95 (0.9)	110 (1.1)	0.015
History of falling	653 (0.7)	92 (0.8)	0.004	78 (0.8)	76 (0.7)	0.002
Personal history of (healed) traumatic fracture	249 (0.3)	51 (0.4)	0.025	36 (0.3)	44 (0.4)	0.012
Repeated falls	231 (0.3)	53 (0.4)	0.031	40 (0.4)	42 (0.4)	0.003
Long term (current) use of systemic steroids	474 (0.5)	79 (0.7)	0.017	64 (0.6)	69 (0.7)	0.006
**Procedure**						
Encounter for screening for osteoporosis	1,411 (1.6)	209 (1.8)	0.013	185 (1.8)	186 (1.8)	<0.001
Dual-energy x-ray absorptiometry (dxa), bone density study, 1 or more sites	2,379 (2.7)	404 (3.4)	0.041	350 (3.4)	359 (3.5)	0.005
**Medications**						
Glucocorticoids	37,561 (42.3)	5,271 (44.2)	0.038	4,728 (45.8)	4,597 (44.5)	0.026
Antihistamines for systemic use	22,120 (24.9)	3,171 (26.6)	0.038	2,725 (26.4)	2,688 (26.0)	0.008
Selective serotonin reuptake inhibitors	16,340 (18.4)	3,502 (29.3)	0.259	2,805 (27.2)	2,810 (27.2)	0.001
Antiepileptics	13,192 (14.8)	2,533 (21.2)	0.166	1,989 (19.3)	2,001 (19.4)	0.003
Other antidepressants	11,768 (13.2)	5,600 (46.9)	0.789	4,041 (39.1)	4,059 (39.3)	0.004
Vitamin D and analogues	9,485 (10.7)	1,322 (11.1)	0.013	1,113 (10.8)	1,109 (10.7)	0.001

Thyroid therapy	8,628 (9.7)	1,134 (9.5)	0.007	1,025 (9.9)	985 (9.5)	0.013
Chemotherapeutics for topical use	7,698 (8.7)	1,162 (9.7)	0.037	991 (9.6)	992 (9.6)	<0.001
Antipsychotics	7,911 (8.9)	2,048 (17.2)	0.247	1,499 (14.5)	1,508 (14.6)	0.002
Metformin	7,479 (8.4)	750 (6.3)	0.082	673 (6.5)	686 (6.6)	0.005
Antiparasitic products, insecticides and repellents	6,999 (7.9)	1,184 (9.9)	0.072	984 (9.5)	987 (9.6)	<0.001
COVID-19 vaccine	6,543 (7.4)	1,082 (9.1)	0.062	954 (9.2)	937 (9.1)	0.006
Antineoplastic agents	5,641 (6.3)	810 (6.8)	0.018	734 (7.1)	723 (7.0)	0.004
Antimigraine preparations	5,309 (6.0)	1,049 (8.8)	0.108	835 (8.1)	833 (8.1)	<0.001
Immunosuppressants	2,714 (3.1)	347 (2.9)	0.009	274 (2.7)	319 (3.1)	0.026
Drugs used in addictive disorders	2,644 (3.0)	3,321 (27.8)	0.733	1,822 (17.6)	1,730 (16.8)	0.024
Non-selective monoamine reuptake inhibitors	2,165 (2.4)	418 (3.5)	0.063	350 (3.4)	337 (3.3)	0.007
Anti-Parkinson drugs	1,729 (1.9)	317 (2.7)	0.047	230 (2.2)	254 (2.5)	0.015
Aromatase inhibitors	724 (0.8)	56 (0.5)	0.043	49 (0.5)	52 (0.5)	0.004
Cardiovascular agents,other	830 (0.9)	126 (1.1)	0.012	103 (1.0)	106 (1.0)	0.003
Bisphosphonates	399 (0.4)	44 (0.4)	0.013	32 (0.3)	40 (0.4)	0.013
Alendronate	301 (0.3)	35 (0.3)	0.008	24 (0.2)	32 (0.3)	0.015
Other drugs affecting bone structure and mineralization	82 (0.1)	≤10 (0.1)	0.003	≤10 (0.1)	≤10 (0.1)	<0.001
Denosumab	70 (0.1)	≤10 (0.1)	0.002	≤10 (0.1)	≤10 (0.1)	<0.001
Zoledronic acid	64 (0.1)	≤10 (0.1)	0.004	≤10 (0.1)	≤10 (0.1)	<0.001
Ibandronate	40 (0.0)	≤10 (0.1)	0.015	≤10 (0.1)	≤10 (0.1)	<0.001
Romosozumab	≤10 (0.0)	≤10 (0.1)	0.033	N/A	≤10 (0.1)	N/A
Risedronic acid	≤10 (0.0)	N/A	N/A	N/A	N/A	N/A
Teriparatide	11 (0.0)	≤10 (0.1)	0.033	N/A	≤10 (0.1)	N/A
Abaloparatide	≤10 (0.0)	N/A	N/A	N/A	N/A	N/A
**Laboratory**						
**BMI**						
Mean ± SD, kg/m^2	37.2 ± 7.3	35.9 ± 7.2		37.1 ± 7.4	36.2 ± 7.2	
≥30 kg/m^2	71,354 (80.3)	9,034 (75.7)	0.112	8,068 (78.2)	7,956 (77.1)	0.026
**SBP**						
Mean ± SD, mm[hg]	127.7 ± 15.9	125.4 ± 16.3		126.8 ± 15.6	125.7 ± 16.2	
≥140 mm[hg]	39,485 (44.4)	5,599 (46.9)	0.049	4,860 (47.1)	4,790 (46.4)	0.014
**eGFR**						
Mean ± SD, ml/min/(1.73_m^2)	86.2 ± 21.8	86.8 ± 21.5		86.9 ± 21.8	86.6 ± 21.4	
≥60 ml/min/(1.73_m^2)	62,474 (70.3)	9,071 (76.0)	0.128	7,891 (76.4)	7,792 (75.5)	0.023
**TG**						
Mean ± SD, mg/dl	133.9 ± 80.9	133.4 ± 82.3		133.2 ± 76.5	133.1 ± 82.1	
≥200 mg/dl	10,576 (11.9)	1,349 (11.3)	0.019	1,202 (11.6)	1,181 (11.4)	0.006
**LDL-C**						
Mean ± SD, mg/dl	109.7 ± 34.5	111.7 ± 33.4		109.9 ± 34.0	112.0 ± 33.2	

≥190 mg/dl	1,606 (1.8)	223 (1.9)	0.004	173 (1.7)	189 (1.8)	0.012
**HbA1C**						
Mean ± SD, %	5.5 ± 0.6	5.4 ± 0.4		5.5 ± 0.4	5.4 ± 0.4	
≥7%	792 (0.9)	11 (0.1)	0.115	11 (0.1)	11 (0.1)	<0.001

Data are presented as mean ± SD for continuous variables and n (%) for categorical variables. SMD, standardized mean difference. A post-matching SMD <0.10 indicates adequate balance. Matching was performed using 1:1 nearest-neighbor propensity score matching with a caliper of 0.2 SD of the logit propensity score based on 215 covariates. SMD, standardized mean difference; COVID-19, coronavirus disease 2019; BMI, body mass index; SBP, systolic blood pressure; eGFR, estimated glomerular filtration rate; TG, triglycerides; LDL-C, low-density lipoprotein cholesterol; HbA1c, glycated hemoglobin A1c.

Supplementary Table 8. Baseline Characteristics Before and After Propensity Score Matching: Semaglutide Versus Phentermine in Obesity Without Type 2 Diabetes.

	Before Matching			After Matching		
Characteristic	Semaglutide (n = 88,835)	Phentermine (n = 37,592)	SMD	Semaglutide (n = 36,674)	Phentermine (n = 36,674)	SMD
**Age**						
Mean ± SD	48.0 ± 14.9	43.1 ± 13.3		43.7 ± 13.4	43.2 ± 13.3	
18-44 years	36,399 (41.0)	21,064 (56.0)	0.305	20,453 (55.8)	20,238 (55.2)	0.012
45-64 years	38,432 (43.3)	13,938 (37.1)	0.126	13,696 (37.3)	13,847 (37.8)	0.009
65-74 years	10,142 (11.4)	2,148 (5.7)	0.205	2,087 (5.7)	2,147 (5.9)	0.007
≥75 years	3,004 (3.4)	297 (0.8)	0.182	297 (0.8)	297 (0.8)	<0.001
**Sex (%)**						
Male	25,075 (28.2)	5,541 (14.7)	0.333	5,552 (15.1)	5,541 (15.1)	<0.001
Female	63,685 (71.7)	32,034 (85.2)	0.334	31,104 (84.8)	31,116 (84.8)	<0.001
**Ethnicity (%)**						
Hispanic or Latino	6,096 (6.9)	3,289 (8.7)	0.070	3,075 (8.4)	3,196 (8.7)	0.012
Not Hispanic or Latino	67,233 (75.7)	24,722 (65.8)	0.219	25,043 (68.3)	24,668 (67.3)	0.022
**Race (%)**						
White	63,668 (71.7)	26,317 (70.0)	0.037	25,643 (69.9)	25,777 (70.3)	0.008
Black or African American	14,452 (16.3)	6,789 (18.1)	0.048	6,849 (18.7)	6,616 (18.0)	0.016
Other Races	2,433 (2.7)	1,676 (4.5)	0.092	1,457 (4.0)	1,498 (4.1)	0.006
**Socioeconomic determinants**						
Persons with potential health hazards related to socioeconomic and psychosocial circumstances	2,465 (2.8)	1,016 (2.7)	0.004	951 (2.6)	993 (2.7)	0.007
**Measures of healthcare utilization**						
Visit	88,635 (99.8)	37,529 (99.8)	0.013	36,606 (99.8)	36,611 (99.8)	0.003
Visit: ambulatory	85,275 (96.0)	34,978 (93.0)	0.130	34,697 (94.6)	34,557 (94.2)	0.017
Visit: emergency	20,969 (23.6)	8,148 (21.7)	0.046	7,820 (21.3)	8,074 (22.0)	0.017
**Comorbidities**						
Disorders of lipoprotein metabolism and other lipidemias	34,564 (38.9)	10,099 (26.9)	0.258	9,920 (27.0)	10,019 (27.3)	0.006
Sleep disorders	18,888 (21.3)	5,706 (15.2)	0.158	5,395 (14.7)	5,654 (15.4)	0.020
Neoplasms	15,231 (17.1)	5,572 (14.8)	0.063	5,266 (14.4)	5,481 (14.9)	0.017
Disorders of thyroid gland	13,056 (14.7)	5,429 (14.4)	0.007	5,230 (14.3)	5,328 (14.5)	0.008
Chronic pain, not elsewhere classified	12,422 (14.0)	5,270 (14.0)	0.001	4,915 (13.4)	5,116 (13.9)	0.016
Depressive episode	11,647 (13.1)	5,190 (13.8)	0.020	4,949 (13.5)	5,069 (13.8)	0.010
Asthma	10,153 (11.4)	3,930 (10.5)	0.031	3,734 (10.2)	3,866 (10.5)	0.012
Diseases of liver	6,807 (7.7)	1,753 (4.7)	0.125	1,704 (4.6)	1,743 (4.8)	0.005
COVID-19	5,577 (6.3)	2,207 (5.9)	0.017	2,112 (5.8)	2,162 (5.9)	0.006

Major depressive disorder, recurrent	5,130 (5.8)	2,084 (5.5)	0.010	1,967 (5.4)	2,047 (5.6)	0.010
Sleep disorders not due to a substance or known physiological condition	3,071 (3.5)	1,503 (4.0)	0.029	1,413 (3.9)	1,432 (3.9)	0.003
Other disorders of bone density and structure	2,394 (2.7)	751 (2.0)	0.046	711 (1.9)	745 (2.0)	0.007
Other symptoms and signs involving cognitive functions and awareness	2,255 (2.5)	818 (2.2)	0.024	793 (2.2)	806 (2.2)	0.002
Slipping, tripping, stumbling and falls	2,233 (2.5)	882 (2.3)	0.011	785 (2.1)	864 (2.4)	0.015
Extrapyramidal and movement disorders	1,992 (2.2)	686 (1.8)	0.030	603 (1.6)	676 (1.8)	0.015
Bipolar disorder	1,949 (2.2)	689 (1.8)	0.026	633 (1.7)	685 (1.9)	0.011
Psoriasis	1,649 (1.9)	550 (1.5)	0.031	529 (1.4)	543 (1.5)	0.003
Age-related cataract	1,282 (1.4)	335 (0.9)	0.051	335 (0.9)	335 (0.9)	<0.001
Somnolence, stupor and coma	1,202 (1.4)	438 (1.2)	0.017	403 (1.1)	433 (1.2)	0.008
Certain disorders involving the immune mechanism	1,039 (1.2)	330 (0.9)	0.029	305 (0.8)	329 (0.9)	0.007
Glaucoma	860 (1.0)	238 (0.6)	0.038	233 (0.6)	237 (0.6)	0.001
History of falling	654 (0.7)	133 (0.4)	0.052	127 (0.3)	133 (0.4)	0.003
Personal history of (healed) traumatic fracture	250 (0.3)	95 (0.3)	0.006	89 (0.2)	94 (0.3)	0.003
Repeated falls	232 (0.3)	57 (0.2)	0.024	59 (0.2)	57 (0.2)	0.001
Long term (current) use of systemic steroids	475 (0.5)	186 (0.5)	0.006	160 (0.4)	181 (0.5)	0.008
**Procedure**						
Encounter for screening for osteoporosis	1,417 (1.6)	401 (1.1)	0.046	384 (1.0)	399 (1.1)	0.004
Dual-energy x-ray absorptiometry (dxa), bone density study, 1 or more sites	2,385 (2.7)	792 (2.1)	0.038	746 (2.0)	780 (2.1)	0.006
**Medications**						
Glucocorticoids	37,590 (42.3)	15,794 (42.0)	0.006	15,045 (41.0)	15,353 (41.9)	0.017
Antihistamines for systemic use	22,141 (24.9)	9,532 (25.4)	0.010	8,984 (24.5)	9,252 (25.2)	0.017
Selective serotonin reuptake inhibitors	16,348 (18.4)	7,082 (18.8)	0.011	6,780 (18.5)	6,928 (18.9)	0.010
Antiepileptics	13,204 (14.9)	4,963 (13.2)	0.048	4,634 (12.6)	4,851 (13.2)	0.018
Other antidepressants	11,777 (13.3)	4,372 (11.6)	0.049	4,159 (11.3)	4,312 (11.8)	0.013
Vitamin D and analogues	9,489 (10.7)	3,567 (9.5)	0.040	3,365 (9.2)	3,534 (9.6)	0.016
Thyroid therapy	8,630 (9.7)	3,378 (9.0)	0.025	3,250 (8.9)	3,325 (9.1)	0.007

Chemotherapeutics for topical use	7,710 (8.7)	3,802 (10.1)	0.049	3,439 (9.4)	3,638 (9.9)	0.018
Antipsychotics	7,918 (8.9)	3,295 (8.8)	0.005	3,040 (8.3)	3,229 (8.8)	0.018
Metformin	7,482 (8.4)	1,747 (4.6)	0.153	1,692 (4.6)	1,744 (4.8)	0.007
Antiparasitic products, insecticides and repellents	7,012 (7.9)	3,478 (9.3)	0.049	3,220 (8.8)	3,356 (9.2)	0.013
COVID-19 vaccine	6,554 (7.4)	2,134 (5.7)	0.069	2,037 (5.6)	2,122 (5.8)	0.010
Antineoplastic agents	5,644 (6.4)	2,307 (6.1)	0.009	2,117 (5.8)	2,231 (6.1)	0.013
Antimigraine preparations	5,315 (6.0)	2,263 (6.0)	0.002	2,113 (5.8)	2,215 (6.0)	0.012
Immunosuppressants	2,715 (3.1)	918 (2.4)	0.038	845 (2.3)	909 (2.5)	0.011
Drugs used in addictive disorders	2,649 (3.0)	844 (2.2)	0.046	795 (2.2)	836 (2.3)	0.008
Non-selective monoamine reuptake inhibitors	2,166 (2.4)	866 (2.3)	0.009	803 (2.2)	843 (2.3)	0.007
Anti-Parkinson drugs	1,730 (1.9)	593 (1.6)	0.028	561 (1.5)	590 (1.6)	0.006
Aromatase inhibitors	724 (0.8)	257 (0.7)	0.015	238 (0.6)	254 (0.7)	0.005
Cardiovascular agents,other	831 (0.9)	191 (0.5)	0.051	198 (0.5)	191 (0.5)	0.003
Bisphosphonates	400 (0.5)	113 (0.3)	0.024	104 (0.3)	112 (0.3)	0.004
Alendronate	302 (0.3)	72 (0.2)	0.029	69 (0.2)	71 (0.2)	0.001
Other drugs affecting bone structure and mineralization	82 (0.1)	26 (0.1)	0.008	22 (0.1)	24 (0.1)	0.002
Denosumab	70 (0.1)	16 (0.0)	0.015	14 (0.0)	16 (0.0)	0.003
Zoledronic acid	64 (0.1)	31 (0.1)	0.004	27 (0.1)	31 (0.1)	0.004
Ibandronate	40 (0.0)	12 (0.0)	0.007	11 (0.0)	12 (0.0)	0.002
Romosozumab	≤10 (0.0)	≤10 (0.0)	0.011	≤10 (0.0)	≤10 (0.0)	<0.001
Risedronic acid	≤10 (0.0)	N/A	N/A	N/A	N/A	N/A
Teriparatide	11 (0.0)	≤10 (0.0)	0.010	≤10 (0.0)	≤10 (0.0)	<0.001
Abaloparatide	≤10 (0.0)	≤10 (0.0)	0.011	≤10 (0.0)	≤10 (0.0)	<0.001
**Laboratory**						
**BMI**						
Mean ± SD, kg/m^2	37.2 ± 7.3	36.0 ± 7.0	0.176	37.3 ± 7.5	36.0 ± 7.0	0.178
≥30 kg/m^2	71,357 (80.3)	28,807 (76.6)	0.090	28,335 (77.3)	28,307 (77.2)	0.002
**SBP**						
Mean ± SD, mm[hg]	127.7 ± 15.9	123.8 ± 15.1	0.252	125.8 ± 15.3	123.9 ± 15.2	0.127
≥140 mm[hg]	39,523 (44.5)	13,263 (35.3)	0.189	13,196 (36.0)	13,201 (36.0)	<0.001
**eGFR**						
Mean ± SD, ml/min/(1.73_m^2)	86.2 ± 21.8	89.7 ± 21.8	0.163	89.4 ± 21.7	89.6 ± 21.8	0.009
≥60 ml/min/(1.73_m^2)	62,512 (70.4)	26,394 (70.2)	0.003	25,578 (69.7)	25,748 (70.2)	0.010
**TG**						
Mean ± SD, mg/dl	133.9 ± 80.9	124.3 ± 73.9	0.124	125.4 ± 70.9	124.8 ± 74.3	0.009
≥200 mg/dl	10,587 (11.9)	3,240 (8.6)	0.109	3,176 (8.7)	3,218 (8.8)	0.004
**LDL-C**						
Mean ± SD, mg/dl	109.7 ± 34.5	113.5 ± 31.8	0.115	111.4 ± 32.5	113.6 ± 31.9	0.069
≥190 mg/dl	1,607 (1.8)	560 (1.5)	0.025	506 (1.4)	552 (1.5)	0.011

**HbA1C**						
Mean ± SD, %	5.5 ± 0.6	5.4 ± 0.4	0.265	5.4 ± 0.4	5.4 ± 0.4	0.076
≥7%	792 (0.9)	21 (0.1)	0.122	22 (0.1)	21 (0.1)	0.001

Data are presented as mean ± SD for continuous variables and n (%) for categorical variables. SMD, standardized mean difference. A post-matching SMD <0.10 indicates adequate balance. Matching was performed using 1:1 nearest-neighbor propensity score matching with a caliper of 0.2 SD of the logit propensity score based on 215 covariates. SMD, standardized mean difference; COVID-19, coronavirus disease 2019; BMI, body mass index; SBP, systolic blood pressure; eGFR, estimated glomerular filtration rate; TG, triglycerides; LDL-C, low-density lipoprotein cholesterol; HbA1c, glycated hemoglobin A1c.

Supplementary Table 9. Baseline Characteristics Before and After Propensity Score Matching: Semaglutide Versus Phentermine–Topiramate in Obesity Without Type 2 Diabetes.

Characteristic	Before Matching			After Matching		
	Semaglutide (n = 88,835)	Phentermine/ topiramate (n = 10,931)	SMD	Semaglutide (n = 10,833)	Phentermine/ topiramate (n = 10,833)	SMD
**Age**						
Mean ± SD	48.0 ± 14.9	42.3 ± 13.1		43.4 ± 13.3	42.4 ± 13.1	
18-44 years	36,399 (41.0)	6,174 (56.5)	0.314	5,997 (55.4)	6,081 (56.1)	0.016
45-64 years	38,432 (43.3)	3,989 (36.5)	0.139	4,067 (37.5)	3,986 (36.8)	0.015
65-74 years	10,142 (11.4)	543 (5.0)	0.237	538 (5.0)	543 (5.0)	0.002
≥75 years	3,004 (3.4)	59 (0.5)	0.206	61 (0.6)	59 (0.5)	0.002
**Sex (%)**						
Male	25,075 (28.2)	1,401 (12.8)	0.389	1,432 (13.2)	1,401 (12.9)	0.008
Female	63,685 (71.7)	9,526 (87.1)	0.390	9,397 (86.7)	9,428 (87.0)	0.008
**Ethnicity (%)**						
Hispanic or Latino	6,096 (6.9)	1,227 (11.2)	0.153	1,237 (11.4)	1,187 (11.0)	0.015
Not Hispanic or Latino	67,233 (75.7)	7,079 (64.8)	0.241	7,022 (64.8)	7,074 (65.3)	0.010
**Race (%)**						
White	63,668 (71.7)	7,754 (70.9)	0.016	7,654 (70.7)	7,688 (71.0)	0.007
Black or African American	14,452 (16.3)	1,714 (15.7)	0.016	1,746 (16.1)	1,711 (15.8)	0.009
Other Races	2,433 (2.7)	513 (4.7)	0.103	498 (4.6)	489 (4.5)	0.004
**Socioeconomic determinants**						
Persons with potential health hazards related to socioeconomic and psychosocial circumstances	2,465 (2.8)	355 (3.2)	0.028	361 (3.3)	352 (3.2)	0.005
**Measures of healthcare utilization**						
Visit	88,635 (99.8)	10,901 (99.7)	0.010	10,807 (99.8)	10,804 (99.7)	0.006
Visit: ambulatory	85,275 (96.0)	10,560 (96.6)	0.032	10,519 (97.1)	10,465 (96.6)	0.029
Visit: emergency	20,969 (23.6)	2,514 (23.0)	0.014	2,548 (23.5)	2,506 (23.1)	0.009
**Comorbidities**						
Disorders of lipoprotein metabolism and other lipidemias	34,564 (38.9)	2,989 (27.3)	0.248	3,034 (28.0)	2,982 (27.5)	0.011
Sleep disorders	18,888 (21.3)	2,046 (18.7)	0.064	2,028 (18.7)	2,029 (18.7)	<0.001
Neoplasms	15,231 (17.1)	1,849 (16.9)	0.006	1,820 (16.8)	1,834 (16.9)	0.003
Disorders of thyroid gland	13,056 (14.7)	1,691 (15.5)	0.022	1,710 (15.8)	1,672 (15.4)	0.010
Chronic pain, not elsewhere classified	12,422 (14.0)	1,777 (16.3)	0.063	1,767 (16.3)	1,759 (16.2)	0.002
Depressive episode	11,647 (13.1)	1,767 (16.2)	0.086	1,725 (15.9)	1,745 (16.1)	0.005
Asthma	10,153 (11.4)	1,442 (13.2)	0.054	1,445 (13.3)	1,429 (13.2)	0.004
Diseases of liver	6,807 (7.7)	675 (6.2)	0.059	695 (6.4)	672 (6.2)	0.009

COVID-19	5,577 (6.3)	624 (5.7)	0.024	653 (6.0)	623 (5.8)	0.012
Major depressive disorder, recurrent	5,130 (5.8)	810 (7.4)	0.066	781 (7.2)	798 (7.4)	0.006
Sleep disorders not due to a substance or known physiological condition	3,071 (3.5)	399 (3.6)	0.010	384 (3.5)	397 (3.7)	0.006
Other disorders of bone density and structure	2,394 (2.7)	251 (2.3)	0.026	253 (2.3)	250 (2.3)	0.002
Other symptoms and signs involving cognitive functions and awareness	2,255 (2.5)	271 (2.5)	0.004	268 (2.5)	270 (2.5)	0.001
Slipping, tripping, stumbling and falls	2,233 (2.5)	301 (2.8)	0.015	312 (2.9)	298 (2.8)	0.008
Extrapyramidal and movement disorders	1,992 (2.2)	247 (2.3)	0.001	249 (2.3)	245 (2.3)	0.002
Bipolar disorder	1,949 (2.2)	231 (2.1)	0.006	211 (1.9)	231 (2.1)	0.013
Psoriasis	1,649 (1.9)	198 (1.8)	0.003	189 (1.7)	194 (1.8)	0.004
Age-related cataract	1,282 (1.4)	105 (1.0)	0.044	99 (0.9)	105 (1.0)	0.006
Somnolence, stupor and coma	1,202 (1.4)	178 (1.6)	0.023	178 (1.6)	175 (1.6)	0.002
Certain disorders involving the immune mechanism	1,039 (1.2)	114 (1.0)	0.012	115 (1.1)	114 (1.1)	<0.001
Glaucoma	860 (1.0)	69 (0.6)	0.038	71 (0.7)	69 (0.6)	0.002
History of falling	654 (0.7)	40 (0.4)	0.050	43 (0.4)	40 (0.4)	0.004
Personal history of (healed) traumatic fracture	250 (0.3)	36 (0.3)	0.009	23 (0.2)	36 (0.3)	0.023
Repeated falls	232 (0.3)	19 (0.2)	0.019	17 (0.2)	19 (0.2)	0.005
Long term (current) use of systemic steroids	475 (0.5)	46 (0.4)	0.017	53 (0.5)	46 (0.4)	0.010
**Procedure**						
Encounter for screening for osteoporosis	1,417 (1.6)	116 (1.1)	0.047	108 (1.0)	116 (1.1)	0.007
Dual-energy x-ray absorptiometry (dxa), bone density study, 1 or more sites	2,385 (2.7)	231 (2.1)	0.037	246 (2.3)	231 (2.1)	0.009
**Medications**						
Glucocorticoids	37,590 (42.3)	4,834 (44.2)	0.039	5,053 (46.6)	4,793 (44.2)	0.048
Antihistamines for systemic use	22,141 (24.9)	2,925 (26.8)	0.042	2,967 (27.4)	2,907 (26.8)	0.012
Selective serotonin reuptake inhibitors	16,348 (18.4)	2,288 (20.9)	0.064	2,326 (21.5)	2,268 (20.9)	0.013
Antiepileptics	13,204 (14.9)	4,253 (38.9)	0.563	4,208 (38.8)	4,155 (38.4)	0.010
Other antidepressants	11,777 (13.3)	1,524 (13.9)	0.020	1,527 (14.1)	1,521 (14.0)	0.002
Vitamin D and analogues	9,489 (10.7)	1,326 (12.1)	0.046	1,311 (12.1)	1,311 (12.1)	<0.001

Thyroid therapy	8,630 (9.7)	1,094 (10.0)	0.010	1,129 (10.4)	1,083 (10.0)	0.014
Chemotherapeutics for topical use	7,710 (8.7)	1,146 (10.5)	0.061	1,221 (11.3)	1,137 (10.5)	0.025
Antipsychotics	7,918 (8.9)	1,140 (10.4)	0.051	1,156 (10.7)	1,135 (10.5)	0.006
Metformin	7,482 (8.4)	811 (7.4)	0.037	817 (7.5)	811 (7.5)	0.002
Antiparasitic products, insecticides and repellents	7,012 (7.9)	1,145 (10.5)	0.089	1,224 (11.3)	1,136 (10.5)	0.026
COVID-19 vaccine	6,554 (7.4)	786 (7.2)	0.007	749 (6.9)	776 (7.2)	0.010
Antineoplastic agents	5,644 (6.4)	808 (7.4)	0.041	822 (7.6)	805 (7.4)	0.006
Antimigraine preparations	5,315 (6.0)	1,217 (11.1)	0.185	1,217 (11.2)	1,178 (10.9)	0.011
Immunosuppressants	2,715 (3.1)	303 (2.8)	0.017	307 (2.8)	303 (2.8)	0.002
Drugs used in addictive disorders	2,649 (3.0)	336 (3.1)	0.005	344 (3.2)	335 (3.1)	0.005
Non-selective monoamine reuptake inhibitors	2,166 (2.4)	390 (3.6)	0.066	404 (3.7)	382 (3.5)	0.011
Anti-Parkinson drugs	1,730 (1.9)	176 (1.6)	0.026	175 (1.6)	176 (1.6)	<0.001
Aromatase inhibitors	724 (0.8)	83 (0.8)	0.006	89 (0.8)	83 (0.8)	0.006
Cardiovascular agents,other	831 (0.9)	76 (0.7)	0.027	71 (0.7)	76 (0.7)	0.006
Bisphosphonates	400 (0.5)	32 (0.3)	0.026	24 (0.2)	32 (0.3)	0.015
Alendronate	302 (0.3)	22 (0.2)	0.027	16 (0.1)	22 (0.2)	0.013
Other drugs affecting bone structure and mineralization	82 (0.1)	≤10 (0.1)	<0.001	≤10 (0.1)	≤10 (0.1)	<0.001
Denosumab	70 (0.1)	≤10 (0.1)	0.004	≤10 (0.1)	≤10 (0.1)	<0.001
Zoledronic acid	64 (0.1)	≤10 (0.1)	0.007	≤10 (0.1)	≤10 (0.1)	<0.001
Ibandronate	40 (0.0)	≤10 (0.1)	0.018	≤10 (0.1)	≤10 (0.1)	<0.001
Romosozumab	≤10 (0.0)	N/A	N/A	N/A	N/A	N/A
Risedronic acid	≤10 (0.0)	N/A	N/A	N/A	N/A	N/A
Teriparatide	11 (0.0)	≤10 (0.1)	0.035	≤10 (0.1)	≤10 (0.1)	<0.001
Abaloparatide	≤10 (0.0)	≤10 (0.1)	0.035	≤10 (0.1)	≤10 (0.1)	<0.001
**Laboratory**						
**BMI**						
Mean ± SD, kg/m^2	37.2 ± 7.3	36.9 ± 7.2		37.4 ± 7.4	36.9 ± 7.2	
≥30 kg/m^2	71,357 (80.3)	8,723 (79.8)	0.013	8,706 (80.4)	8,653 (79.9)	0.012
**SBP**						
Mean ± SD, mm[hg]	127.7 ± 15.9	122.4 ± 15.2		125.2 ± 15.2	122.4 ± 15.2	
≥140 mm[hg]	39,498 (44.5)	3,789 (34.7)	0.201	3,826 (35.3)	3,784 (34.9)	0.008
**eGFR**						
Mean ± SD, ml/min/(1.73_m^2)	86.2 ± 21.8	88.8 ± 21.6		89.0 ± 21.7	88.7 ± 21.6	
≥60 ml/min/(1.73_m^2)	62,495 (70.3)	7,868 (72.0)	0.036	7,800 (72.0)	7,791 (71.9)	0.002
**TG**						
Mean ± SD, mg/dl	133.9 ± 80.9	125.4 ± 70.4		127.8 ± 78.9	125.5 ± 70.6	
≥200 mg/dl	10,587 (11.9)	1,005 (9.2)	0.089	993 (9.2)	1,004 (9.3)	0.004
**LDL-C**						
Mean ± SD, mg/dl	109.7 ± 34.5	112.3 ± 31.3		111.4 ± 32.5	112.3 ± 31.3	

≥190 mg/dl	1,607 (1.8)	166 (1.5)	0.023	177 (1.6)	164 (1.5)	0.010
**HbA1C**						
Mean ± SD, %	5.5 ± 0.6	5.4 ± 0.4		5.4 ± 0.4	5.4 ± 0.4	
≥7%	792 (0.9)	11 (0.1)	0.114	11 (0.1)	11 (0.1)	<0.001

Data are presented as mean ± SD for continuous variables and n (%) for categorical variables. SMD, standardized mean difference. A post-matching SMD <0.10 indicates adequate balance. Matching was performed using 1:1 nearest-neighbor propensity score matching with a caliper of 0.2 SD of the logit propensity score based on 215 covariates. SMD, standardized mean difference; COVID-19, coronavirus disease 2019; BMI, body mass index; SBP, systolic blood pressure; eGFR, estimated glomerular filtration rate; TG, triglycerides; LDL-C, low-density lipoprotein cholesterol; HbA1c, glycated hemoglobin A1c.

Supplementary Table 10. Baseline Characteristics Before and After Propensity Score Matching: Semaglutide Versus Usual Care in Obesity Without Type 2 Diabetes.

Characteristic	Before Matching			After Matching		
	Semaglutide (n = 88,835)	Usual care (n = 82,570)	SMD	Semaglutide (n = 56,225)	Usual care (n = 56,225)	SMD
**Age**						
Mean ± SD	48.0 ± 14.9	47.6 ± 17.7		47.1 ± 15.8	46.7 ± 16.9	
18-44 years	36,399 (41.0)	37,068 (44.9)	0.079	26,139 (46.5)	25,784 (45.9)	0.013
45-64 years	38,432 (43.3)	26,621 (32.2)	0.229	19,627 (34.9)	19,925 (35.4)	0.011
65-74 years	10,142 (11.4)	12,029 (14.6)	0.094	7,144 (12.7)	7,263 (12.9)	0.006
≥75 years	3,004 (3.4)	5,500 (6.7)	0.151	2,564 (4.6)	2,518 (4.5)	0.004
**Sex (%)**						
Male	25,075 (28.2)	25,469 (30.8)	0.057	15,586 (27.7)	16,000 (28.5)	0.016
Female	63,685 (71.7)	56,983 (69.0)	0.059	40,577 (72.2)	40,164 (71.4)	0.016
**Ethnicity (%)**						
Hispanic or Latino	6,096 (6.9)	9,043 (11.0)	0.144	5,317 (9.5)	5,199 (9.2)	0.007
Not Hispanic or Latino	67,233 (75.7)	56,359 (68.3)	0.166	39,968 (71.1)	40,179 (71.5)	0.008
**Race (%)**						
White	63,668 (71.7)	50,528 (61.2)	0.223	36,725 (65.3)	36,979 (65.8)	0.010
Black or African American	14,452 (16.3)	14,695 (17.8)	0.041	10,097 (18.0)	10,038 (17.9)	0.003
Other Races	2,433 (2.7)	4,423 (5.4)	0.133	2,230 (4.0)	2,103 (3.7)	0.012
**Socioeconomic determinants**						
Persons with potential health hazards related to socioeconomic and psychosocial circumstances	2,462 (2.8)	3,097 (3.8)	0.055	1,877 (3.3)	1,803 (3.2)	0.007
**Measures of healthcare utilization**						
Visit	88,635 (99.8)	82,215 (99.6)	0.036	56,026 (99.6)	56,054 (99.7)	0.009
Visit: ambulatory	85,275 (96.0)	71,339 (86.4)	0.344	52,673 (93.7)	52,675 (93.7)	<0.001
Visit: emergency	20,956 (23.6)	18,939 (22.9)	0.015	13,356 (23.8)	13,392 (23.8)	0.002
**Comorbidities**						
Disorders of lipoprotein metabolism and other lipidemias	34,555 (38.9)	24,025 (29.1)	0.208	17,945 (31.9)	18,040 (32.1)	0.004
Sleep disorders	18,879 (21.3)	12,007 (14.5)	0.176	9,278 (16.5)	9,304 (16.5)	0.001
Neoplasms	15,216 (17.1)	12,681 (15.4)	0.048	9,123 (16.2)	9,184 (16.3)	0.003
Disorders of thyroid gland	13,053 (14.7)	10,462 (12.7)	0.059	7,535 (13.4)	7,473 (13.3)	0.003
Chronic pain, not elsewhere classified	12,410 (14.0)	8,222 (10.0)	0.124	6,391 (11.4)	6,449 (11.5)	0.003
Depressive episode	11,638 (13.1)	9,070 (11.0)	0.065	6,639 (11.8)	6,680 (11.9)	0.002
Asthma	10,148 (11.4)	7,602 (9.2)	0.073	5,669 (10.1)	5,776 (10.3)	0.006
Diseases of liver	6,800 (7.7)	4,777 (5.8)	0.075	3,617 (6.4)	3,626 (6.4)	<0.001
COVID-19	5,565 (6.3)	3,686 (4.5)	0.080	2,802 (5.0)	2,795 (5.0)	<0.001

Major depressive disorder, recurrent	5,129 (5.8)	3,990 (4.8)	0.042	2,918 (5.2)	2,910 (5.2)	<0.001
Sleep disorders not due to a substance or known physiological condition	3,070 (3.5)	1,818 (2.2)	0.076	1,346 (2.4)	1,395 (2.5)	0.006
Other disorders of bone density and structure	2,389 (2.7)	1,767 (2.1)	0.036	1,301 (2.3)	1,305 (2.3)	<0.001
Other symptoms and signs involving cognitive functions and awareness	2,253 (2.5)	2,262 (2.7)	0.013	1,428 (2.5)	1,464 (2.6)	0.004
Slipping, tripping, stumbling and falls	2,227 (2.5)	1,826 (2.2)	0.019	1,312 (2.3)	1,309 (2.3)	<0.001
Extrapyramidal and movement disorders	1,992 (2.2)	1,627 (2.0)	0.019	1,113 (2.0)	1,156 (2.1)	0.005
Bipolar disorder	1,948 (2.2)	2,826 (3.4)	0.074	1,563 (2.8)	1,572 (2.8)	<0.001
Psoriasis	1,649 (1.9)	956 (1.2)	0.057	730 (1.3)	771 (1.4)	0.006
Age-related cataract	1,281 (1.4)	1,049 (1.3)	0.015	762 (1.4)	777 (1.4)	0.002
Somnolence, stupor and coma	1,200 (1.4)	830 (1.0)	0.032	669 (1.2)	658 (1.2)	0.002
Certain disorders involving the immune mechanism	1,039 (1.2)	782 (0.9)	0.022	583 (1.0)	591 (1.1)	0.001
Glaucoma	859 (1.0)	830 (1.0)	0.004	580 (1.0)	569 (1.0)	0.002
History of falling	653 (0.7)	542 (0.7)	0.009	426 (0.8)	395 (0.7)	0.006
Personal history of (healed) traumatic fracture	249 (0.3)	185 (0.2)	0.011	145 (0.3)	136 (0.2)	0.003
Repeated falls	231 (0.3)	218 (0.3)	<0.001	151 (0.3)	141 (0.3)	0.003
Long term (current) use of systemic steroids	474 (0.5)	353 (0.4)	0.015	269 (0.5)	270 (0.5)	<0.001
**Procedure**						
Encounter for screening for osteoporosis	1,411 (1.6)	797 (1.0)	0.056	630 (1.1)	654 (1.2)	0.004
Dual-energy x-ray absorptiometry (dxa), bone density study, 1 or more sites	2,379 (2.7)	1,930 (2.3)	0.022	1,317 (2.3)	1,349 (2.4)	0.004
**Medications**						
Glucocorticoids	37,561 (42.3)	31,582 (38.2)	0.082	22,039 (39.2)	22,052 (39.2)	<0.001
Antihistamines for systemic use	22,120 (24.9)	21,015 (25.5)	0.013	13,994 (24.9)	14,051 (25.0)	0.002
Selective serotonin reuptake inhibitors	16,340 (18.4)	13,124 (15.9)	0.066	9,111 (16.2)	9,166 (16.3)	0.003
Antiepileptics	13,192 (14.8)	13,796 (16.7)	0.051	8,465 (15.1)	8,504 (15.1)	0.002
Other antidepressants	11,768 (13.2)	10,352 (12.5)	0.021	6,660 (11.8)	6,823 (12.1)	0.009
Vitamin D and analogues	9,485 (10.7)	10,561 (12.8)	0.066	6,528 (11.6)	6,577 (11.7)	0.003
Thyroid therapy	8,628 (9.7)	8,523 (10.3)	0.020	5,277 (9.4)	5,345 (9.5)	0.004

Chemotherapeutics for topical use	7,698 (8.7)	7,435 (9.0)	0.012	5,027 (8.9)	5,017 (8.9)	<0.001
Antipsychotics	7,911 (8.9)	11,368 (13.8)	0.154	6,273 (11.2)	6,230 (11.1)	0.002
Metformin	7,479 (8.4)	24,605 (29.8)	0.565	7,463 (13.3)	7,689 (13.7)	0.012
Antiparasitic products, insecticides and repellents	6,999 (7.9)	6,529 (7.9)	0.001	4,549 (8.1)	4,550 (8.1)	<0.001
COVID-19 vaccine	6,543 (7.4)	6,402 (7.8)	0.015	4,276 (7.6)	4,262 (7.6)	<0.001
Antineoplastic agents	5,641 (6.3)	5,054 (6.1)	0.009	3,370 (6.0)	3,426 (6.1)	0.004
Antimigraine preparations	5,309 (6.0)	4,429 (5.4)	0.026	3,022 (5.4)	3,099 (5.5)	0.006
Immunosuppressants	2,714 (3.1)	2,441 (3.0)	0.006	1,579 (2.8)	1,640 (2.9)	0.007
Drugs used in addictive disorders	2,644 (3.0)	3,166 (3.8)	0.047	1,775 (3.2)	1,781 (3.2)	<0.001
Non-selective monoamine reuptake inhibitors	2,165 (2.4)	2,077 (2.5)	0.005	1,358 (2.4)	1,346 (2.4)	0.001
Anti-Parkinson drugs	1,729 (1.9)	2,293 (2.8)	0.055	1,200 (2.1)	1,256 (2.2)	0.007
Aromatase inhibitors	724 (0.8)	1,621 (2.0)	0.098	676 (1.2)	699 (1.2)	0.004
Cardiovascular agents,other	830 (0.9)	844 (1.0)	0.009	538 (1.0)	537 (1.0)	<0.001
Bisphosphonates	399 (0.4)	522 (0.6)	0.025	278 (0.5)	266 (0.5)	0.003
Alendronate	301 (0.3)	395 (0.5)	0.022	209 (0.4)	190 (0.3)	0.006
Other drugs affecting bone structure and mineralization	82 (0.1)	129 (0.2)	0.018	68 (0.1)	73 (0.1)	0.003
Denosumab	70 (0.1)	113 (0.1)	0.018	57 (0.1)	64 (0.1)	0.004
Zoledronic acid	64 (0.1)	75 (0.1)	0.007	43 (0.1)	48 (0.1)	0.003
Ibandronate	40 (0.0)	53 (0.1)	0.008	28 (0.1)	31 (0.1)	0.002
Romosozumab	≤10 (0.0)	≤10 (0.0)	<0.001	≤10 (0.0)	≤10 (0.0)	<0.001
Risedronic acid	≤10 (0.0)	N/A	N/A	N/A	N/A	N/A
Teriparatide	11 (0.0)	≤10 (0.0)	<0.001	≤10 (0.0)	≤10 (0.0)	<0.001
Abaloparatide	≤10 (0.0)	≤10 (0.0)	<0.001	≤10 (0.0)	≤10 (0.0)	<0.001
**Laboratory**						
**BMI**						
Mean ± SD, kg/m^2	37.2 ± 7.3	35.7 ± 7.0		37.3 ± 7.3	36.0 ± 7.0	
≥30 kg/m^2	71,354 (80.3)	64,078 (77.6)	0.067	44,898 (79.9)	44,725 (79.5)	0.008
**SBP**						
Mean ± SD, mm[hg]	127.7 ± 15.9	127.4 ± 17.5		127.8 ± 16.2	126.9 ± 17.2	
≥140 mm[hg]	39,565 (44.5)	34,393 (41.7)	0.058	24,146 (42.9)	24,055 (42.8)	0.003
**eGFR**						
Mean ± SD, ml/min/(1.73_m^2)	86.2 ± 21.8	90.6 ± 25.9		87.3 ± 22.8	90.7 ± 25.2	
≥60 ml/min/(1.73_m^2)	62,543 (70.4)	53,331 (64.6)	0.124	37,531 (66.8)	37,812 (67.3)	0.011
**TG**						
Mean ± SD, mg/dl	133.9 ± 80.9	144.8 ± 93.1		132.9 ± 80.9	144.2 ± 92.8	
≥200 mg/dl	10,576 (11.9)	9,042 (11.0)	0.030	6,211 (11.0)	6,395 (11.4)	0.010
**LDL-C**						
Mean ± SD, mg/dl	109.7 ± 34.5	106.6 ± 35.6		108.2 ± 34.0	108.2 ± 35.6	
≥190 mg/dl	1,606 (1.8)	1,077 (1.3)	0.041	811 (1.4)	847 (1.5)	0.005

**HbA1C**						
Mean ± SD, %	5.5 ± 0.6	5.9 ± 0.9		5.6 ± 0.7	5.8 ± 0.7	
≥7%	792 (0.9)	2,489 (3.0)	0.154	770 (1.4)	891 (1.6)	0.018

Data are presented as mean ± SD for continuous variables and n (%) for categorical variables. SMD, standardized mean difference. A post-matching SMD <0.10 indicates adequate balance. Matching was performed using 1:1 nearest-neighbor propensity score matching with a caliper of 0.2 SD of the logit propensity score based on 215 covariates. SMD, standardized mean difference; COVID-19, coronavirus disease 2019; BMI, body mass index; SBP, systolic blood pressure; eGFR, estimated glomerular filtration rate; TG, triglycerides; LDL-C, low-density lipoprotein cholesterol; HbA1c, glycated hemoglobin A1c.

Supplementary Table 11. Per-Protocol Hazard Ratios for Skeletal Outcomes Across Cumulative 75-Day Refill Intervals.

Obesity with Type 2 Diabetes

	Semaglutide vs Empagliflozin		Semaglutide vs Sitagliptin		Semaglutide vs Glipizide		Semaglutide vs Usual care	
Outcome	HR (95% CI)	*p* value	HR (95% CI)	*p* value	HR (95% CI)	*p* value	HR (95% CI)	*p* value
**Persistence exposed through day 75 (1 interval)**								
MOF	0.59 (0.49, 0.71)	<0.001	0.69 (0.52, 0.91)	0.008	0.58 (0.47, 0.72)	<0.001	0.69 (0.61, 0.79)	<0.001
Osteoporosis	0.93 (0.80, 1.09)	0.400	1.01 (0.80, 1.28)	0.913	0.92 (0.76, 1.11)	0.388	0.89 (0.80, 0.99)	0.030
Osteoarthritis of knee	1.19 (1.06, 1.32)	0.002	1.20 (1.01, 1.42)	0.039	1.13 (0.99, 1.29)	0.077	1.01 (0.94, 1.09)	0.716
Osteoarthritis of hip	1.02 (0.88, 1.18)	0.796	1.10 (0.87, 1.39)	0.432	0.99 (0.83, 1.19)	0.959	0.97 (0.88, 1.07)	0.495
Gout	0.78 (0.66, 0.93)	0.007	0.90 (0.66, 1.23)	0.523	0.84 (0.66, 1.06)	0.138	0.88 (0.77, 1.01)	0.065
**Persistence exposed through day 150 (2 consecutive intervals)**								
MOF	0.55 (0.42, 0.71)	<0.001	0.60 (0.39, 0.94)	0.022	0.77 (0.56, 1.04)	0.089	0.74 (0.62, 0.87)	<0.001
Osteoporosis	0.79 (0.64, 0.97)	0.026	0.91 (0.65, 1.28)	0.590	0.97 (0.74, 1.28)	0.847	0.88 (0.78, 1.00)	0.057
Osteoarthritis of knee	1.25 (1.08, 1.45)	0.003	1.07 (0.84, 1.35)	0.604	1.10 (0.91, 1.32)	0.336	0.93 (0.85, 1.01)	0.095
Osteoarthritis of hip	0.99 (0.81, 1.20)	0.912	1.01 (0.73, 1.39)	0.964	1.05 (0.81, 1.35)	0.731	0.96 (0.85, 1.08)	0.465
Gout	0.66 (0.52, 0.84)	<0.001	0.90 (0.58, 1.41)	0.645	0.90 (0.65, 1.25)	0.541	0.87 (0.74, 1.02)	0.091
**Persistence exposed through day 225 (3 consecutive intervals)**								
MOF	0.58 (0.43, 0.77)	<0.001	0.73 (0.46, 1.16)	0.183	0.69 (0.47, 1.00)	0.048	0.74 (0.61, 0.90)	0.002
Osteoporosis	0.91 (0.71, 1.15)	0.423	0.81 (0.56, 1.17)	0.260	1.12 (0.83, 1.53)	0.452	0.84 (0.72, 0.97)	0.017
Osteoarthritis of knee	1.24 (1.04, 1.47)	0.014	0.98 (0.74, 1.28)	0.857	0.99 (0.80, 1.23)	0.912	0.94 (0.85, 1.04)	0.209
Osteoarthritis of hip	0.99 (0.80, 1.22)	0.915	0.91 (0.63, 1.32)	0.637	0.89 (0.66, 1.19)	0.426	1.02 (0.89, 1.17)	0.772
Gout	0.74 (0.56, 0.97)	0.026	1.04 (0.63, 1.72)	0.864	0.85 (0.59, 1.22)	0.376	0.93 (0.77, 1.12)	0.447

Obesity without Type 2 Diabetes								
	Semaglutide vs Naltrexone/bupropion		Semaglutide vs Phentermine/topiramate		Semaglutide vs Phentermine		Semaglutide vs Usual care	
Outcome	HR (95% CI)	*p* value	HR (95% CI)	*p* value	HR (95% CI)	*p* value	HR (95% CI)	*p* value
**Persistence exposed through day 75 (1 interval)**								
MOF	N/A	N/A	N/A	N/A	0.78 (0.49, 1.23)	0.282	0.78 (0.50, 1.20)	0.25
Osteoporosis	N/A	N/A	0.67 (0.34, 1.33)	0.251	0.99 (0.71, 1.38)	0.956	1.07 (0.78, 1.47)	0.684
Osteoarthritis of knee	0.99 (0.67, 1.49)	0.984	1.03 (0.70, 1.52)	0.872	1.09 (0.92, 1.30)	0.33	1.14 (0.94, 1.37)	0.173
Osteoarthritis of hip	1.29 (0.71, 2.33)	0.402	0.79 (0.40, 1.56)	0.496	0.73 (0.54, 0.98)	0.036	1.34 (0.99, 1.82)	0.061
Gout	N/A	N/A	N/A	N/A	1.04 (0.61, 1.77)	0.894	0.92 (0.58, 1.46)	0.717
**Persistence exposed through day 150 (2 consecutive intervals)**								
MOF	N/A	N/A	N/A	N/A	0.85 (0.49, 1.48)	0.57	1.13 (0.55, 2.32)	0.731
Osteoporosis	N/A	N/A	N/A	N/A	0.82 (0.54, 1.24)	0.346	1.54 (0.98, 2.41)	0.061
Osteoarthritis of knee	0.83 (0.44, 1.57)	0.573	1.33 (0.78, 2.27)	0.293	0.93 (0.75, 1.16)	0.537	1.10 (0.85, 1.41)	0.475
Osteoarthritis of hip	N/A	N/A	N/A	N/A	0.64 (0.44, 0.95)	0.024	0.86 (0.56, 1.32)	0.489
Gout	N/A	N/A	N/A	N/A	1.43 (0.78, 2.61)	0.25	0.86 (0.48, 1.55)	0.618
**Persistence exposed through day 225 (3 consecutive intervals)**								
MOF	N/A	N/A	N/A	N/A	1.09 (0.56, 2.11)	0.807	N/A	N/A
Osteoporosis	N/A	N/A	N/A	N/A	1.12 (0.71, 1.76)	0.633	1.69 (0.99, 2.87)	0.051
Osteoarthritis of knee	0.99 (0.48, 2.02)	0.973	1.18 (0.63, 2.22)	0.604	0.88 (0.68, 1.13)	0.32	1.22 (0.90, 1.65)	0.208
Osteoarthritis of hip	N/A	N/A	N/A	N/A	0.74 (0.48, 1.14)	0.165	1.22 (0.75, 1.99)	0.412
Gout	N/A	N/A	N/A	N/A	N/A	N/A	1.40 (0.74, 2.67)	0.301

Per-protocol analysis restricted to patients persistent on assigned treatment at each cumulative threshold. The 75-day interval reflects a 30-day prescription plus a 45-day permissible refill gap. HR, hazard ratio; CI, confidence interval; MOF, major osteoporotic fracture; T2D, type 2 diabetes

Supplementary Table 12. Treatment Persistence Rates Across Cumulative 75-Day Refill Intervals by Treatment Group.

Obesity with Type 2 Diabetes						
	Through day 75		Through day 150 among those persistent through day 75		Through day 225 among those persistent through day 150	
	Persistent	Not persistent	Persistent	Not persistent	Persistent	Not persistent
Tirzepatide	41,275 (66.3%)	20,958 (33.7%)	29,915 (48.1%)	11,360 (18.3%)	22,801 (36.6%)	7,114 (11.4%)
Semaglutide	53,129 (54.7%)	43,918 (45.3%)	36,422 (37.5%)	16,707 (17.2%)	28,064 (28.9%)	8,358 (8.6%)
Empagliflozin	26,009 (41.8%)	36,235 (58.2%)	10,242 (16.5%)	15,767 (25.3%)	7,067 (11.4%)	3,175 (5.1%)
Sitagliptin	8,167 (38.1%)	13,281 (61.9%)	3,529 (16.5%)	4,638 (21.6%)	2,638 (12.3%)	891 (4.2%)
Glipizide	15,183 (40.3%)	22,452 (59.7%)	5,809 (15.4%)	9,374 (24.9%)	4,187 (11.1%)	1,622 (4.3%)
Usual care	105,085 (46.1%)	123,107 (53.9%)	52,947 (23.2%)	52,138 (22.8%)	40,935 (17.9%)	12,012 (5.3%)

Obesity without Type 2 Diabetes						
	Through day 75		Through day 150 among those persistent through day 75		Through day 225 among those persistent through day 150	
	Persistent	Not persistent	Persistent	Not persistent	Persistent	Not persistent
Tirzepatide	71,317 (55.3%)	57,744 (44.7%)	46,064 (35.7%)	25,253 (19.6%)	32,146 (24.9%)	13,918 (10.8%)
Semaglutide	44,123 (49.7%)	44,712 (50.3%)	27,682 (31.2%)	16,441 (18.5%)	19,527 (22.0%)	8,155 (9.2%)
Naltrexone/bupropion	3,438 (28.8%)	8,499 (71.2%)	1,174 (9.8%)	2,264 (19.0%)	675 (5.7%)	499 (4.2%)
Phentermine/topiramate	3,775 (34.5%)	7,156 (65.5%)	2,000 (18.3%)	1,775 (16.2%)	1,325 (12.1%)	675 (6.2%)
Phentermine	18,086 (48.1%)	19,506 (51.9%)	10,115 (26.9%)	7,971 (21.2%)	6,318 (16.8%)	3,797 (10.1%)
Usual care	25,908 (31.4%)	56,669 (68.6%)	12,314 (14.9%)	13,594 (16.5%)	9,087 (11.0%)	3,227 (3.9%)

Persistence was defined as continuous medication use without a refill gap exceeding 75 days (30-day prescription plus 45-day permissible gap). The proportion of patients remaining on the assigned treatment is reported at each cumulative threshold (day 75, 150, 225, 300). T2D, type 2 diabetes.

Supplementary Table 13. Subgroup Analysis of Osteoporosis Incidence During Three-Year Follow-Up: Semaglutide Versus Comparators in Obesity With Type 2 Diabetes.

Osteoporosis	Empagliflozin		Sitagliptin		Glipizide		Usual care	
	HR (95%CI)	P for interaction	HR (95%CI)	P for interaction	HR (95%CI)	P for interaction	HR (95%CI)	P for interaction
eGFR, mL/min/1.73m²		0.19		0.13		0.19		0.32
≥45	0.72 (0.53-0.98)		0.86 (0.59-1.27)		0.90 (0.67-1.22)		0.76 (0.64-0.90)	
<45	1.22 (0.59-2.51)		1.73 (0.76-3.92)		1.60 (0.72-3.55)		0.66 (0.53-0.82)	
HbA1c, %		0.03		0.13		0.83		0.20
≥7	1.05 (0.68-1.64)		0.75 (0.42-1.34)		1.07 (0.72-1.58)		0.76 (0.54-1.07)	
<7	0.46 (0.24-0.85)		1.46 (0.76-2.81)		0.99 (0.59-1.67)		0.58 (0.45-0.74)	
Hypertension		0.10		0.32		0.27		0.17
HTN (＋)	0.88 (0.68-1.14)		1.03 (0.77-1.38)		1.03 (0.82-1.30)		0.80 (0.67-0.95)	
HTN (－)	0.24 (0.05-1.13)		0.55 (0.16-1.85)		0.56 (0.20-1.60)		0.54 (0.32-0.91)	
Ischemic heart disease		0.85		0.89		0.51		0.84
IHD (＋)	0.87 (0.60-1.27)		1.02 (0.67-1.57)		1.01 (0.66-1.56)		0.86 (0.63-1.17)	
IHD (－)	0.91 (0.64-1.31)		1.07 (0.70-1.65)		0.84 (0.61-1.17)		0.82 (0.67-1.02)	
Heart failure		0.98		0.79		0.97		0.50
HF (＋)	0.88(0.52-1.49)		0.88 (0.52-1.49)		0.87 (0.48-1.56)		0.61 (0.39-0.96)	
HF (－)	0.87(0.64-1.19)		0.96 (0.69-1.32)		0.85 (0.66-1.10)		0.73 (0.61-0.87)	
Proteinuria		0.07		0.17		0.07		0.02
Proteinuria (＋)	0.40 (0.18-0.86)		0.51 (0.20-1.29)		0.52 (0.27-1.01)		0.40 (0.23-0.69)	
Proteinuria (－)	0.84 (0.64-1.09)		1.00 (0.75-1.34)		0.99 (0.78-1.27)		0.77 (0.65-0.92)	
Metformin		0.82		0.99		0.79		0.19
Metformin (＋)	0.82 (0.60-1.12)		1.15 (0.85-1.57)		0.99 (0.75-1.30)		0.66 (0.54-0.81)	
Metformin (－)	0.87 (0.55-1.36)		1.15 (0.68-1.92)		0.92 (0.59-1.42)		0.84 (0.62-1.14)	
Insulin		0.15		0.49		0.48		0.76
Insulin (＋)	0.64 (0.42-0.97)		0.96 (0.64-1.43)		0.88 (0.61-1.27)		0.79 (0.58-1.08)	
Insulin (－)	0.99 (0.65-1.50)		1.22 (0.70-2.15)		1.07 (0.71-1.61)		0.75 (0.60-0.93)	
ACEi/ARBs		0.45		0.67		0.23		0.48
ACEi/ARBs (＋)	0.94 (0.45-1.95)		0.93 (0.43-2.02)		0.89 (0.48-1.67)		0.67 (0.43-1.05)	
ACEi/ARBs (－)	0.65 (0.37-1.15)		0.74 (0.38-1.47)		0.55 (0.33-0.91)		0.84 (0.53-1.34)	

Subgroup analyses were stratified by eGFR (≥45 vs <45 mL/min/1.73 m²), HbA1c (≥7% vs <7%), BMI categories (<30, 30–34.9, 35–39.9, ≥40 kg/m²), cardiovascular comorbidities (hypertension, heart failure, ischemic heart disease), proteinuria, and background therapy (insulin, metformin, renin–angiotensin system inhibitors). HR, hazard ratio; CI, confidence interval. P-values for interaction are reported where applicable.

Supplementary Table 14. Subgroup Analysis of Knee Osteoarthritis Incidence During Three-Year Follow-Up: Semaglutide Versus Comparators in Obesity With Type 2 Diabetes.

Osteoarthritis-knee	Empagliflozin		Sitagliptin		Glipizide		Usual care	
	HR (95%CI)	P for interaction	HR (95%CI)	P for interaction	HR (95%CI)	P for interaction	HR (95%CI)	P for interaction
eGFR, mL/min/1.73m²		0.91		0.62		0.61		0.34
≥45	1.34 (1.16-1.55)		1.12 (0.93-1.35)		1.09 (0.96-1.24)		1.00 (0.92-1.08)	
<45	1.38 (0.84-2.25)		0.98 (0.59-1.61)		0.98 (0.65-1.46)		1.07 (0.96-1.19)	
HbA1c, %		0.44		0.39		0.72		0.35
≥7	1.19 (0.96-1.48)		1.00 (0.75-1.33)		0.97 (0.81-1.16)		1.00 (0.85-1.17)	
<7	1.02 (0.74-1.41)		0.81 (0.56-1.19)		0.92 (0.71-1.19)		0.91 (0.80-1.02)	
Hypertension		0.85		0.64		0.48		0.42
HTN (＋)	1.34 (1.18-1.53)		1.25 (1.07-1.46)		1.12 (1.00-1.25)		1.02 (0.94-1.11)	
HTN (－)	1.41 (0.90-2.21)		1.43 (0.83-2.44)		0.95 (0.62-1.46)		0.93 (0.75-1.15)	
Ischemic heart disease		0.01		0.80		0.79		0.05
IHD (＋)	1.04 (0.84-1.28)		1.13 (0.87-1.48)		1.08 (0.87-1.35)		1.17 (0.98-1.39)	
IHD (－)	1.52 (1.28-1.80)		1.09 (0.90-1.31)		1.05 (0.91-1.20)		0.96 (0.87-1.05)	
Heart failure		0.67		0.97		0.35		0.33
HF (＋)	1.20 (0.90-1.61)		1.20 (0.90-1.61)		1.22 (0.91-1.63)		1.14 (0.89-1.45)	
HF (－)	1.29 (1.11-1.51)		1.20 (1.02-1.40)		1.05 (0.93-1.17)		1.00 (0.92-1.09)	
Proteinuria		0.54		0.07		0.24		0.10
Proteinuria (＋)	1.20 (0.86-1.67)		0.84 (0.57-1.22)		0.93 (0.68-1.26)		0.82 (0.65-1.02)	
Proteinuria (－)	1.34 (1.17-1.53)		1.21 (1.05-1.40)		1.13 (1.01-1.26)		1.00 (0.92-1.08)	
Metformin		0.99		0.36		0.92		0.98
Metformin (＋)	1.40 (1.20-1.62)		1.08 (0.92-1.27)		1.12 (0.99-1.26)		1.02 (0.94-1.12)	
Metformin (－)	1.39 (1.08-1.79)		1.26 (0.95-1.66)		1.13 (0.90-1.42)		1.02 (0.87-1.19)	
Insulin		0.70		0.75		0.70		0.22
Insulin (＋)	1.27 (1.03-1.58)		1.15 (0.94-1.40)		1.11 (0.93-1.32)		1.07 (0.92-1.24)	
Insulin (－)	1.35 (1.09-1.68)		1.21 (0.91-1.61)		1.05 (0.88-1.26)		0.96 (0.86-1.06)	
ACEi/ARBs		0.84		0.84		0.41		0.60
ACEi/ARBs (＋)	1.11 (0.80-1.56)		1.04 (0.73-1.49)		0.89 (0.67-1.18)		1.01 (0.81-1.25)	
ACEi/ARBs (－)	1.17 (0.87-1.57)		1.10 (0.79-1.52)		1.03 (0.82-1.29)		0.92 (0.70-1.20)	

Subgroup analyses were stratified by eGFR (≥45 vs <45 mL/min/1.73 m²), HbA1c (≥7% vs <7%), BMI categories (<30, 30–34.9, 35–39.9, ≥40 kg/m²), cardiovascular comorbidities (hypertension, heart failure, ischemic heart disease), proteinuria, and background therapy (insulin, metformin, renin–angiotensin system inhibitors). HR, hazard ratio; CI, confidence interval. P-values for interaction are reported where applicable.

Supplementary Table 15. Subgroup Analysis of Hip Osteoarthritis Incidence During Three-Year Follow-Up: Semaglutide Versus Comparators in Obesity With Type 2 Diabetes.

Osteoarthritis-hip	Empagliflozin		Sitagliptin		Glipizide		Usual care	
	HR (95%CI)	P for interaction	HR (95%CI)	P for interaction	HR (95%CI)	P for interaction	HR (95%CI)	P for interaction
eGFR, mL/min/1.73m²		0.75		0.40		0.79		0.05
≥45	1.12 (0.90-1.39)		1.10 (0.84-1.43)		1.09 (0.90-1.34)		0.96 (0.86-1.08)	
<45	1.01 (0.53-1.91)		0.78 (0.37-1.65)		1.00 (0.54-1.86)		0.80 (0.68-0.93)	
HbA1c, %		0.45		0.67		0.52		0.36
≥7	0.92 (0.69-1.24)		0.82 (0.56-1.20)		1.04 (0.80-1.36)		0.89 (0.70-1.12)	
<7	1.12 (0.74-1.70)		0.94 (0.56-1.57)		1.21 (0.83-1.77)		1.02 (0.86-1.20)	
Hypertension		0.49		0.62		0.04		0.50
HTN (＋)	1.09 (0.91-1.31)		0.96 (0.77-1.20)		1.07 (0.90-1.26)		0.95 (0.85-1.08)	
HTN (－)	0.82 (0.38-1.79)		0.77 (0.33-1.81)		2.07 (1.12-3.81)		1.08 (0.77-1.51)	
Ischemic heart disease		0.68		0.02		0.38		0.83
IHD (＋)	0.96 (0.73-1.26)		0.65 (0.46-0.91)		1.32 (0.97-1.79)		0.97 (0.78-1.20)	
IHD (－)	1.04 (0.80-1.34)		1.12 (0.83-1.52)		1.11 (0.90-1.38)		0.99 (0.86-1.15)	
Heart failure		0.38		0.28		0.32		0.08
HF (＋)	0.78 (0.51-1.18)		0.78 (0.51-1.18)		0.79 (0.51-1.22)		0.73 (0.53-1.01)	
HF (－)	0.96 (0.77-1.20)		1.01 (0.81-1.26)		1.00 (0.84-1.19)		0.99 (0.88-1.12)	
Proteinuria		< 0.01		0.06		0.22		< 0.01
Proteinuria (＋)	0.51 (0.31-0.83)		0.51 (0.28-0.93)		0.76 (0.48-1.21)		0.51 (0.36-0.73)	
Proteinuria (－)	1.17 (0.97-1.42)		0.94 (0.76-1.16)		1.04 (0.88-1.24)		1.06 (0.94-1.19)	
Metformin		0.67		0.47		0.22		0.91
Metformin (＋)	1.00 (0.82-1.23)		0.96 (0.78-1.19)		1.16 (0.97-1.39)		0.95 (0.84-1.08)	
Metformin (－)	0.91 (0.63-1.32)		1.14 (0.76-1.71)		0.90 (0.63-1.29)		0.96 (0.77-1.21)	
Insulin		0.13		0.07		0.56		0.89
Insulin (＋)	0.93 (0.70-1.23)		0.79 (0.60-1.04)		1.25 (0.98-1.59)		1.01 (0.83-1.22)	
Insulin (－)	1.29 (0.94-1.77)		1.24 (0.82-1.88)		1.12 (0.86-1.47)		1.02 (0.87-1.20)	
ACEi/ARBs		0.55		0.41		0.26		0.24
ACEi/ARBs (＋)	1.00 (0.65-1.54)		0.95 (0.57-1.59)		0.99 (0.69-1.44)		1.00 (0.75-1.35)	
ACEi/ARBs (－)	0.83 (0.53-1.29)		0.71 (0.45-1.13)		1.35 (0.93-1.96)		1.32 (0.93-1.87)	

Subgroup analyses were stratified by eGFR (≥45 vs <45 mL/min/1.73 m²), HbA1c (≥7% vs <7%), BMI categories (<30, 30–34.9, 35–39.9, ≥40 kg/m²), cardiovascular comorbidities (hypertension, heart failure, ischemic heart disease), proteinuria, and background therapy (insulin, metformin, renin–angiotensin system inhibitors). HR, hazard ratio; CI, confidence interval. P-values for interaction are reported where applicable.

Supplementary Table 16. Subgroup Analysis of Gout Incidence During Three-Year Follow-Up: Semaglutide Versus Comparators in Obesity With Type 2 Diabetes.

Gout	Empagliflozin		Sitagliptin		Glipizide		Usual care	
	HR (95%CI)	P for interaction	HR (95%CI)	P for interaction	HR (95%CI)	P for interaction	HR (95%CI)	P for interaction
eGFR, mL/min/1.73m²		0.11		0.69		0.16		< 0.01
≥45	1.05 (0.80-1.38)		0.94 (0.66-1.34)		0.86 (0.67-1.12)		0.89 (0.75-1.04)	
<45	0.66 (0.40-1.10)		0.81 (0.42-1.54)		0.58 (0.35-0.95)		0.42 (0.35-0.50)	
HbA1c, %		< 0.01		0.13		0.03		0.13
≥7	1.52 (1.05-2.20)		0.95 (0.61-1.49)		0.97 (0.71-1.31)		1.14 (0.86-1.52)	
<7	0.55 (0.32-0.94)		0.53(0.29-0.98)		0.54 (0.35-0.84)		0.85 (0.66-1.09)	
Hypertension		0.33		0.84		0.31		0.40
HTN (＋)	0.87 (0.70-1.09)		0.66 (0.51-0.85)		0.83 (0.69-1.01)		0.89 (0.75-1.04)	
HTN (－)	1.38 (0.56-3.38)		0.73 (0.25-2.11)		0.52 (0.21-1.28)		1.11 (0.67-1.82)	
Ischemic heart disease		0.01		0.56		0.77		0.69
IHD (＋)	0.70 (0.50-0.98)		0.75 (0.51-1.12)		0.75 (0.52-1.08)		0.84 (0.62-1.14)	
IHD (－)	1.27 (0.94-1.71)		0.88 (0.63-1.23)		0.80 (0.62-1.03)		0.90 (0.75-1.09)	
Heart failure		0.18		0.24		0.31		0.89
HF (＋)	0.68 (0.46-1.01)		0.68 (0.46-1.01)		0.74 (0.50-1.09)		0.90 (0.63-1.29)	
HF (－)	0.95 (0.72-1.26)		0.92 (0.69-1.22)		0.93 (0.75-1.16)		0.93 (0.79-1.10)	
Proteinuria		0.19		0.43		0.61		0.21
Proteinuria (＋)	1.17 (0.75-1.84)		0.97 (0.58-1.62)		0.67 (0.45-1.01)		0.73 (0.52-1.02)	
Proteinuria (－)	0.83 (0.66-1.06)		0.77 (0.60-0.99)		0.76 (0.61-0.94)		0.93 (0.78-1.09)	
Metformin		0.17		0.80		0.31		0.32
Metformin (＋)	1.08 (0.83-1.41)		0.76 (0.57-1.02)		0.87 (0.70-1.08)		0.87 (0.73-1.04)	
Metformin (－)	0.77 (0.52-1.15)		0.82 (0.54-1.23)		0.70 (0.48-1.00)		1.03 (0.77-1.37)	
Insulin		0.31		0.84		0.53		0.36
Insulin (＋)	0.87 (0.62-1.23)		0.90 (0.64-1.27)		0.87 (0.64-1.18)		0.85 (0.64-1.12)	
Insulin (－)	1.14 (0.77-1.67)		0.84 (0.50-1.43)		1.01 (0.72-1.42)		1.00 (0.81-1.23)	
ACEi/ARBs		0.92		0.77		0.09		0.61
ACEi/ARBs (＋)	0.93 (0.56-1.56)		0.84 (0.50-1.42)		1.09 (0.69-1.72)		1.07 (0.74-1.56)	
ACEi/ARBs (－)	0.89 (0.51-1.56)		0.95 (0.52-1.71)		0.64 (0.41-0.98)		0.93 (0.61-1.41)	

Subgroup analyses were stratified by eGFR (≥45 vs <45 mL/min/1.73 m²), HbA1c (≥7% vs <7%), BMI categories (<30, 30–34.9, 35–39.9, ≥40 kg/m²), cardiovascular comorbidities (hypertension, heart failure, ischemic heart disease), proteinuria, and background therapy (insulin, metformin, renin–angiotensin system inhibitors). HR, hazard ratio; CI, confidence interval. P-values for interaction are reported where applicable.

Supplementary Table 17. Subgroup Analysis of Osteoporosis Incidence During Two-Year Follow-Up: Semaglutide Versus Comparators in Obesity Without Type 2 Diabetes.

Osteoporosis	Naltrexone-Bupropion		Phentermine		Phentermine-Topiramate		Usual care	
	HR (95%CI)	P for interaction	HR (95%CI)	P for interaction	HR (95%CI)	P for interaction	HR (95%CI)	P for interaction
eGFR, mL/min/1.73m²		0.93		N/A		0.75		0.97
≥45	0.69 (0.40-1.18)		1.29 (0.86-1.94)		1.09 (0.60-1.98)		0.56 (0.42-0.74)	
<45	0.62 (0.06-6.34)		N/A		1.75 (0.10-31.00)		0.59 (0.06-5.56)	
HbA1c, %		N/A		0.78		0.62		0.49
≥7	N/A		1.38 (0.18-10.82)		0.92 (0.08-10.92)		0.89 (0.14-5.57)	
<7	0.79 (0.41-1.53)		1.02 (0.63-1.67)		1.78 (0.88-3.58)		0.46 (0.33-0.64)	
Hypertension		0.65		0.38		0.16		0.18
HTN (＋)	0.63 (0.33-1.20)		1.12 (0.68-1.83)		2.27 (1.10-4.69)		0.42 (0.30-0.60)	
HTN (－)	0.82 (0.34-1.97)		0.77 (0.39-1.52)		1.08 (0.52-2.24)		0.63 (0.40-1.00)	
Ischemic heart disease		0.18		0.35		0.75		0.64
IHD (＋)	0.27 (0.06-1.21)		0.58 (0.15-2.26)		0.85 (0.07-10.33)		0.36 (0.12-1.09)	
IHD (－)	0.80 (0.47-1.36)		1.15 (0.76-1.74)		1.28 (0.73-2.23)		0.48 (0.22-0.69)	
Heart failure		0.30		0.76		0.35		0.68
HF (＋)	2.86 (0.44-18.56)		1.59 (0.23-10.97)		4.22 (0.51-35.22)		0.61 (0.22-1.74)	
HF (－)	1.00 (0.56-1.82)		1.17 (0.78-1.77)		1.46 (0.79-2.69)		0.49 (0.36-0.66)	
Proteinuria		N/A		0.99		N/A		0.76
Proteinuria (＋)	N/A		1.23 (0.08-19.70)		N/A		0.41 (0.09-2.01)	
Proteinuria (－)	0.78 (0.45-1.34)		1.26 (0.87-1.85)		0.97 (0.54-1.74)		0.53 (0.39-0.71)	
Metformin		0.90		0.80		0.06		0.51
Metformin (＋)	0.92 (0.26-3.22)		1.58 (0.59-4.23)		0.32 (0.07-1.53)		0.65 (0.37-1.15)	
Metformin (－)	1.01 (0.51-2.02)		1.36 (0.77-2.40)		1.62 (0.80-3.29)		0.51 (0.33-0.78)	
Insulin		N/A		0.29		N/A		0.91
Insulin (＋)	N/A		0.35 (0.04-3.25)		N/A		0.55 (0.14-2.10)	
Insulin (－)	1.13 (0.69-1.86)		1.19 (0.80-1.79)		1.32 (0.77-2.27)		0.51 (0.38-0.68)	
ACEi/ARBs		0.51		0.25		0.60		0.97
ACEi/ARBs (＋)	0.71 (0.13-3.78)		3.68 (0.64-21.08)		2.89 (0.14-58.58)		0.61 (0.23-1.57)	
ACEi/ARBs (－)	1.30 (0.68-2.50)		1.28 (0.80-2.05)		1.28 (0.70-2.35)		0.59 (0.42-0.85)	

Subgroup analyses were stratified by eGFR (≥45 vs <45 mL/min/1.73 m²), HbA1c (≥7% vs <7%), BMI categories (<30, 30–34.9, 35–39.9, ≥40 kg/m²), cardiovascular comorbidities (hypertension, heart failure, ischemic heart disease), proteinuria, and background therapy (insulin, metformin, renin–angiotensin system inhibitors). HR, hazard ratio; CI, confidence interval. P-values for interaction are reported where applicable.

Supplementary Table 18. Subgroup Analysis of Knee Osteoarthritis Incidence During Two-Year Follow-Up: Semaglutide Versus Comparators in Obesity Without Type 2 Diabetes.

Osteoarthritis-knee	Naltrexone-Bupropion		Phentermine		Phentermine-Topiramate		Usual care	
	HR (95%CI)	P for interaction	HR (95%CI)	P for interaction	HR (95%CI)	P for interaction	HR (95%CI)	P for interaction
eGFR, mL/min/1.73m²		0.72		0.41		0.30		0.19
≥45	1.09 (0.90-1.33)		1.15 (1.01-1.31)		1.19 (1.00-1.42)		1.13 (1.03-1.25)	
<45	0.84 (0.20-3.49)		0.70 (0.21-2.30)		0.50 (0.10-2.56)		0.64 (0.28-1.49)	
HbA1c, %		N/A		0.12		N/A		0.24
≥7	N/A		4.77 (0.84-27.06)		N/A		0.58 (0.22-1.54)	
<7	1.15 (0.92-1.44)		1.21 (1.05-1.41)		1.10 (0.90-1.34)		1.04 (0.94-1.16)	
Hypertension		0.13		0.01		0.81		0.06
HTN (＋)	1.08 (0.86-1.35)		0.98 (0.83-1.15)		1.25 (0.99-1.59)		1.08 (0.96-1.22)	
HTN (－)	1.47 (1.06-2.03)		1.41 (1.14-1.74)		1.20 (0.95-1.52)		1.32 (1.12-1.55)	
Ischemic heart disease		0.82		0.77		0..52		0.38
IHD (＋)	1.17 (0.68-2.00)		1.27 (0.81-2.00)		1.51 (0.84-2.70)		1.29 (0.93-1.78)	
IHD (－)	1.09 (0.90-1.34)		1.19 (1.04-1.35)		1.23 (1.04-1.47)		1.10 (0.98-1.24)	
Heart failure		0.85		0.96		0.05		0.79
HF (＋)	0.87 (0.24-2.24)		1.11 (0.55-2.22)		3.37 (1.08-10.57)		1.06 (0.68-1.63)	
HF (－)	0.95 (0.77-1.18)		1.09 (0.96-1.24)		1.07 (0.87-1.33)		1.12 (1.01-1.25)	
Proteinuria		0.17		0.88		0.08		0.16
Proteinuria (＋)	1.80 (0.62-5.24)		1.04 (0.46-2.35)		3.34 (1.00-11.15)		1.72 (0.92-3.18)	
Proteinuria (－)	0.84 (0.69-1.03)		1.11 (0.98-1.25)		1.14 (0.95-1.37)		1.09 (0.99-1.21)	
Metformin		0.36		0.59		1.00		0.61
Metformin (＋)	0.80 (0.47-1.37)		1.10 (0.80-1.51)		1.17 (0.80-1.71)		1.03 (0.83-1.29)	
Metformin (－)	1.05 (0.83-1.33)		1.22 (1.01-1.46)		1.18 (0.92-1.50)		1.11 (0.95-1.30)	
Insulin		0.12		0.95		0.10		0.81
Insulin (＋)	0.40 (0.13-1.23)		1.12 (0.54-2.34)		2.29 (0.92-5.68)		1.10 (0.64-1.88)	
Insulin (－)	0.98 (0.81-1.19)		1.10 (0.97-1.25)		1.05 (0.88-1.25)		1.03 (0.93-1.14)	
ACEi/ARBs		0.03		0.96		0.58		0.62
ACEi/ARBs (＋)	1.87 (1.02-3.43)		1.20 (0.73-1.97)		1.29 (0.60-2.76)		1.17 (0.81-1.67)	
ACEi/ARBs (－)	0.88 (0.68-1.12)		1.21 (1.04-1.41)		1.03 (0.84-1.27)		1.06 (0.93-1.21)	

Subgroup analyses were stratified by eGFR (≥45 vs <45 mL/min/1.73 m²), HbA1c (≥7% vs <7%), BMI categories (<30, 30–34.9, 35–39.9, ≥40 kg/m²), cardiovascular comorbidities (hypertension, heart failure, ischemic heart disease), proteinuria, and background therapy (insulin, metformin, renin–angiotensin system inhibitors). HR, hazard ratio; CI, confidence interval. P-values for interaction are reported where applicable.

Supplementary Table 19. Subgroup Analysis of Hip Osteoarthritis Incidence During Two-Year Follow-Up: Semaglutide Versus Comparators in Obesity Without Type 2 Diabetes.

Osteoarthritis-hip	Naltrexone-Bupropion		Phentermine		Phentermine-Topiramate		Usual care	
	HR (95%CI)	P for interaction	HR (95%CI)	P for interaction	HR (95%CI)	P for interaction	HR (95%CI)	P for interaction
eGFR, mL/min/1.73m²		N/A		0.90		N/A		0.78
≥45	0.94 (0.66-1.33)		1.71 (0.91-3.20)		1.39 (1.02-1.89)		1.01 (0.86-1.20)	
<45	N/A		1.06 (0.23-4.98)		N/A		0.84 (0.24-2.94)	
HbA1c, %		N/A		0.52		N/A		0.09
≥7	N/A		1.62 (0.38-6.79)		N/A		3.03 (0.73-12.53)	
<7	1.02 (0.69-1.49)		1.00 (0.77-1.31)		1.41 (0.98-2.02)		0.89 (0.74-1.07)	
Hypertension		0.07		< 0.01		0.48		0.05
HTN (＋)	0.87 (0.59-1.30)		1.01 (0.76-1.34)		1.27 (0.83-1.94)		0.92 (0.75-1.12)	
HTN (－)	1.69 (0.95-3.00)		2.04 (1.40-2.97)		1.56 (1.05-2.31)		1.29 (0.98-1.71)	
Ischemic heart disease		0.98		0.90		0.70		0.42
IHD (＋)	1.20 (0.53-2.71)		1.16 (0.50-2.69)		1.58 (0.58-4.27)		0.91 (0.55-1.48)	
IHD (－)	1.21 (0.86-1.70)		1.23 (0.97-1.56)		1.29 (0.95-1.74)		1.13 (0.91-1.40)	
Heart failure		0.52		0.95		0.26		0.16
HF (＋)	0.79 (0.19-3.28)		1.16 (0.39-3.48)		0.43 (0.05-4.08)		0.60 (0.33-1.10)	
HF (－)	1.28 (0.88-1.86)		1.20 (0.95-1.51)		1.60 (1.14-2.25)		0.94 (0.79-1.13)	
Proteinuria		0.40		0.35		0.62		0.25
Proteinuria (＋)	2.58 (0.45-14.94)		2.84 (0.39-20.77)		2.35 (0.36-15.40)		0.45 (0.15-1.41)	
Proteinuria (－)	1.20 (0.86-1.68)		1.09 (0.87-1.37)		1.45 (1.04-2.01)		0.89 (0.75-1.06)	
Metformin		0.62		0.18		0.66		0.53
Metformin (＋)	1.12 (0.48-2.61)		2.01 (1.10-3.67)		1.17 (0.58-2.36)		0.94 (0.64-1.36)	
Metformin (－)	1.42 (0.95-2.13)		1.26 (0.91-1.73)		1.40 (0.95-2.08)		1.08 (0.83-1.41)	
Insulin		0.99		0.52		0.78		0.58
Insulin (＋)	1.31 (0.26-6.47)		0.81 (0.26-2.46)		1.12 (0.35-3.55)		1.18 (0.56-2.49)	
Insulin (－)	1.32 (0.94-1.85)		1.17 (0.94-1.47)		1.32 (0.95-1.83)		0.95 (0.80-1.14)	
ACEi/ARBs		0.50		0.19		0.11		0.29
ACEi/ARBs (＋)	1.00 (0.31-3.17)		0.65 (0.25-1.72)		0.49 (0.13-1.91)		0.67 (0.32-1.41)	
ACEi/ARBs (－)	1.52 (0.98-2.36)		1.28 (0.98-1.68)		1.53 (1.07-2.19)		1.02 (0.82-1.28)	

Subgroup analyses were stratified by eGFR (≥45 vs <45 mL/min/1.73 m²), HbA1c (≥7% vs <7%), BMI categories (<30, 30–34.9, 35–39.9, ≥40 kg/m²), cardiovascular comorbidities (hypertension, heart failure, ischemic heart disease), proteinuria, and background therapy (insulin, metformin, renin–angiotensin system inhibitors). HR, hazard ratio; CI, confidence interval. P-values for interaction are reported where applicable.

Supplementary Table 20. Subgroup Analysis of Gout Incidence During Two-Year Follow-Up: Semaglutide Versus Comparators in Obesity Without Type 2 Diabetes.

Gout	Naltrexone-Bupropion		Phentermine		Phentermine-Topiramate		Usual care	
	HR (95%CI)	P for interaction	HR (95%CI)	P for interaction	HR (95%CI)	P for interaction	HR (95%CI)	P for interaction
eGFR, mL/min/1.73m²		0.30		0.05		0.92		0.06
≥45	0.70 (0.45-1.09)		0.86 (0.62-1.20)		1.08 (0.64-1.81)		0.99 (0.77-1.26)	
<45	2.08 (0.28-15.34)		4.80 (0.91-25.42)		1.19 (0.17-8.41)		2.89 (0.99-8.43)	
HbA1c, %		N/A		N/A		N/A		N/A
≥7	N/A		N/A		N/A		N/A	
<7	0.48 (0.28-0.83)		1.04 (0.70-1.53)		1.00 (0.55-1.83)		0.98 (0.76-1.26)	
Hypertension		0.19		0.02		0.35		0.31
HTN (＋)	0.77 (0.49-1.21)		0.79 (0.55-1.14)		1.03 (0.56-1.90)		1.01 (0.77-1.32)	
HTN (－)	0.35 (0.11-1.06)		1.97 (1.02-3.79)		1.67 (0.75-3.72)		1.33 (0.84-2.11)	
Ischemic heart disease		0.19		0.47		0.95		0.10
IHD (＋)	1.24 (0.47-3.30)		1.66 (0.66-4.18)		1.47 (0.41-5.32)		1.41 (0.77-2.57)	
IHD (－)	0.60 (0.37-0.96)		1.16 (0.84-1.61)		1.41 (0.85-2.32)		0.80 (0.60-1.08)	
Heart failure		N/A		0.94		0.75		0.89
HF (＋)	N/A		1.20 (0.36-3.98)		2.15 (0.51-9.17)		1.04 (0.52-2.09)	
HF (－)	0.56 (0.34-0.91)		1.14 (0.81-1.61)		1.66 (0.87-3.18)		0.98 (0.75-1.29)	
Proteinuria		0.08		N/A		0.81		0.14
Proteinuria (＋)	1.80 (0.42-7.76)		N/A		1.08 (0.15-7.65)		0.48 (0.17-1.34)	
Proteinuria (－)	0.45 (0.28-0.73)		0.98 (0.71-1.35)		1.38 (0.79-2.40)		1.06 (0.82-1.38)	
Metformin		0.14		0.67		0.18		0.53
Metformin (＋)	1.05 (0.31-3.58)		0.89 (0.34-2.32)		3.86 (0.75-19.79)		1.46 (0.75-2.81)	
Metformin (－)	0.37 (0.21-0.68)		1.12 (0.71-1.75)		1.15 (0.57-2.31)		1.14 (0.76-1.70)	
Insulin		N/A		0.99		0.74		0.88
Insulin (＋)	N/A		0.95 (0.15-5.95)		1.92 (0.13-28.12)		0.79 (0.23-2.70)	
Insulin (－)	0.60 (0.39-0.93)		0.94 (0.68-1.30)		1.22 (0.73-2.05)		0.88 (0.69-1.12)	
ACEi/ARBs		0.16		0.86		0.69		0.62
ACEi/ARBs (＋)	1.37 (0.39-4.74)		1.17 (0.45-3.03)		1.03 (0.15-6.96)		0.96 (0.47-1.96)	
ACEi/ARBs (－)	0.48 (0.23-1.02)		1.07 (0.68-1.68)		1.57 (0.79-3.09)		1.17 (0.81-1.70)	

Subgroup analyses were stratified by eGFR (≥45 vs <45 mL/min/1.73 m²), HbA1c (≥7% vs <7%), BMI categories (<30, 30–34.9, 35–39.9, ≥40 kg/m²), cardiovascular comorbidities (hypertension, heart failure, ischemic heart disease), proteinuria, and background therapy (insulin, metformin, renin–angiotensin system inhibitors). HR, hazard ratio; CI, confidence interval. P-values for interaction are reported where applicable

Supplementary Table 21. Individual Fracture Components of Major Osteoporotic Fracture in Obesity with Type 2 Diabetes.

	Exposure Cohort	Comparator Cohort	Risk Difference (95% CI)	ARD (%)	HR-Based RRR (%)	HR (95% CI)	*P* value	Adjusted *P* value (BH Method)	E-value for HR	E-value (CI limit closest to null)
Semaglutide vs Empagliflozin										
**MOF**										
Hip fracture	91/ 47,275 (0.2)	166/ 47,212 (0.4)	-0.002 (-0.002, -0.001)	-0.2	+40.4	0.52 (0.41, 0.68)	<0.001	<0.001	3.23	2.32
Clinical vertebral fracture	212/ 46,938 (0.5)	286/ 46,889 (0.6)	-0.002 (-0.003, -0.001)	-0.2	+22.2	0.71 (0.59, 0.85)	<0.001	<0.001	2.17	1.64
Distal radius-ulna fracture	84/ 47,070 (0.2)	110/ 47,089 (0.2)	-0.001 (-0.001, 0.000)	-0.1	+8.34	0.73 (0.55, 0.97)	0.03	0.039	2.08	1.21
Proximal humerus fracture	107/ 47,121 (0.2)	134/ 47,101 (0.3)	-0.001 (-0.001, 0.000)	-0.1	+26.76	0.77 (0.59, 0.99)	0.039	0.039	1.94	1.13
Semaglutide vs Sitagliptin										
**MOF**										
Hip fracture	44/ 19,740 (0.2)	68/ 19,724 (0.3)	-0.001 (-0.002, -0.000)	-0.1	N/A	0.69 (0.48, 1.02)	0.059	0.118	N/A	N/A
Clinical vertebral fracture	93/ 19,590 (0.5)	152/ 19,645 (0.8)	-0.003 (-0.005, -0.001)	-0.3	31.44	0.66 (0.51, 0.85)	0.001	0.005	2.42	1.64
Distal radius-ulna fracture	33/ 19,692 (0.2)	48/ 19,692 (0.2)	-0.001 (-0.002, 0.000)	-0.1	N/A	0.75 (0.48, 1.17)	0.198	0.264	N/A	N/A
Proximal humerus fracture	56/ 19,690 (0.3)	51/ 19,683 (0.3)	0.000 (-0.001, 0.001)	0	N/A	1.18 (0.80, 1.72)	0.402	0.402	N/A	N/A
Semaglutide vs Glipizide										
**MOF**										
Hip fracture	59/ 32,475 (0.2)	116/ 32,440 (0.4)	-0.002 (-0.003, -0.001)	-0.2	+45.91	0.52 (0.38, 0.71)	<0.001	<0.001	3.24	2.15
Clinical vertebral fracture	144/ 32,254 (0.4)	239/ 32,258 (0.7)	-0.003 (-0.004, -0.002)	-0.3	+40.56	0.62 (0.50, 0.76)	<0.001	<0.001	2.61	1.95
Distal radius-ulna fracture	57/ 32,337 (0.2)	73/ 32,390 (0.2)	-0.000 (-0.001, 0.000)	0	N/A	0.81 (0.57, 1.14)	0.224	0.298	N/A	N/A
Proximal humerus fracture	91/ 32,374 (0.3)	108/ 32,390 (0.3)	-0.001 (-0.001, 0.000)	-0.1	N/A	0.87 (0.65, 1.14)	0.309	0.309	N/A	N/A
Semaglutide vs Usual care										
**MOF**										
Hip fracture	137/ 93,275 (0.1)	163/ 93,254 (0.2)	-0.000 (-0.001, 0.000)	0	N/A	0.89 (0.71, 1.12)	0.333	0.491	N/A	N/A
Clinical vertebral fracture	346/ 92,728 (0.4)	482/ 92,798 (0.5)	-0.001 (-0.002, -0.001)	-0.1	+26.7	0.75 (0.66, 0.87)	<0.001	<0.001	1.98	1.58
Distal radius-ulna fracture	163/ 92,884 (0.2)	191/ 92,913 (0.2)	-0.000 (-0.001, 0.000)	0	N/A	0.91 (0.74, 1.12)	0.368	0.491	N/A	N/A
Proximal humerus fracture	196/ 92,979 (0.2)	199/ 93,038 (0.2)	-0.000 (-0.000, 0.000)	0	N/A	1.03 (0.85, 1.26)	0.736	0.736	N/A	N/A

Site-specific fracture outcomes include hip (S72.0–S72.2), clinical vertebral (S22.0, S32.0), distal radius/ulna (S52.5–S52.6), and proximal humerus (S42.2–S42.3). HR, hazard ratio; CI, confidence interval; T2D, type 2 diabetes. Comparisons are presented for both the obesity with T2D cohort (3-year follow-up) and the obesity without T2D cohort (2-year follow-up).

Supplementary Table 22. Individual Fracture Components of Major Osteoporotic Fracture in Obesity without Type 2 Diabetes.

	Exposure Cohort	Comparator Cohort	Risk Difference (95% CI)	ARD (%)	HR-Based RRR (%)	HR (95% CI)	*P* value	Adjusted *P* value (BH Method)	E-value for HR	E-value (CI limit closest to null)
Semaglutide vs Naltrexone/bupropion										
**MOF**										
Hip fracture	N/A	N/A	N/A	N/A	N/A	N/A	N/A	N/A	N/A	N/A
Clinical vertebral fracture	N/A	N/A	N/A	N/A	N/A	N/A	N/A	N/A	N/A	N/A
Distal radius-ulna fracture	N/A	N/A	N/A	N/A	N/A	N/A	N/A	N/A	N/A	N/A
Proximal humerus fracture	N/A	N/A	N/A	N/A	N/A	N/A	N/A	N/A	N/A	N/A
Semaglutide vs Phentermine/topiramate										
**MOF**										
Hip fracture	N/A	N/A	N/A	N/A	N/A	N/A	N/A	N/A	N/A	N/A
Clinical vertebral fracture	N/A	N/A	N/A	N/A	N/A	N/A	N/A	N/A	N/A	N/A
Distal radius-ulna fracture	11/ 10,762 (0.1)	13/ 10,749 (0.1)	-0.000 (-0.001, 0.001)	0	N/A	0.78 (0.35, 1.74)	0.538	0.538	N/A	N/A
Proximal humerus fracture	N/A	N/A	N/A	N/A	N/A	N/A	N/A	N/A	N/A	N/A
Semaglutide vs Phentermine										
**MOF**										
Hip fracture	N/A	N/A	N/A	N/A	N/A	N/A	N/A	N/A	N/A	N/A
Clinical vertebral fracture	38/ 36,554 (0.1)	41/ 36,532 (0.1)	-0.000 (-0.001, 0.000)	0	N/A	0.97 (0.62, 1.50)	0.880	0.880	N/A	N/A
Distal radius-ulna fracture	38/ 36,450 (0.1)	32/ 36,453 (0.1)	0.000 (-0.000, 0.001)	0	N/A	1.25 (0.78, 2.00)	0.356	0.769	N/A	N/A
Proximal humerus fracture	19/ 36,597 (0.1)	16/ 36,582 (0.0)	0.000 (-0.000, 0.000)	0	N/A	1.25 (0.64, 2.43)	0.513	0.769	N/A	N/A
Semaglutide vs Usual care										
**MOF**										
Hip fracture	N/A	N/A	N/A	N/A	N/A	N/A	N/A	N/A	N/A	N/A
Clinical vertebral fracture	71/ 55,993 (0.1)	70/ 55,976 (0.1)	0.000 (-0.000, 0.000)	0	N/A	0.91 (0.66, 1.27)	0.584	0.755	N/A	N/A
Distal radius-ulna fracture	44/ 55,875 (0.1)	37/ 55,857 (0.1)	0.000 (-0.000, 0.000)	0	N/A	1.07 (0.69, 1.66)	0.755	0.755	N/A	N/A
Proximal humerus fracture	22/ 56,053 (0.0)	25/ 56,065 (0.0)	-0.000 (-0.000, 0.000)	0	N/A	0.80 (0.45, 1.42)	0.449	0.755	N/A	N/A

Site-specific fracture outcomes include hip (S72.0–S72.2), clinical vertebral (S22.0, S32.0), distal radius/ulna (S52.5–S52.6), and proximal humerus (S42.2–S42.3). HR, hazard ratio; CI, confidence interval; T2D, type 2 diabetes. Comparisons are presented for both the obesity with T2D cohort (3-year follow-up) and the obesity without T2D cohort (2-year follow-up).

Supplementary Table 23. Three-Year Hazard Ratios for Skeletal Outcomes Associated With Tirzepatide Versus Comparators in People With Obesity and Type 2 Diabetes.

	Exposure Cohort	Comparator Cohort	Risk Difference (95% CI)	ARD (%)	NNT	HR-Based RRR (%)	HR (95% CI)	*P* value	Adjusted *P* value (BH Method)	E-value for HR	E-value (CI limit closest to null)
Tirzepatide vs Semaglutide											
MOF	271/ 59,684 (0.5)	424/ 59,616 (0.7)	-0.003 (-0.003, -0.002)	-0.3	N/A	N/A	0.91 (0.78, 1.06)	0.220	0.872	N/A	N/A
Osteoporosis	464/ 59,495 (0.8)	639/ 59,312 (1.1)	-0.003 (-0.004, -0.002)	-0.3	N/A	N/A	0.99 (0.88, 1.12)	0.858	0.872	N/A	N/A
Osteoarthritis of knee	1,409/ 54,199 (2.6)	1,878/ 53,924 (3.5)	-0.009 (-0.011, -0.007)	-0.9	N/A	N/A	1.01 (0.94, 1.08)	0.872	0.872	N/A	N/A
Osteoarthritis of hip	619/ 58,555 (1.1)	863/ 58,458 (1.5)	-0.004 (-0.005, -0.003)	-0.4	N/A	N/A	0.98 (0.88, 1.09)	0.723	0.872	N/A	N/A
Gout	403/ 58,817 (0.7)	507/ 58,641 (0.9)	-0.002 (-0.003, -0.001)	-0.2	N/A	N/A	1.06 (0.93, 1.20)	0.422	0.872	N/A	N/A
NCOs	1,686/ 49,869 (3.4)	2,151/ 50,208 (4.3)	-0.009 (-0.011, -0.007)	-0.9	N/A	N/A	1.05 (0.99, 1.12)	0.102	N/A	N/A	N/A
Tirzepatide vs Empagliflozin											
MOF	197/ 35,361 (0.6)	409/ 35,244 (1.2)	-0.006 (-0.007, -0.005)	-0.6	+213	+17.55	0.64 (0.54, 0.76)	<0.001	<0.001	2.49	1.96
Osteoporosis	295/ 35,182 (0.8)	462/ 35,075 (1.3)	-0.005 (-0.006, -0.003)	-0.5	+187	+19.12	0.82 (0.71, 0.95)	0.010	0.017	1.72	1.27
Osteoarthritis of knee	897/ 31,589 (2.8)	1,017/ 32,640 (3.1)	-0.003 (-0.005, -0.000)	-0.3	-53	-28.17	1.18 (1.08, 1.29)	<0.001	<0.001	1.64	1.37
Osteoarthritis of hip	432/ 34,491 (1.3)	541/ 34,716 (1.6)	-0.003 (-0.005, -0.001)	-0.3	N/A	N/A	1.06 (0.93, 1.20)	0.379	0.474	N/A	N/A
Gout	290/ 34,579 (0.8)	361/ 34,517 (1.0)	-0.002 (-0.004, -0.001)	-0.2	N/A	N/A	1.01 (0.87, 1.18)	0.893	0.893	N/A	N/A
NCOs	1,063/ 28,783 (3.7)	1,328/ 29,924 (4.4)	-0.007 (-0.011, -0.004)	-0.7	N/A	N/A	1.07 (0.98, 1.16)	0.122	N/A	N/A	N/A
Tirzepatide vs Sitagliptin											
MOF	102/ 16,500 (0.6)	210/ 16,503 (1.3)	-0.007 (-0.009, -0.004)	-0.7	+803	+4.58	0.76 (0.60, 0.96)	0.023	0.058	1.97	1.24
Osteoporosis	179/ 16,348 (1.1)	304/ 16,289 (1.9)	-0.008 (-0.010, -0.005)	-0.8	N/A	N/A	0.88 (0.73, 1.06)	0.172	0.286	N/A	N/A
Osteoarthritis of knee	437/ 14,592 (3.0)	557/ 15,179 (3.7)	-0.007 (-0.011, -0.003)	-0.7	-57	-22.39	1.21 (1.07, 1.38)	0.003	0.016	1.72	1.33
Osteoarthritis of hip	211/ 16,087 (1.3)	292/ 16,221 (1.8)	-0.005 (-0.008, -0.002)	-0.5	N/A	N/A	1.07 (0.89, 1.28)	0.454	0.567	N/A	N/A
Gout	123/ 16,212 (0.8)	174/ 16,294 (1.1)	-0.003 (-0.005, -0.001)	-0.3	N/A	N/A	1.00 (0.79, 1.26)	0.999	0.999	N/A	N/A
NCOs	470/ 13,528 (3.5)	707/ 13,808 (5.1)	-0.016 (-0.021, -0.012)	-1.6	N/A	N/A	0.99 (0.88, 1.11)	0.806	N/A	N/A	N/A
Tirzepatide vs Glipizide											
MOF	146/ 26,055 (0.6)	345/ 26,071 (1.3)	-0.008 (-0.009, -0.006)	-0.8	+119	+27.06	0.63 (0.52, 0.77)	<0.001	<0.001	2.55	1.93
Osteoporosis	234/ 25,924 (0.9)	353/ 25,951 (1.4)	-0.005 (-0.006, -0.003)	-0.5	N/A	N/A	0.93 (0.79, 1.10)	0.413	0.516	N/A	N/A
Osteoarthritis of knee	652/ 23,380 (2.8)	766/ 24,182 (3.2)	-0.004 (-0.007, -0.001)	-0.4	-73	-18.97	1.23 (1.10, 1.36)	<0.001	<0.001	1.75	1.44
Osteoarthritis of hip	305/ 25,482 (1.2)	439/ 25,709 (1.7)	-0.005 (-0.007, -0.003)	-0.5	N/A	N/A	0.99 (0.85, 1.14)	0.864	0.864	N/A	N/A
Gout	197/ 25,554 (0.8)	291/ 25,695 (1.1)	-0.004 (-0.005, -0.002)	-0.4	N/A	N/A	0.92 (0.77, 1.11)	0.388	0.516	N/A	N/A
NCOs	725/ 21,551 (3.4)	988/ 22,262 (4.4)	-0.011 (-0.014, -0.007)	-1.1	N/A	N/A	1.03 (0.93, 1.13)	0.603	N/A	N/A	N/A
Tirzepatide vs Usual care											
MOF	265/ 59,110 (0.4)	529/ 59,121 (0.9)	-0.004 (-0.005, -0.004)	-0.4	+292	+17.86	0.71 (0.61, 0.82)	<0.001	<0.001	2.18	1.74
Osteoporosis	459/ 58,924 (0.8)	677/ 58,942 (1.1)	-0.004 (-0.005, -0.003)	-0.4	N/A	N/A	0.94 (0.83, 1.06)	0.306	0.382	N/A	N/A
Osteoarthritis of knee	1,389/ 53,714 (2.6)	1,779/ 54,729 (3.3)	-0.007 (-0.009, -0.005)	-0.7	N/A	N/A	1.07 (0.99, 1.15)	0.063	0.128	N/A	N/A
Osteoarthritis of hip	607/ 58,004 (1.0)	812/ 58,247 (1.4)	-0.003 (-0.005, -0.002)	-0.3	N/A	N/A	1.02 (0.92, 1.14)	0.666	0.666	N/A	N/A
Gout	402/ 58,229 (0.7)	488/ 58,557 (0.8)	-0.001 (-0.002, -0.000)	-0.1	N/A	N/A	1.13 (0.99, 1.29)	0.077	0.128	N/A	N/A
NCOs	1,662/ 49,449 (3.4)	2,195/ 50,269 (4.4)	-0.010 (-0.012, -0.008)	-1.0	N/A	N/A	1.03 (0.97, 1.10)	0.358	N/A	N/A	N/A

Data are presented as events/total patients (proportion). Hazard ratios with 95% confidence intervals were estimated using Cox proportional hazards models after 1:1 propensity score matching. Absolute risk differences were derived from Kaplan–Meier cumulative incidence at 1080 days. NNT was computed as 1/|ARD| and rounded upward; reported only for significant results. P-values were adjusted using the Benjamini–Hochberg method. E-values quantify the minimum strength of unmeasured confounding needed to explain away the observed association; reported only for significant results. MOF, major osteoporotic fracture; NCO, negative control outcome; ARD, absolute risk difference; NNT, number needed to treat; RRR, relative risk reduction; HR, hazard ratio; CI, confidence interval; N/A, not applicable.

Supplementary Table 24. Two-Year Hazard Ratios for Skeletal Outcomes Associated With Tirzepatide Versus Comparators in People With Obesity Without Type 2 Diabetes.

	Exposure Cohort	Comparator Cohort	Risk Difference (95% CI)	ARD (%)	NNT	HR-Based RRR (%)	HR (95% CI)	*P* value	Adjusted *P* value (BH Method)	E-value for HR	E-value (CI limit closest to null)
Tirzepatide vs Semaglutide											
MOF	192/ 84,551 (0.2)	252/ 84,631 (0.3)	-0.001 (-0.001, -0.000)	-0.1	N/A	N/A	0.95 (0.79, 1.15)	0.585	0.602	N/A	N/A
Osteoporosis	320/ 84,621 (0.4)	432/ 84,586 (0.5)	-0.001 (-0.002, -0.001)	-0.1	N/A	N/A	0.93 (0.80, 1.07)	0.323	0.601	N/A	N/A
Osteoarthritis of knee	1,116/ 79,161 (1.4)	1,473/ 79,035 (1.9)	-0.005 (-0.006, -0.003)	-0.5	+166	+13.67	0.90 (0.84, 0.98)	0.012	0.059	1.45	1.17
Osteoarthritis of hip	474/ 83,562 (0.6)	602/ 83,638 (0.7)	-0.002 (-0.002, -0.001)	-0.2	N/A	N/A	0.97 (0.86, 1.09)	0.602	0.602	N/A	N/A
Gout	233/ 84,598 (0.3)	263/ 84,582 (0.3)	-0.000 (-0.001, 0.000)	0	N/A	N/A	1.09 (0.91, 1.30)	0.361	0.601	N/A	N/A
NCOs	1,497/ 73,218 (2.0)	1,852/ 73,700 (2.5)	-0.005 (-0.006, -0.003)	-0.5	N/A	N/A	0.99 (0.92, 1.06)	0.781	N/A	N/A	N/A
Tirzepatide vs Naltrexone/bupropion											
MOF	29/ 10,305 (0.3)	34/ 10,319 (0.3)	-0.000 (-0.002, 0.001)	0	N/A	N/A	0.92 (0.56, 1.51)	0.745	0.745	N/A	N/A
Osteoporosis	39/ 10,388 (0.4)	46/ 10,377 (0.4)	-0.001 (-0.002, 0.001)	-0.1	N/A	N/A	0.91 (0.59, 1.39)	0.652	0.745	N/A	N/A
Osteoarthritis of knee	149/ 9,700 (1.5)	155/ 9,720 (1.6)	-0.001 (-0.004, 0.003)	-0.1	N/A	N/A	1.04 (0.83, 1.31)	0.707	0.745	N/A	N/A
Osteoarthritis of hip	49/ 10,252 (0.5)	57/ 10,241 (0.6)	-0.001 (-0.003, 0.001)	-0.1	N/A	N/A	0.92 (0.63, 1.35)	0.676	0.745	N/A	N/A
Gout	25/ 10,404 (0.2)	22/ 10,395 (0.2)	0.000 (-0.001, 0.002)	0	N/A	N/A	1.20 (0.68, 2.13)	0.529	0.745	N/A	N/A
NCOs	188/ 8,800 (2.1)	221/ 8,931 (2.5)	-0.003 (-0.008, 0.001)	-0.3	N/A	N/A	0.93 (0.76, 1.12)	0.434	N/A	N/A	N/A
Tirzepatide vs Phentermine/topiramate											
MOF	19/ 10,647 (0.2)	25/ 10,669 (0.2)	-0.001 (-0.002, 0.001)	-0.1	N/A	N/A	0.89 (0.49, 1.62)	0.702	0.870	N/A	N/A
Osteoporosis	36/ 10,705 (0.3)	43/ 10,695 (0.4)	-0.001 (-0.002, 0.001)	-0.1	N/A	N/A	0.96 (0.62, 1.50)	0.870	0.870	N/A	N/A
Osteoarthritis of knee	134/ 10,162 (1.3)	146/ 10,151 (1.4)	-0.001 (-0.004, 0.002)	-0.1	N/A	N/A	1.06 (0.84, 1.34)	0.623	0.870	N/A	N/A
Osteoarthritis of hip	45/ 10,580 (0.4)	49/ 10,599 (0.5)	-0.000 (-0.002, 0.001)	0	N/A	N/A	1.09 (0.73, 1.63)	0.684	0.870	N/A	N/A
Gout	18/ 10,743 (0.2)	11/ 10,740 (0.1)	0.001 (-0.000, 0.002)	+0.1	N/A	N/A	1.86 (0.88, 3.95)	0.099	0.496	N/A	N/A
NCOs	164/ 9,223 (1.8)	205/ 9,454 (2.2)	-0.004 (-0.008, 0.000)	-0.4	N/A	N/A	0.93 (0.76, 1.15)	0.519	N/A	N/A	N/A
Tirzepatide vs Phentermine											
MOF	71/ 36,883 (0.2)	85/ 36,930 (0.2)	-0.000 (-0.001, 0.000)	0	N/A	N/A	1.06 (0.77, 1.45)	0.740	0.943	N/A	N/A
Osteoporosis	103/ 37,032 (0.3)	140/ 37,003 (0.4)	-0.001 (-0.002, -0.000)	-0.1	N/A	N/A	0.97 (0.75, 1.25)	0.789	0.943	N/A	N/A
Osteoarthritis of knee	397/ 35,300 (1.1)	480/ 35,542 (1.4)	-0.002 (-0.004, -0.001)	-0.2	N/A	N/A	1.06 (0.92, 1.21)	0.422	0.943	N/A	N/A
Osteoarthritis of hip	161/ 36,756 (0.4)	210/ 36,794 (0.6)	-0.001 (-0.002, -0.000)	-0.1	N/A	N/A	0.99 (0.81, 1.22)	0.943	0.943	N/A	N/A
Gout	50/ 37,124 (0.1)	59/ 37,115 (0.2)	-0.000 (-0.001, 0.000)	0	N/A	N/A	1.05 (0.72, 1.53)	0.802	0.943	N/A	N/A
NCOs	552/ 33,087 (1.7)	749/ 33,121 (2.3)	-0.006 (-0.008, -0.004)	-0.6	N/A	N/A	0.93 (0.83, 1.04)	0.198	N/A	N/A	N/A
Tirzepatide vs Usual care											
MOF	115/ 58,640 (0.2)	137/ 58,704 (0.2)	-0.000 (-0.001, 0.000)	0	N/A	N/A	0.95 (0.74, 1.22)	0.698	0.698	N/A	N/A
Osteoporosis	215/ 58,622 (0.4)	220/ 58,697 (0.4)	-0.000 (-0.001, 0.001)	0	N/A	N/A	1.07 (0.89, 1.30)	0.458	0.573	N/A	N/A
Osteoarthritis of knee	693/ 55,401 (1.3)	713/ 56,425 (1.3)	-0.000 (-0.001, 0.001)	0	N/A	N/A	1.08 (0.97, 1.20)	0.141	0.235	N/A	N/A
Osteoarthritis of hip	279/ 58,108 (0.5)	263/ 58,403 (0.5)	0.000 (-0.000, 0.001)	0	-896	-8.48	1.19 (1.00, 1.40)	0.047	0.118	1.66	1.05
Gout	152/ 58,675 (0.3)	116/ 58,842 (0.2)	0.001 (0.000, 0.001)	+0.1	-1540	-12.15	1.44 (1.13, 1.83)	0.003	0.016	2.23	1.51
NCOs	668/ 53,852 (1.2)	705/ 54,652 (1.3)	-0.000 (-0.002, 0.001)	0	N/A	N/A	1.06 (0.95, 1.18)	0.301	N/A	N/A	N/A

Data are presented as events/total patients (proportion). Hazard ratios with 95% confidence intervals were estimated using Cox proportional hazards models after 1:1 propensity score matching. Absolute risk differences were derived from Kaplan–Meier cumulative incidence at 720 days. NNT was computed as 1/|ARD| and rounded upward; reported only for significant results. P-values were adjusted using the Benjamini–Hochberg method. E-values quantify the minimum strength of unmeasured confounding needed to explain away the observed association; reported only for significant results. MOF, major osteoporotic fracture; NCO, negative control outcome; ARD, absolute risk difference; NNT, number needed to treat; RRR, relative risk reduction; HR, hazard ratio; CI, confidence interval; N/A, not applicable.

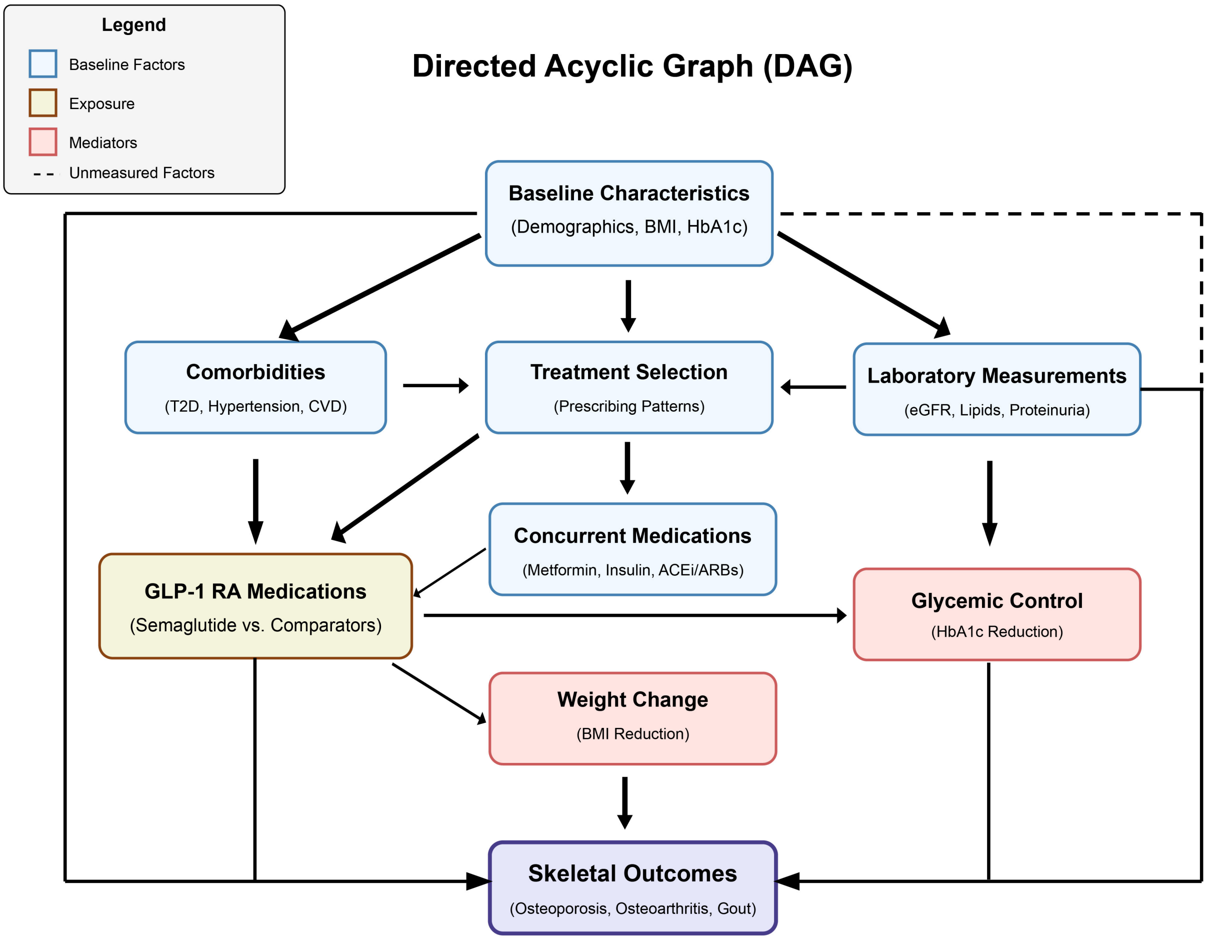

Supplementary Figure 1. Directed Acyclic Graph (DAG) of Causal Pathways Between GLP-1 Receptor Agonist Treatment and Skeletal Health Outcomes in Obesity. This directed acyclic graph depicts hypothesized causal relationships between GLP-1 receptor agonist medications (specifically semaglutide versus comparators) and skeletal outcomes in our target populations. Color-coding differentiates between baseline factors (blue), exposure (yellow), mediators (red), and skeletal outcomes (purple). Baseline characteristics (demographics, BMI, HbA1c) influence comorbidity patterns (T2D, hypertension, CVD) and laboratory measurements (eGFR, lipids, proteinuria), which collectively affect treatment selection decisions. GLP-1 RA treatment influences skeletal outcomes through two primary mediating pathways: weight change (BMI reduction) and glycemic control (HbA1c reduction). The dashed line represents unmeasured factors that may influence both baseline characteristics and skeletal outcomes. Concurrent medications (metformin, insulin, ACEi/ARBs) are positioned as intermediary factors influenced by treatment selection decisions but also directly affecting the principal exposure. This framework guided our propensity score matching approach to minimize confounding and informed our subgroup analyses examining effect modification by comorbidities and laboratory parameters.

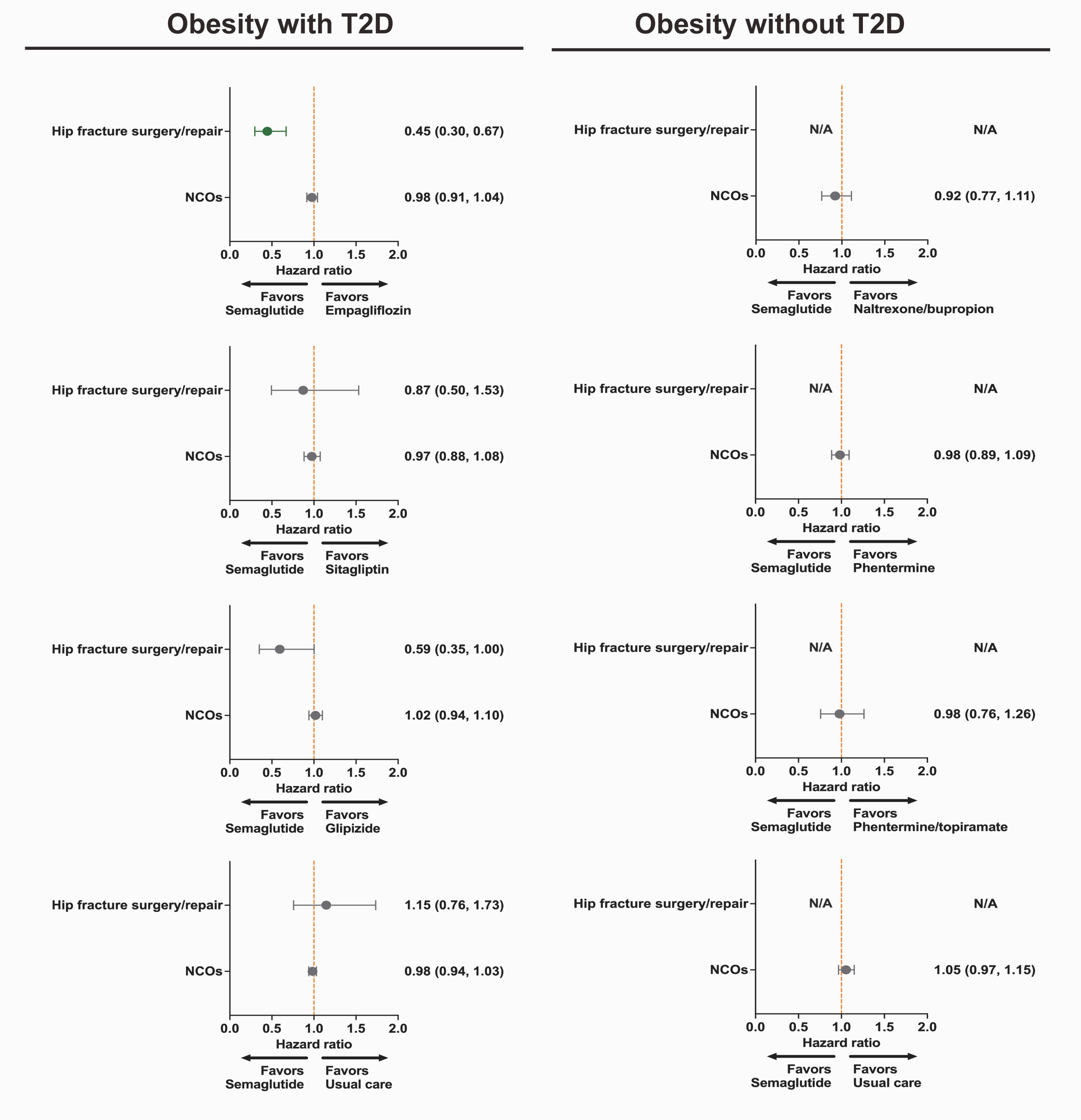

Supplementary Figure 2. Surgery Code–Confirmed Hip Fracture Analysis. Hip fracture was defined by ICD-10 diagnosis codes (S72.0–S72.2) combined with inpatient surgical procedure codes (CPT 27235, 27244, 27245; ICD-10-PCS 0QS6, 0QS7), a validated hard endpoint with a reported positive predictive value exceeding 95% in administrative databases. HR, hazard ratio; CI, confidence interval; T2D, type 2 diabetes.

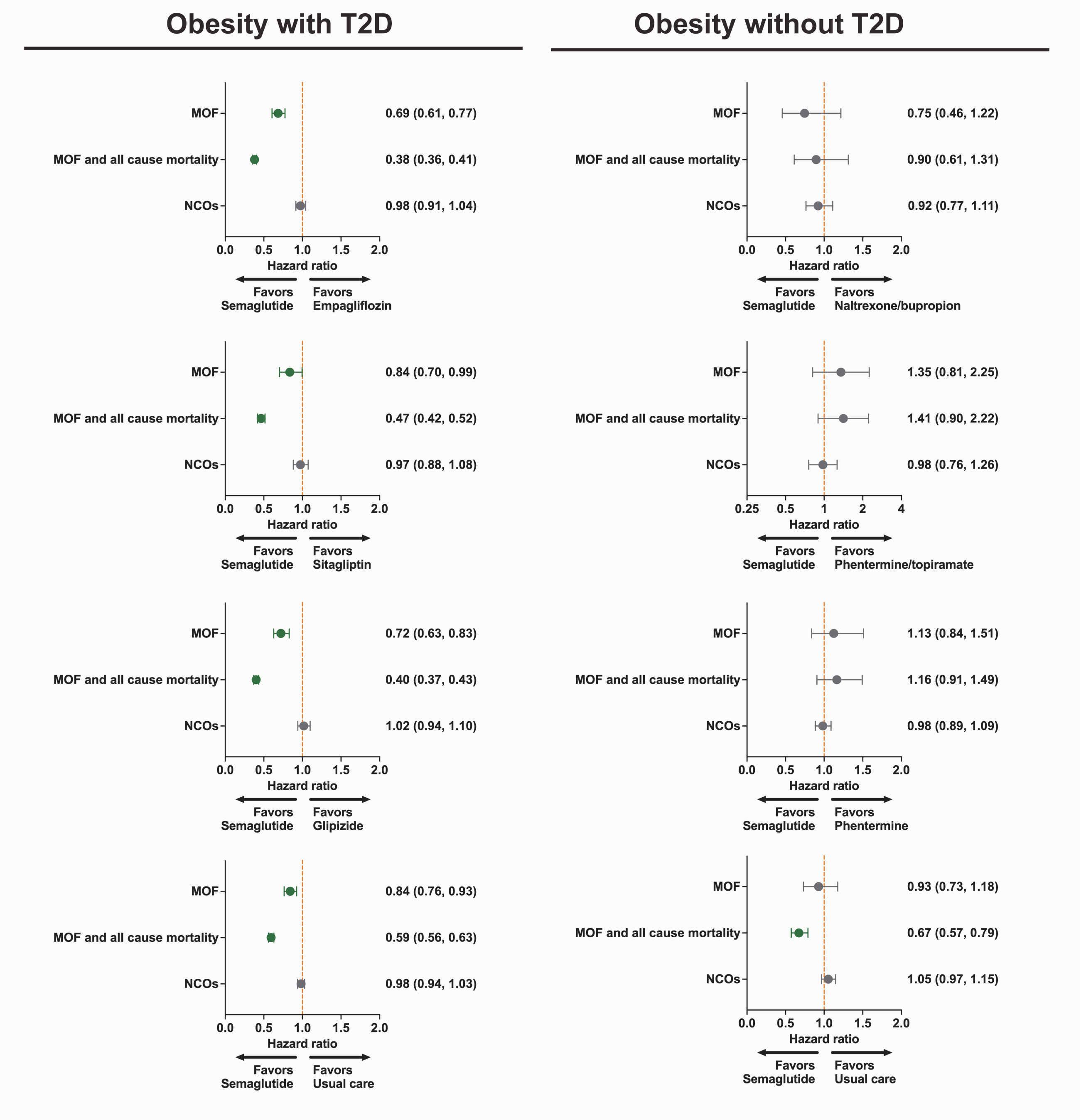

Supplementary Figure 3. Forest plot for the Composite Endpoint of Major Osteoporotic Fracture Plus All-Cause Mortality. This analysis addresses the potential influence of differential mortality as a competing event by combining MOF and all-cause mortality into a single composite endpoint. Comparisons are shown for both the obesity with T2D cohort (3-year follow-up) and the obesity without T2D cohort (2-year follow-up). HR, hazard ratio; CI, confidence interval.

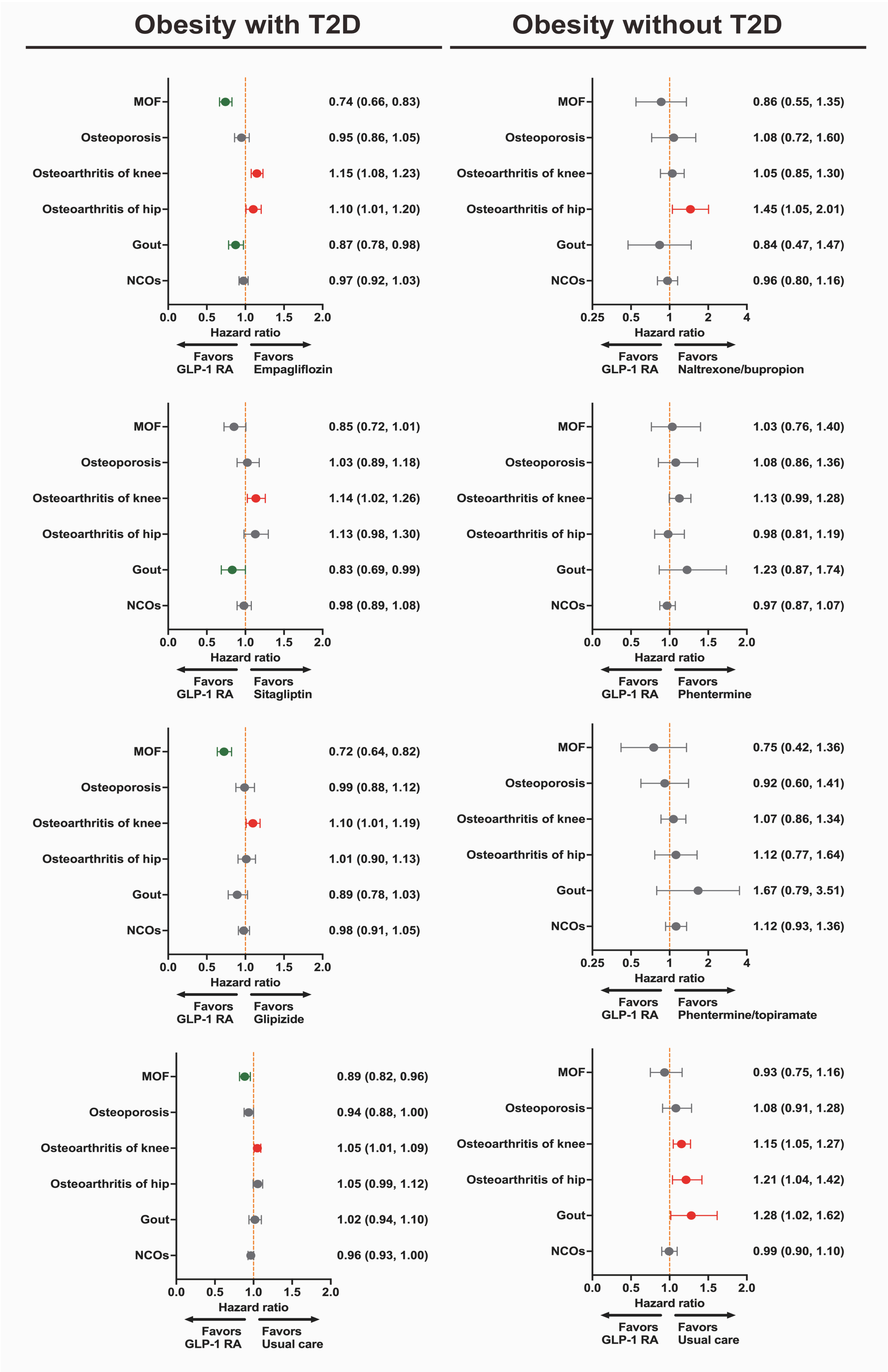

Supplementary Figure 4. GLP-1 Receptor Agonist Class-Level Sensitivity Analysis: Forest Plot of Skeletal Outcomes. In this sensitivity analysis, the exposure was defined as initiation

of any GLP-1 receptor agonist (semaglutide, liraglutide, dulaglutide, or exenatide) rather than semaglutide alone. All other design elements (matching, covariates, outcomes) were identical to the primary analysis. HR, hazard ratio; CI, confidence interval; T2D, type 2 diabetes; MOF, major osteoporotic fracture.

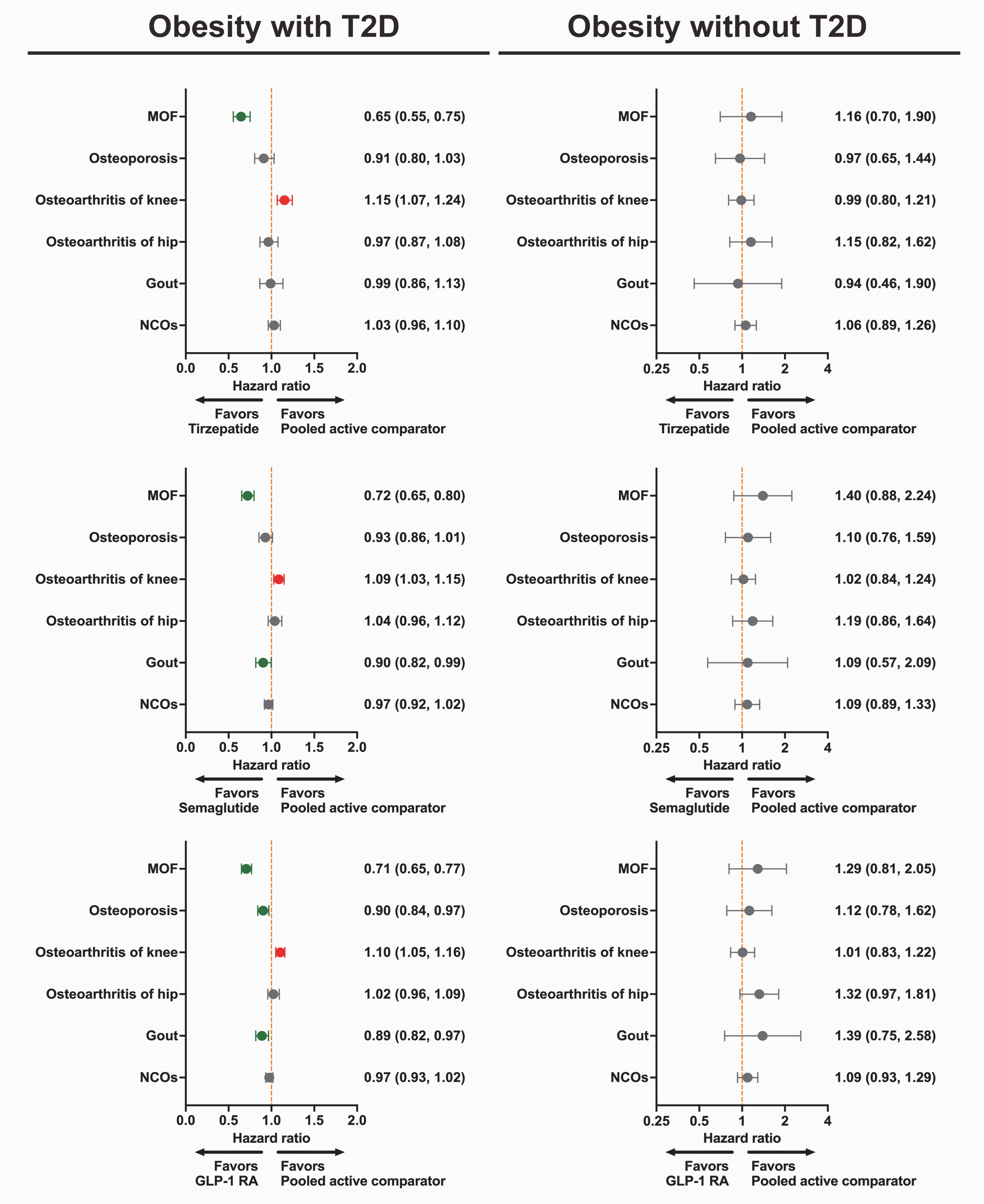

Supplementary Figure 5. Semaglutide Versus Pooled Active Comparators: Forest Plot of Skeletal Outcomes. The pooled active comparator group combined all individual active comparators within each cohort into a single reference group (T2D: empagliflozin + sitagliptin + glipizide; non-T2D: naltrexone–bupropion + phentermine + phentermine–topiramate). HR, hazard ratio; CI, confidence interval; T2D, type 2 diabetes; MOF, major osteoporotic fractur

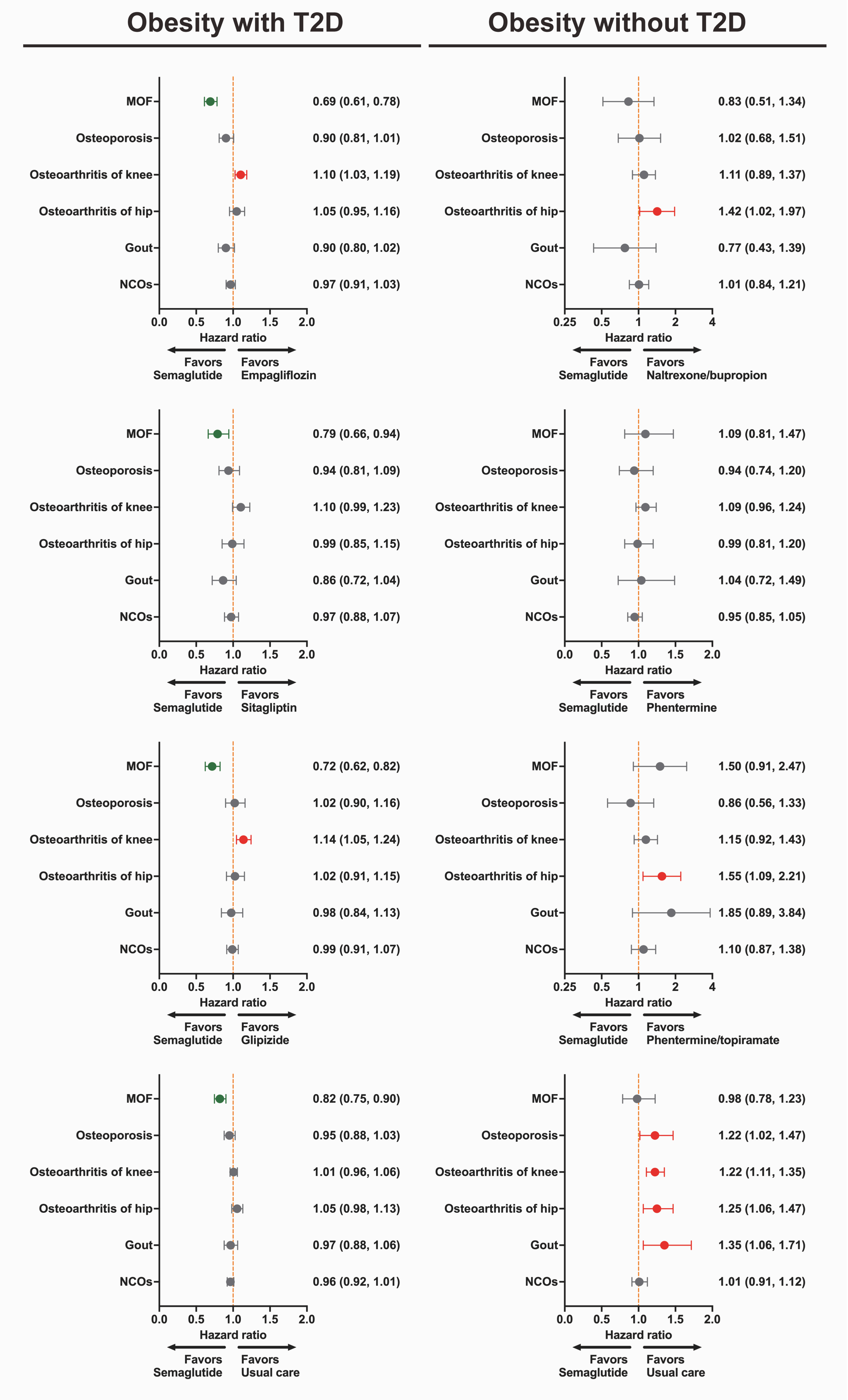
Obesity with T2D
Obesity without T2D
MOF
Osteoporosis
Osteoarthritis of knee
Osteoarthritis of hip
Gout
NCOs
0.69 (0.61, 0.78)
0.90 (0.81, 1.01)
1.10 (1.03, 1.19)
1.05 (0.95, 1.16)
0.90 (0.80, 1.02)
0.97 (0.91, 1.03)
Hazard ratio
Favors Semaglutide
Favors Empagliflozin
0.83 (0.51, 1.34)
1.02 (0.68, 1.51)
1.11 (0.89, 1.37)
1.42 (1.02, 1.97)
0.77 (0.43, 1.39)
1.01 (0.84, 1.21)
Favors Naltrexone/bupropion
0.79 (0.66, 0.94)
0.94 (0.81, 1.09)
1.10 (0.99, 1.23)
0.99 (0.85, 1.15)
0.86 (0.72, 1.04)
0.97 (0.88, 1.07)
Favors Sitagliptin
1.09 (0.81, 1.47)
0.94 (0.74, 1.20)
1.09 (0.96, 1.24)
0.99 (0.81, 1.20)
1.04 (0.72, 1.49)
0.95 (0.85, 1.05)
Favors Phentermine
0.72 (0.62, 0.82)
1.02 (0.90, 1.16)
1.14 (1.05, 1.24)
1.02 (0.91, 1.15)
0.98 (0.84, 1.13)
0.99 (0.91, 1.07)
Favors Glipizide
1.50 (0.91, 2.47)
0.86 (0.56, 1.33)
1.15 (0.92, 1.43)
1.55 (1.09, 2.21)
1.85 (0.89, 3.84)
1.10 (0.87, 1.38)
Favors Phentermine/topiramate
0.82 (0.75, 0.90)
0.95 (0.88, 1.03)
1.01 (0.96, 1.06)
1.05 (0.98, 1.13)
0.97 (0.88, 1.06)
0.96 (0.92, 1.01)
Favors Usual care
0.98 (0.78, 1.23)
1.22 (1.02, 1.47)
1.22 (1.11, 1.35)
1.25 (1.06, 1.47)
1.35 (1.06, 1.71)
1.01 (0.91, 1.12)

Supplementary Figure 6. Global Network Sensitivity Analysis. This sensitivity analysis replicated the primary analysis using the TriNetX Global Collaborative Network (approximately 170 million patients across 80+ healthcare organizations in 30+ countries) to assess generalizability beyond US-based data. All other design elements (matching, covariates, outcomes, follow-up) were identical to the primary analysis. HR, hazard ratio; CI, confidence interval; T2D, type 2 diabetes; MOF, major osteoporotic fracture.

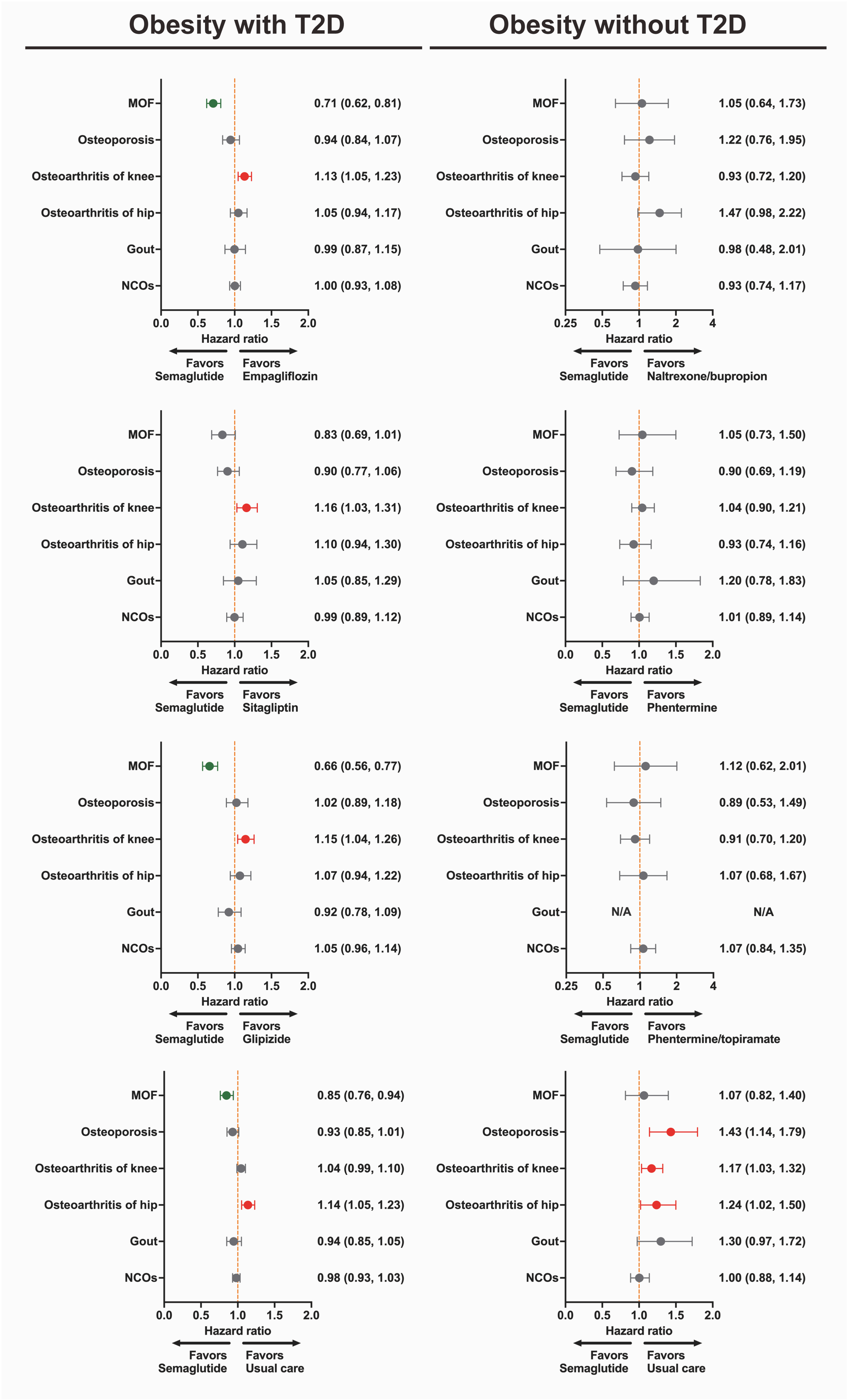
Obesity with T2D
Obesity without T2D
MOF
Osteoporosis
Osteoarthritis of knee
Osteoarthritis of hip
Gout
NCOs
0.71 (0.62, 0.81)
0.94 (0.84, 1.07)
1.13 (1.05, 1.23)
1.05 (0.94, 1.17)
0.99 (0.87, 1.15)
1.00 (0.93, 1.08)
Hazard ratio
Favors Semaglutide
Favors Empagliflozin
1.05 (0.64, 1.73)
1.22 (0.76, 1.95)
0.93 (0.72, 1.20)
1.47 (0.98, 2.22)
0.98 (0.48, 2.01)
0.93 (0.74, 1.17)
Favors Naltrexone/bupropion
0.83 (0.69, 1.01)
0.90 (0.77, 1.06)
1.16 (1.03, 1.31)
1.10 (0.94, 1.30)
1.05 (0.85, 1.29)
0.99 (0.89, 1.12)
Favors Sitagliptin
1.05 (0.73, 1.50)
0.90 (0.69, 1.19)
1.04 (0.90, 1.21)
0.93 (0.74, 1.16)
1.20 (0.78, 1.83)
1.01 (0.89, 1.14)
Favors Phentermine
0.66 (0.56, 0.77)
1.02 (0.89, 1.18)
1.15 (1.04, 1.26)
1.07 (0.94, 1.22)
0.92 (0.78, 1.09)
1.05 (0.96, 1.14)
Favors Glipizide
1.12 (0.62, 2.01)
0.89 (0.53, 1.49)
0.91 (0.70, 1.20)
1.07 (0.68, 1.67)
N/A
N/A
1.07 (0.84, 1.35)
Favors Phentermine/topiramate
0.85 (0.76, 0.94)
0.93 (0.85, 1.01)
1.04 (0.99, 1.10)
1.14 (1.05, 1.23)
0.94 (0.85, 1.05)
0.98 (0.93, 1.03)
Favors Usual care
1.07 (0.82, 1.40)
1.43 (1.14, 1.79)
1.17 (1.03, 1.32)
1.24 (1.02, 1.50)
1.30 (0.97, 1.72)
1.00 (0.88, 1.14)
Favors Usual care

Supplementary Figure 7. Three-Month Landmark Analysis for Skeletal Outcomes Excluding Early Events. In this landmark analysis, patients who experienced an outcome event or were censored within the first 90 days after the index date were excluded. Follow-up was counted from day 91 onward to reduce the influence of prevalent or misclassified events captured shortly after treatment initiation. HR, hazard ratio; CI, confidence interval; T2D, type 2 diabetes; MOF, major osteoporotic fracture.

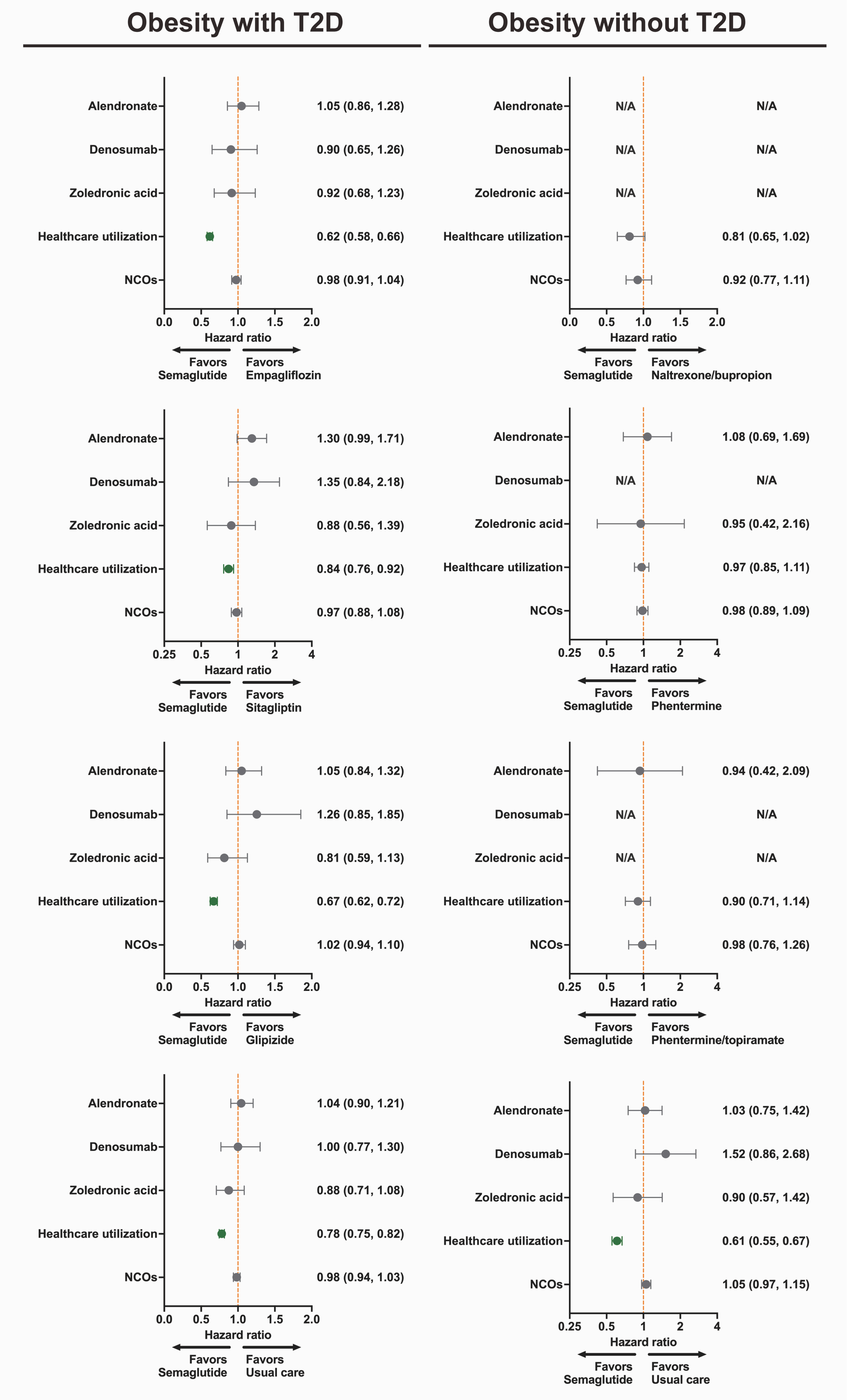

Obesity with T2D
Obesity without T2D
Alendronate
Denosumab
Zoledronic acid
Healthcare utilization
NCOs
1.05 (0.86, 1.28)
0.90 (0.65, 1.26)
0.92 (0.68, 1.23)
0.62 (0.58, 0.66)
0.98 (0.91, 1.04)
Hazard ratio
Favors Semaglutide
Favors Empagliflozin
N/A
0.81 (0.65, 1.02)
0.92 (0.77, 1.11)
Favors Naltrexone/bupropion
1.30 (0.99, 1.71)
1.35 (0.84, 2.18)
0.88 (0.56, 1.39)
0.84 (0.76, 0.92)
0.97 (0.88, 1.08)
Favors Sitagliptin
1.08 (0.69, 1.69)
0.95 (0.42, 2.16)
0.97 (0.85, 1.11)
0.98 (0.89, 1.09)
Favors Phentermine
1.05 (0.84, 1.32)
1.26 (0.85, 1.85)
0.81 (0.59, 1.13)
0.67 (0.62, 0.72)
1.02 (0.94, 1.10)
Favors Glipizide
0.94 (0.42, 2.09)
0.90 (0.71, 1.14)
0.98 (0.76, 1.26)
Favors Phentermine/topiramate
1.04 (0.90, 1.21)
1.00 (0.77, 1.30)
0.88 (0.71, 1.08)
0.78 (0.75, 0.82)
0.98 (0.94, 1.03)
Favors Usual care
1.03 (0.75, 1.42)
1.52 (0.86, 2.68)
0.90 (0.57, 1.42)
0.61 (0.55, 0.67)
1.05 (0.97, 1.15)

Supplementary Figure 8. Bone-Active Medication Use and Healthcare Utilization Across Treatment Comparisons. Bone-active medications include alendronate, zoledronic acid, and denosumab. Healthcare utilization outcomes include outpatient, emergency department, and inpatient encounters. HRs were estimated using Cox proportional hazards models after propensity score matching. HR, hazard ratio; CI, confidence interval; T2D, type 2 diabetes.

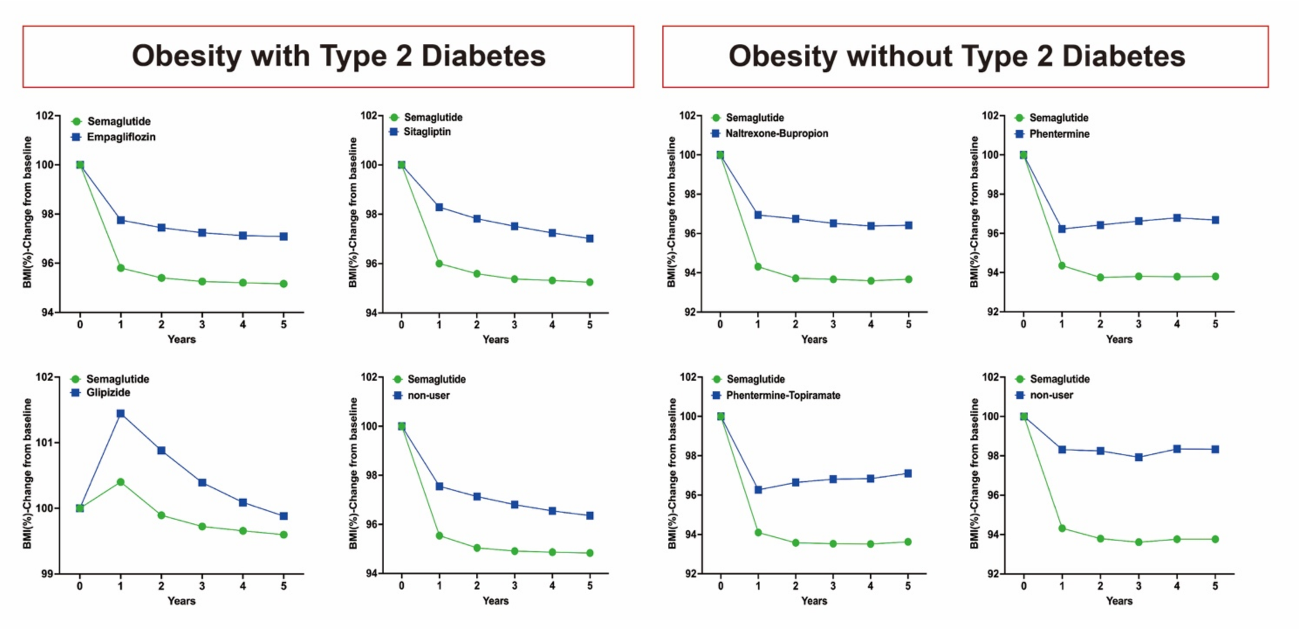

Supplementary Figure 9. Longitudinal Changes in Body Mass Index Among Semaglutide Users Versus Comparator Groups Across Metabolic Phenotypes. This figure illustrates the five-year trajectory of body mass index (BMI) changes (expressed as percentage change from baseline) across treatment groups, stratified by diabetes status. The left panels demonstrate changes in obesity with type 2 diabetes receiving semaglutide versus conventional glucose-lowering medications (empagliflozin, sitagliptin, glipizide, and usual care). The right panels display corresponding changes in obesity without T2D receiving semaglutide versus traditional anti-obesity treatments (naltrexone-bupropion, phentermine, phentermine-topiramate, and non-users). Y-axis values represent percentage changes from baseline, with baseline normalized to 100%.

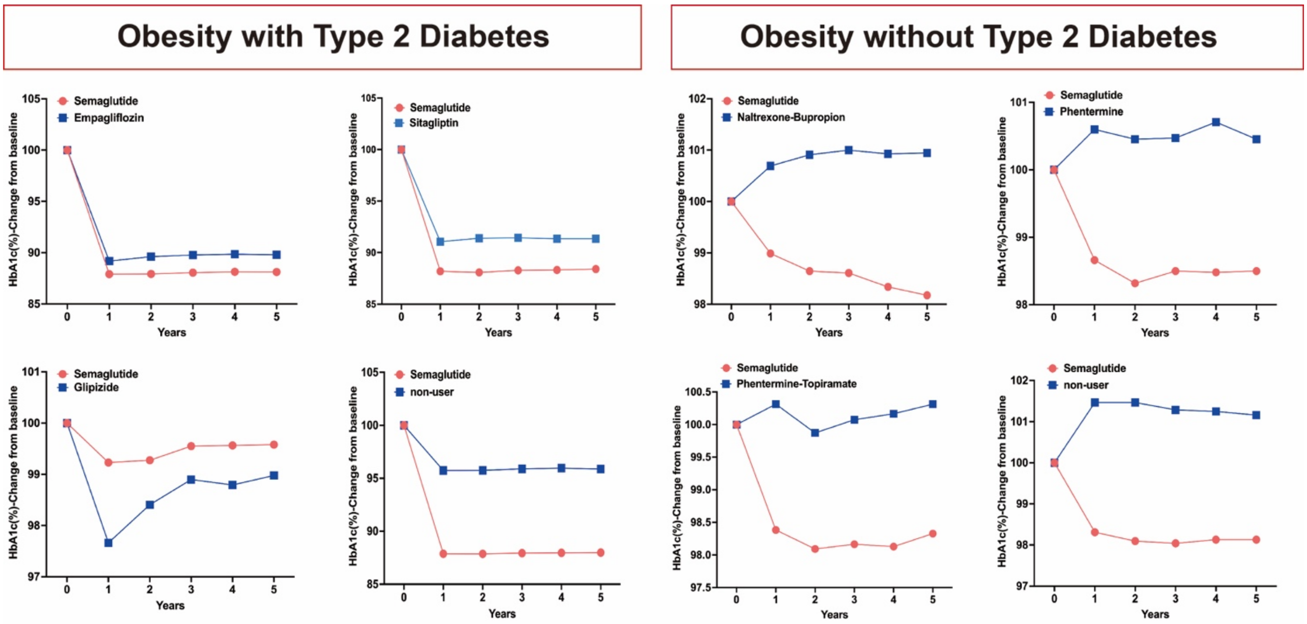

Supplementary Figure 10. Differential Glycemic Control Trajectories with Semaglutide Versus Comparator Therapies in Obesity With and Without Type 2 diabetes. This figure presents longitudinal changes in glycated hemoglobin (HbA1c) over a five-year period, expressed as percentage change from baseline. The left panels show obese individuals with type 2 diabetes receiving semaglutide versus conventional antidiabetic medications (empagliflozin, sitagliptin, glipizide, and usual care). The right panels display non-diabetic obese individuals receiving semaglutide versus alternative anti-obesity treatments (naltrexone-bupropion, phentermine, phentermine-topiramate, and non-users). Y-axis values represent percentage changes from baseline, with baseline normalized to 100%.

References

[1] Gokhale M, Stürmer T, Buse JB. Real-world evidence: the devil is in the detail. Diabetologia. 2020;63:1694-705.
[2] Karim ME, Gustafson P, Petkau J, Tremlett H. Comparison of Statistical Approaches for Dealing With Immortal Time Bias in Drug Effectiveness Studies. Am J Epidemiol. 2016;184:325-35.
[3] Pottegård A, Morin L, Hallas J, Gerhard T, Winterstein AG, Perez-Gutthann S, et al. Where to begin? Thirty must-read papers for newcomers to pharmacoepidemiology. Pharmacoepidemiol Drug Saf. 2022;31:257-9.
[4] Suissa S. Immortal time bias in observational studies of drug effects. Pharmacoepidemiol Drug Saf. 2007;16:241-9.
[5] Morgan CJ. Landmark analysis: A primer. J Nucl Cardiol. 2019;26:391-3.
[6] Thomas L, Li F, Pencina M. Using Propensity Score Methods to Create Target Populations in Observational Clinical Research. Jama. 2020;323:466-7.
[7] Haneuse S, VanderWeele TJ, Arterburn D. Using the E-Value to Assess the Potential Effect of Unmeasured Confounding in Observational Studies. Jama. 2019;321:602-3.
[8] Arnold BF, Ercumen A. Negative Control Outcomes: A Tool to Detect Bias in Randomized Trials. Jama. 2016;316:2597-8.
