# Supplementary material for: Associations of Semaglutide With Skeletal Outcomes in People With Obesity, With and Without Type 2 Diabetes: A Target Trial Emulation
Source: Diabetes Obes Metab. 2026 Apr 20;28(7):5834–47. doi: 10.1111/dom.70786 (PMC13243989; doi:10.1111/dom.70786)
Supplement: Supplementary file 2 — Data S1: dom70786‐sup‐0002‐Supinfo2.docx. [file DOM-28-5834-s002.docx]

STROBE Statement—Checklist of items that should be included in reports of ***cohort studies***

|  | Item No | Recommendation |
| --- | --- | --- |
| **Title and abstract** | 1 | *(a) Indicate the study’s design with a commonly used term in the title or the abstract*  The title “Associations of Semaglutide with Skeletal Outcomes in People with Obesity, with and without Type 2 Diabetes: A Target Trial Emulation” includes “Target Trial Emulation,” clearly indicating the cohort study design. |
|  |  | *(b) Provide in the abstract an informative and balanced summary of what was done and what was found*  The abstract describes the study as a retrospective cohort using the TriNetX federated EHR network with propensity score matching (215 covariates). It reports the with-T2D cohort (n = 19,824–93,519 matched pairs) followed for 3 years and the without-T2D cohort (n = 10,323–56,225 pairs) for 2 years. Key findings: semaglutide was associated with lower MOF risk in the T2D cohort (HR 0.69–0.84 vs comparators) but not in the non-T2D cohort. |
| Introduction | | |
| Background/rationale | 2 | *Explain the scientific background and rationale for the investigation being reported*  Introduction: Obesity and T2D impact bone health through increased mechanical stress on weight-bearing joints and diabetes-related metabolic perturbations that alter bone density and microarchitecture. GLP-1 RAs have transformed adiposity reduction but long-term skeletal data remain scarce. Few head-to-head evaluations distinguish obesity alone from obesity with T2D, motivating this investigation. |
| Objectives | 3 | *State specific objectives, including any prespecified hypotheses*  Introduction: The study aimed to characterize associations between semaglutide initiation and long-term skeletal outcomes in people with obesity, stratified by T2D status, using a target trial emulation framework. The primary prespecified hypothesis was that semaglutide initiation would be associated with lower MOF hazard compared with active comparators in the T2D cohort. MOF was prioritized as the primary endpoint because it summarizes clinically relevant fracture burden. |
| Methods | | |
| Study design | 4 | *Present key elements of study design early in the paper*  Methods: Retrospective cohort study emulating two parallel target trials in the TriNetX US Collaborative Network, comparing skeletal outcomes after semaglutide initiation in people with obesity, stratified by T2D status. A new-user design required no prescription for the index drug or same-class medication during the 12 months before T₀. Intention-to-treat was the primary approach. Reporting followed STROBE guidelines. |
| Setting | 5 | *Describe the setting, locations, and relevant dates, including periods of recruitment, exposure, follow-up, and data collection*  Methods (Data Source and Study Design): The TriNetX US Collaborative Network, a federated database of de-identified records from approximately 120 million patients across 65 healthcare organizations in the United States. Eligibility windows: January 1, 2018 to May 31, 2025 for obesity with T2D; June 1, 2021 to May 31, 2025 for obesity without T2D (aligned with FDA approval dates of Ozempic December 2017 and Wegovy June 2021). The T2D cohort was followed for 3 years; the non-T2D cohort for 2 years. |
| Participants | 6 | *(a) Give the eligibility criteria, and the sources and methods of selection of participants. Describe methods of follow-up*  Methods (Cohort Construction): People with obesity initiating semaglutide were identified from prescription records. Exclusions: other/unspecified diabetes (E08, E09, E13), type 1 diabetes (E10), prior bariatric surgery or organ transplantation, HIV, and end-stage renal disease. Follow-up started at T₀ (first qualifying prescription) and continued under an ITT approach regardless of treatment discontinuation, switching, or augmentation, ending at the first occurrence of the outcome, death, loss to follow-up, or administrative end of follow-up. |
|  |  | *(b) For matched studies, give matching criteria and number of exposed and unexposed*  Methods (Statistical Analysis) and Results: 1:1 nearest-neighbor propensity score matching on 215 covariates with a caliper of 0.2 standard deviations of the logit propensity score; balance confirmed by standardized mean differences below 0.10. In the T2D cohort, matched cohorts ranged from 19,824 to 93,519 pairs per arm. In the non-T2D cohort, 10,323 to 56,225 pairs. Figure 1 provides a comprehensive flow diagram with matched pairs. |
| Variables | 7 | *Clearly define all outcomes, exposures, predictors, potential confounders, and effect modifiers. Give diagnostic criteria, if applicable*  Methods (Outcome Assessment and Covariates): Exposure: semaglutide initiation from prescription records. Primary outcome: major osteoporotic fracture (MOF) — composite of hip (S72), clinical vertebral (S32.0–S32.2), distal radius/ulna (S52.5–S52.6), proximal humerus (S42.2–S42.3), and fragility fractures (M80). Secondary: incident osteoporosis (M80–M81). Exploratory: knee OA (M17), hip OA (M16), gout (M10). Detailed ICD-10 codes in Supplementary Material. |
| Data sources/ measurement | 8* | *For each variable of interest, give sources of data and details of methods of assessment (measurement). Describe comparability of assessment methods if there is more than one group*  Methods: Diagnoses were ascertained via ICD-10-CM codes, medications from prescription records, and laboratory values from structured EHR fields within the TriNetX platform. The same diagnostic codes and data sources were used for both semaglutide and comparator groups. A directed acyclic graph (Figure S1) guided covariate selection. Negative control outcomes (dog bites, ganglion cysts, adhesive capsulitis, blepharitis, hernias, skin cancer) were included to probe residual confounding. Details in Supplementary Material. |
| Bias | 9 | *Describe any efforts to address potential sources of bias*  Methods (Covariates and Statistical Analysis): Propensity score matching on 215 covariates including demographics, lifestyle factors, comorbidities, concomitant therapies, laboratory measures, systemic glucocorticoid prescriptions, DXA utilization, healthcare utilization intensity, fall history, and prior traumatic fracture. Benjamini–Hochberg FDR correction across comparisons. E-values calculated for significant associations. Per-protocol analysis performed. Negative control outcomes assessed. Post-baseline mediators (weight change, glycemic trajectories) were not adjusted for to avoid blocking the pathway of interest. See also Supplementary Table 1. |
| Study size | 10 | *Explain how the study size was arrived at*  Methods and Results: Study size was determined by the available population within TriNetX meeting eligibility criteria. After applying inclusion/exclusion criteria and propensity score matching, the T2D cohort yielded 19,824 to 93,519 matched pairs per comparison, and the non-T2D cohort yielded 10,323 to 56,225 matched pairs. Figure 1 provides a comprehensive cohort selection flowchart showing numbers at each stage. |
| Quantitative variables | 11 | *Explain how quantitative variables were handled in the analyses. If applicable, describe which groupings were chosen and why*  Methods (Statistical Analysis): Continuous variables (BMI, HbA1c, eGFR, lipid values) entered the propensity score model directly. Pre-specified subgroups used clinically meaningful cut-points: eGFR (≥45 vs <45 mL/min/1.73 m²), HbA1c (≥7% vs <7%), BMI categories (<30, 30–34.9, 35–39.9, ≥40 kg/m²). Cox proportional hazards models estimated hazard ratios with 95% confidence intervals. Per-protocol analysis used cumulative 75-day refill intervals (30-day prescription plus 45-day permissible gap). |
| Statistical methods | 12 | *(a) Describe all statistical methods, including those used to control for confounding*  Methods: 1:1 nearest-neighbor propensity score matching (215 covariates, caliper 0.2 SD of logit PS). Cox proportional hazards models estimated HRs with 95% CIs. Benjamini–Hochberg procedure controlled FDR across all comparisons; both raw and adjusted p-values reported. E-values calculated for selected significant associations. Per-protocol analyses restricted to patients persistent on assigned treatment across cumulative 75-day refill intervals. ITT was the primary approach. Analyses run on the TriNetX platform (Java 11.0.16), with statistics in R 4.0.2. |
|  |  | *(b) Describe any methods used to examine subgroups and interactions*  Methods: Pre-specified subgroup analyses by eGFR (≥45 vs <45 mL/min/1.73 m²), HbA1c (≥7% vs <7%), BMI categories, cardiovascular comorbidities (hypertension, heart failure, ischemic heart disease), proteinuria, and background therapy (insulin, metformin, renin–angiotensin system inhibitors). Interaction p-values were reported (e.g., p for interaction = 0.03 for HbA1c <7% subgroup in semaglutide vs empagliflozin osteoporosis comparison). |
|  |  | *(c) Explain how missing data were addressed*  Methods: Complete-case analysis was used. Missing data were handled without imputation. The proportions of missing data for key continuous covariates before propensity score matching are reported in Table S2. Sensitivity sets with alternative completeness rules were also examined. |
|  |  | *(d) If applicable, explain how loss to follow-up was addressed*  Methods: Under the primary ITT estimand, follow-up started at T₀ and continued regardless of treatment discontinuation, switching, or augmentation, ending at the first occurrence of the outcome, death, loss to follow-up, or administrative end of follow-up. Cox proportional hazards models account for varying follow-up periods through censoring. Per-protocol analysis partially addresses adherence-related bias. |
|  |  | *(e) Describe any sensitivity analyses*  Methods: (1) Approval-aligned sensitivity analysis restricted each comparison to the later drug’s approval date. (2) Class-based sensitivity analysis replacing semaglutide with any GLP-1 RA (semaglutide, liraglutide, dulaglutide, exenatide). (3) Validated hip fracture endpoint confirmed by surgical procedure codes (CPT 27235, 27244, 27245; ICD-10-PCS 0QS6, 0QS7; PPV >95%). (4) Site-specific fracture analyses. (5) Composite endpoint of MOF plus all-cause mortality. (6) Global network and three-month landmark analyses. (7) Per-protocol analysis with 75-day grace periods. (8) Negative control outcomes. |
| Results | | |
| Participants | 13* | *(a) Report numbers of individuals at each stage of study—eg numbers potentially eligible, examined for eligibility, confirmed eligible, included in the study, completing follow-up, and analysed*  Results and Figure 1: Two populations with obesity were included. T2D cohort: compared against glucose-lowering agents or usual care over 3 years, with matched cohorts ranging from 19,824 to 93,519 pairs per arm. Non-T2D cohort: compared against anti-obesity medications or usual care over 2 years, with 10,323 to 56,225 matched pairs. After matching, mean age ranged 55.1–62.5 years, female 43.0%–59.2%, mean BMI 34.1–38.2 kg/m², mean HbA1c 7.1%–7.5%. Figure 1 provides a comprehensive flow diagram. |
|  |  | *(b) Give reasons for non-participation at each stage*  Figure 1: Detailed exclusion criteria and numbers at each stage, including exclusions for other/unspecified diabetes (E08, E09, E13), type 1 diabetes (E10), prior bariatric surgery or organ transplantation, HIV, and end-stage renal disease. Numbers excluded at each step are shown in the flow diagram. |
|  |  | *(c) Consider use of a flow diagram*  Figure 1: A comprehensive flow diagram is provided showing the cohort selection process from the TriNetX database through eligibility screening, exclusion criteria application, and propensity score matching for all nine comparison pairs. |
| Descriptive data | 14* | *(a) Give characteristics of study participants (eg demographic, clinical, social) and information on exposures and potential confounders*  Results and Tables S3–S10: Baseline characteristics after PSM are detailed, including age, sex, race, BMI, HbA1c, eGFR, comorbidities (hypertension, heart failure, ischemic heart disease, hyperlipidemia, kidney disease), concomitant medications (insulin, metformin, SGLT2 inhibitors, sulfonylureas, DPP-4 inhibitors, bisphosphonates, statins), healthcare utilization, fall history, prior traumatic fracture, and DXA utilization. All post-matching SMDs were below 0.10. |
|  |  | *(b) Indicate number of participants with missing data for each variable of interest*  Methods and Table S2: The proportions of missing data for key continuous covariates (BMI, HbA1c, eGFR, lipid values) before propensity score matching are reported in Supplementary Table S2. Complete-case analysis was used; sensitivity sets with alternative completeness rules were also examined. |
|  |  | *(c) Summarise follow-up time (eg, average and total amount)*  Results: The T2D cohort was followed for 3 years (with sensitivity analyses extending to longer periods); the non-T2D cohort was followed for 2 years. Kaplan–Meier survival curves (Figures 2–3) show temporal patterns of cumulative incidence across the follow-up period. |
| Outcome data | 15* | *Report numbers of outcome events or summary measures over time*  Results, Table 1, and Figures 2–3: MOF events, osteoporosis diagnoses, and exploratory outcomes (knee OA, hip OA, gout) are reported with cumulative incidence data across all treatment comparisons in both cohorts. Hazard ratios with 95% CIs are provided for each comparison. For example, semaglutide vs empagliflozin for MOF: HR 0.69 (95% CI 0.61–0.77); semaglutide vs glipizide: HR 0.72 (0.63–0.83); semaglutide vs usual care: HR 0.84 (0.76–0.93). |
| Main results | 16 | *(a) Give unadjusted estimates and, if applicable, confounder-adjusted estimates and their precision (eg, 95% confidence interval). Make clear which confounders were adjusted for and why they were included*  Results, Table 1: Hazard ratios with 95% CIs from Cox models after PSM on 215 covariates are reported. In the T2D cohort: semaglutide vs empagliflozin for MOF HR 0.69 (0.61–0.77), vs glipizide HR 0.72 (0.63–0.83), vs usual care HR 0.84 (0.76–0.93), all significant after FDR correction. Semaglutide vs sitagliptin HR 0.84 (0.70–0.99, raw p = 0.045) did not remain significant after FDR correction (adjusted p = 0.113). Both raw and FDR-adjusted p-values are reported. |
|  |  | *(b) Report category boundaries when continuous variables were categorized*  Methods and Results: BMI categories: <30, 30–34.9, 35–39.9, ≥40 kg/m². HbA1c thresholds: ≥7% vs <7%. eGFR categories: ≥45 vs <45 mL/min/1.73 m². These boundaries were pre-specified for subgroup analyses based on clinically meaningful cut-points. |
|  |  | *(c) If relevant, consider translating estimates of relative risk into absolute risk for a meaningful time period*  Results: Cumulative incidence rates are provided alongside hazard ratios in Kaplan–Meier curves (Figures 2–3). For example, in the validated hip fracture sensitivity analysis, semaglutide vs empagliflozin showed HR 0.45 (95% CI 0.30–0.67). Site-specific fracture HRs ranged from 0.52 (hip) to 0.77 (humerus). |
| Other analyses | 17 | *Report other analyses done—eg analyses of subgroups and interactions, and sensitivity analyses*  Results and Supplementary Tables S13–S22: Subgroup analyses by HbA1c, eGFR, BMI, cardiovascular comorbidities, proteinuria, and background therapy. Key finding: semaglutide showed stronger association with reduced osteoporosis risk among patients with HbA1c <7% (HR 0.46, 95% CI 0.24–0.85; p for interaction = 0.03) vs empagliflozin. Sensitivity analyses: class-level GLP-1 RA analysis confirmed consistent MOF associations; validated hip fracture (HR 0.45, 0.30–0.67 vs empagliflozin); per-protocol analysis consistent with ITT; global network and 3-month landmark analyses consistent with main findings. Figures S2–S7. |
| Discussion | | |
| Key results | 18 | *Summarise key results with reference to study objectives*  Discussion: Semaglutide initiation was associated with lower hazard of MOF (primary endpoint) in people with obesity and T2D. Within the T2D cohort, semaglutide showed lower MOF hazards across all four comparators (empagliflozin, sitagliptin, glipizide, usual care), consistent regardless of the reference drug. Among those without T2D, no significant MOF associations were observed. Osteoporosis and gout findings were generally non-significant. Exploratory osteoarthritis findings were inconsistent across comparators, possibly reflecting detection bias. |
| Limitations | 19 | *Discuss limitations of the study, taking into account sources of potential bias or imprecision. Discuss both direction and magnitude of any potential bias*  Discussion (Limitations): (1) Key fracture determinants (frailty, fall risk, physical activity, menopausal status, vitamin D, bone turnover markers, baseline BMD) not directly measurable in TriNetX; proxy variables used. E-value: unmeasured confounder would need 2.28-fold association with both treatment and outcome to explain the semaglutide–empagliflozin MOF result. (2) EHR data may under-capture mild OA; residual confounding from symptom-driven prescribing cannot be excluded. (3) Dosage variations, treatment adherence, and lifestyle changes could not be fully accounted for. (4) Concomitant metformin/pioglitazone use assessed only at baseline. (5) Different comparator classes in T2D vs non-T2D strata reflect prescribing practice differences; residual channeling bias may persist. (6) Outcome ascertainment relied on ICD-10 codes; non-differential misclassification would generally attenuate HRs toward the null. |
| Interpretation | 20 | *Give a cautious overall interpretation of results considering objectives, limitations, multiplicity of analyses, results from similar studies, and other relevant evidence*  Discussion: Results are interpreted as comparative associations under real-world treatment initiation, not as causal effects. Findings are consistent with preclinical literature on GLP-1–related bone biology and GLP-1 receptors on osteoblasts and osteoclasts. T2D-specific findings may reflect indirect skeletal benefits through improved glycemic control mitigating hyperglycemia-related AGE accumulation. Some associations may reflect chance despite FDR correction; the most consistent MOF findings (vs empagliflozin, glipizide, and usual care) were supported by per-protocol, site-specific, and validated hip fracture analyses. Findings are hypothesis-generating. |
| Generalisability | 21 | *Discuss the generalisability (external validity) of the study results*  Discussion: The TriNetX network includes approximately 120 million patients across 65 US healthcare organizations. However, semaglutide dosing patterns differ by indication (obesity regimens reach higher maintenance doses than T2D regimens). Different comparator classes in the T2D and non-T2D strata reflect real-world prescribing practice differences. Results should be interpreted within each drug comparison. For people with T2D and elevated fracture risk, the lower MOF hazards observed warrant further study in trials capturing dose, adherence, and bone imaging data. |
| Other information | | |
| Funding | 22 | *Give the source of funding and the role of the funders for the present study and, if applicable, for the original study on which the present article is based*  Funding: Taiwan’s National Science and Technology Council (NSTC 113-2314-B-040-026-MY2 and NSTC 114-2622-B-040-001) with supplementary funding from Chung Shan Medical University Hospital (CSH-2026-A-009, CSH-2026-C-030, CSH-2026-F-003). The study funder was not involved in the design of the study; the collection, analysis, and interpretation of data; writing the report; and did not impose any restrictions regarding publication. IRB approval: CS2-24004 and CS2-24100, Chung Shan Medical University Hospital. |

*Give information separately for exposed and unexposed groups.
